# Supplementary material for: Maintenance of physical activity level, functioning and health after non-pharmacological treatment of pelvic girdle pain with either transcutaneous electrical nerve stimulation or acupuncture: a randomised controlled trial
Source: BMJ Open. 2021 Oct 1;11(10):e046314. doi: 10.1136/bmjopen-2020-046314 (PMC8488730; doi:10.1136/bmjopen-2020-046314)
Supplement: Author's manuscript [file bmjopen-2020-046314.draft_revisions.pdf]

BMJ Open is committed to open peer review. As part of this commitment we make the peer review history of every article we publish publicly available.

When an article is published we post the peer reviewers' comments and the authors' responses online. We also post the versions of the paper that were used during peer review. These are the versions that the peer review comments apply to.

The versions of the paper that follow are the versions that were submitted during the peer review process. They are not the versions of record or the final published versions. They should not be cited or distributed as the published version of this manuscript.

BMJ Open is an open access journal and the full, final, typeset and author-corrected version of record of the manuscript is available on our site with no access controls, subscription charges or pay-per-view fees (http://bmjopen.bmj.com).

If you have any questions on BMJ Open's open peer review process please email info.bmjopen@bmj.com

BMJ Open

Maintenance of physical activity level, functioning and health after non-pharmacological treatment of pelvic girdle pain with either transcutaneous electric nerve stimulation or acupuncture. A randomized controlled trial

Journal:	*BMJ Open*
Manuscript ID	bmjopen-2020-046314
Article Type:	Original research
Date Submitted by the Author:	26-Oct-2020
Complete List of Authors:	Svahn Ekdahl, Annika; Sahlgrenska Academy, Department of Health and Rehabilitation; Physiotherapy<br>Olsen, Monika Fagevik; Sahlgrenska universitetssjukhuset, Physiotherapy; Sahlgrenska Academy, Department of Health and Rehabilitation; Physiotherapy<br>Jendman, Tove; Physiotherapy Clinic "I Rorelse"<br>Gutke, Annelie; Sahlgrenska Academy, Department of Health and Rehabilitation; Physiotherapy
Keywords:	PAIN MANAGEMENT, Maternal medicine < OBSTETRICS, Back pain < ORTHOPAEDIC & TRAUMA SURGERY, Musculoskeletal disorders < ORTHOPAEDIC & TRAUMA SURGERY

SCHOLARONE™
Manuscripts

I, the Submitting Author has the right to grant and does grant on behalf of all authors of the Work (as defined in the below author licence), an exclusive licence and/or a non-exclusive licence for contributions from authors who are: i) UK Crown employees; ii) where BMJ has agreed a CC-BY licence shall apply, and/or iii) in accordance with the terms applicable for US Federal Government officers or employees acting as part of their official duties; on a worldwide, perpetual, irrevocable, royalty-free basis to BMJ Publishing Group Ltd ("BMJ") its licensees and where the relevant Journal is co-owned by BMJ to the co-owners of the Journal, to publish the Work in this journal and any other BMJ products and to exploit all rights, as set out in our licence.

The Submitting Author accepts and understands that any supply made under these terms is made by BMJ to the Submitting Author unless you are acting as an employee on behalf of your employer or a postgraduate student of an affiliated institution which is paying any applicable article publishing charge ("APC") for Open Access articles. Where the Submitting Author wishes to make the Work available on an Open Access basis (and intends to pay the relevant APC), the terms of reuse of such Open Access shall be governed by a Creative Commons licence – details of these licences and which Creative Commons licence will apply to this Work are set out in our licence referred to above.

Other than as permitted in any relevant BMJ Author's Self Archiving Policies, I confirm this Work has not been accepted for publication elsewhere, is not being considered for publication elsewhere and does not duplicate material already published. I confirm all authors consent to publication of this Work and authorise the granting of this licence.

For peer review only

Maintenance of physical activity level, functioning and health after non-pharmacological treatment of pelvic girdle pain with either transcutaneous electric nerve stimulation or acupuncture. A randomized controlled trial

Annika Svahn Ekdahl[1], Monika Fagevik Olsén[1,2,] Tove Jendman[3], Annelie Gutke[1]

[1] Institute of Neuroscience and Physiology, Section of Health and Rehabilitation, Physiotherapy, Sahlgrenska Academy, University of Gothenburg, Gothenburg, Sweden

[2] Department of Physiotherapy, Sahlgrenska University Hospital, Gothenburg, Sweden

[3] Physiotherapy clinic "I Rörelse," Gothenburg, Sweden

Correspondence to:
Annika Svahn Ekdahl
University of Gothenburg
Institute of Neuroscience and Physiology
Department of Physiotherapy
Box 455
405 30 Gothenburg
Sweden
Phone: +46 31 786 5724 Mobile: +46 766 185724
E-mail: annika.svahn-ekdahl@neuro.gu.se

Word count: Abstract 300, Body of manuscript 2987

For peer review only - http://bmjopen.bmj.com/site/about/guidelines.xhtml

Abstract

Objective

To investigate if there are differences between acupuncture and transcutaneous electric nerve stimulation (TENS) as treatment for pelvic girdle pain (PGP) in pregnancy in order to manage pain and thus maintain health and functioning in daily activities and physical activity (PA).

Design

Randomized controlled trial

Setting and participants

Pregnant women (n=113) with clinically verified PGP in gestational weeks 12–28, recruited from maternity healthcare centers, randomized (1:1) into two groups. Exclusion criteria: Any obstetric complication, systemic disease or previous disorder that could contradict tests or treatment.

Interventions

The intervention consisted of either ten acupuncture sessions (two sessions per week) provided by a physiotherapist or daily home-based TENS during five weeks.

Primary outcome variables

Disability (Oswestry Disability Index), functioning (Patient Specific Functional Scale), work ability (Work Ability Index) and PA-level according to general recommendations.

Secondary outcome variables

Functioning related to PGP (Pelvic Girdle Questionnaire), evening pain intensity (Numeric Rating Scale, NRS), concern about pain (NRS), health (EuroQoL 5D), symptoms of

For peer review only

For peer review only - http://bmjopen.bmj.com/site/about/guidelines.xhtml

depression/catastrophizing (Edinburgh Postnatal Depression Scale/Coping Strategies Questionnaire).

Results

No clinically important differences were detected between the groups. Both groups managed to preserve their functioning and PA level at follow up. This may be due to significantly ($p<0.05$) reduced within groups evening pain intensity; acupuncture -0.96 (95% CI -1.91 to -0.01; p=0.049), TENS -1.29 (95% CI -2.13 to -0.44; p=0.003) and concern about pain; acupuncture -1.44 (95% CI -2.31 to -0.57; p=0.0012), TENS -1.99 (CI -2.81 to -1.17; $p<0.0001$).

Conclusion

Treating PGP with acupuncture or TENS resulted in maintenance of functioning and physical activity and also less pain and concern about pain. Either intervention could be recommended as a non-pharmacological alternative for pain relief and may enable pregnant women to stay active.

Trial registration: In "FoU i Sverige" (R&D in Sweden) May 2013, No. 12726. Enrollment of participants started in 2014.

For peer review only

For peer review only - http://bmjopen.bmj.com/site/about/guidelines.xhtml

Article Summary

Strengths and limitations of this study

- All participants had clinically verified PGP during pregnancy.
- Interventions were evaluated both in the 2nd and 3rd trimester and few minor side effects were reported.
- In the acupuncture group, 93 % completed the assigned 10 sessions and in the TENS group the women used the TENS device at home for mean 26 out of 35 possible days.
- It is possible that the participants did not use TENS according to instructions, as this was self-reported.
- The study design, where pregnant women had to travel to the clinic for treatment and/or assessment, may have affected the ability to recruit women with more severe PGP to the study.

For peer review only

For peer review only - http://bmjopen.bmj.com/site/about/guidelines.xhtml

Introduction

Pelvic girdle pain (PGP) and/or low back pain (LBP) is reported internationally by over 50% of pregnant women.[1-3] PGP has musculoskeletal origin and is localized to the area between the posterior iliac crest and the gluteal fold, most commonly around the sacroiliac joints and/or pubic bone, sometimes with radiating pain in the thighs.[4] Limitations in daily functioning and walking difficulties, especially a feeling of "catching" of the leg are common symptoms.[2] These symptoms differ from those associated with LBP, which is defined as pain in the area between the lower ribs and the iliac crest, with or without radiating pain to the legs, and is often described as worse when sitting.[5]

The etiology of PGP is multifactorial. Increased pregnancy-related ligamentous laxity may result in altered mechanics in the pelvic joints.[6] Pain may occur if these alterations cannot be compensated for by neuromuscular control.[4] PGP most commonly occurs around gestation week 18, but can occur at any time during pregnancy. Among women who experience PGP during pregnancy, about 20% suffer from persistent pain 4 months postpartum.[7] Previous long-term studies have reported that approximately 10% suffer from remaining PGP more than 11 years postpartum.[8, 9] Experiencing PGP during pregnancy is a risk factor for having PGP also in a next pregnancy.[2]

Pregnant women are advised to follow the general recommendations for physical activity (PA), of at least 150 minutes of moderate intensity or at least 75 minutes of high intensity PA per week or a combination of both.[10] Few pregnant women reach this level of PA and a common cause is PGP.[11] A high level of pain during pregnancy results in more severe limitations in daily life and less PA.[12]

There is a higher rate of sick leave among pregnant women compared to age-matched, non-pregnant women and a major reason is PGP and/or LBP.[13-15] Studies show a reduced risk

For peer review only - http://bmjopen.bmj.com/site/about/guidelines.xhtml

For peer review only

for sick leave if the woman can continue to stay active and exercise throughout her pregnancy.[16, 17] Women with PGP and/or LBP have an increased risk of prenatal anxiety, depressive symptoms and postpartum depression compared to healthy pregnant women.[18, 19] If pregnant women maintains the recommended levels of PA, both the odds of getting prenatal depression and the severity of it is reduced.[20] Furthermore, there is a risk in pregnancy for complications such as hypertension, diabetes and preeclampsia and this risk is reduced if the woman stays active.[21]

The use of medication in pregnancy has increased over the years [22, 23] with a risk for overuse of analgesics among women having PGP.[24] Therefore, it is important to provide efficient treatment alternatives. The best evidence non-pharmacological treatments for PGP are acupuncture, a stabilizing pelvic belt, and physical exercise.[25, 26] There is, however, limited evidence for the use of transcutaneous electrical nerve stimulation (TENS),[25-27] even if it is widely used in physiotherapy clinical practice. The physiological mechanisms of acupuncture and TENS are similar. Both peripheral and central mechanisms of pain control are stimulated, as per the gate control theory, activating descending pathways for pain control and release of endogenous opioids.[28, 29] No specific adverse effects regarding use in pregnancy are reported for any of the interventions mentioned here.[30]

The aim of this study was to investigate if there are differences between acupuncture and TENS as treatments for PGP in order to manage pain and thus maintain health and functioning in daily activities and PA.

Methods

A randomized, controlled design was used to compare acupuncture and TENS as pain relief for PGP in pregnancy. The decision to randomize into two different groups was made based

For peer review only

on ethical considerations. It is not ethical to withhold evidence based treatment, therefore the experimental treatment (TENS) was given to one group and acupuncture, which is known to be effective for PGP [25, 26], to an active control group.[31] The study was approved by the regional ethical review board in Gothenburg, Sweden (No. 308-13).

Setting and Participants

The data collection took place 2014–2018 in two Swedish cities. Two independent physiotherapists located in each city conducted tests and treatment. Women with PGP were briefly informed about the study at Maternity Health Care Centers and those who were interested were contacted by the independent test leader for detailed information and for initial screening for inclusion.

Inclusion criteria

- Single fetus pregnancy
- Pregnancy-related PGP or combined PGP/LBP in gestational weeks 12–28
- Pain located distally/laterally of L5/S1, over the buttocks, and/or the pubic bone, verified by ≥2 positive pain provocation tests or a positive active straight leg raise (ASLR)
- A score of ≥20% on the Oswestry Disability Index (ODI) and/or ≤6 in one self-chosen activity of the Patient-Specific Functional Scale (PSFS)
- Ability to read and understand Swedish

Exclusion criteria

- Previous fracture, surgery, or malignant disease in the back, pelvis, or hips
- Any systemic disease or obstetric complication that contraindicates treatment or tests
- Contraindications for TENS; pacemaker; decreased sensibility in the treatment area
- Contraindications for acupuncture; treatment with anticoagulants

For peer review only - http://bmjopen.bmj.com/site/about/guidelines.xhtml

- Start of other treatment during the study period

Those eligible for participation underwent a clinical examination to verify PGP and answered the baseline questionnaire. All participants gave oral and written consent. The clinical examination consisted of a reliable procedure, which included a neurological examination, hip rotation range of motion test, pelvic pain provocation tests, a brief mechanical assessment of the lumbar spine and the ASLR test.[32] The examination also included a single-leg balance test.[33]

Randomization and Interventions

Before start of intervention, the participants each drew a sealed, opaque envelope, prepared from a computer-generated list by an independent research assistant, for assignment to either the acupuncture or TENS group (1:1).

The intervention lasted for 5 weeks in both groups. All women got written general advice on how to manage PGP in addition to the assigned treatment. The women randomized to acupuncture received ten sessions of 30 minutes each. It was decided to shorten the treatment if any woman experienced enough pain relief in fewer than ten sessions. The acupuncture points were chosen, based on the women's symptoms, from a protocol tested elsewhere.[34] After insertion, the needles were manually stimulated twice during each treatment session.

The women randomized to TENS received oral and written instructions and then used the TENS device (Cefar Primo PRO; DJO Nordic AB, Malmö, Sweden) at home for at least 30 minutes per day for 5 weeks, placing the electrodes in the area of pain and using high frequency stimulation (80 Hz). Each woman met the physiotherapist on a follow-up visit after 1 week to discuss the treatment and to adjust electrode placement if needed. After 3 weeks, one follow-up telephone call to each woman was made to ensure that the intervention was working well. After 5 weeks the participants made a final visit to the physiotherapist.

For peer review only - http://bmjopen.bmj.com/site/about/guidelines.xhtml

Outcomes and Follow-up

Because of the present study's focus on functioning, a broad spectrum of outcome variables was included.

Primary outcomes

- Disability, measured by ODI, range 0–100%, where 0% = no disability.[35]
- Functional status, assessed by the PSFS. Two self-chosen everyday activities rated on a numeric rating scale (NRS) of 0–10; 10 = can perform the activity unrestrictedly or as before the onset of PGP.[36]
- Work ability, measured by one question from the Work Ability Index (WAI). Current work ability marked on a NRS of 0–10; 10 = work ability at its best.[37]
- Physical activity level, number of days/week with activity for 30 minutes at moderate intensity and/or activity at high intensity for 20 minutes.[38]

Secondary outcomes

- Daily function, measured by the Pelvic Girdle Questionnaire (PGQ). A total score and scores for the subscales of "Activity" and "Symptoms", where 0% = no disability.[39, 40]
- Evening pain intensity at its worst, measured by a NRS of 0–10; 10 = worst pain,[41] and by a pain drawing to illustrate the pain location.
- Concern about pain, assessed using a NRS of 0–10; 10 = extremely concerned.[42]
- Health, measured by the EuroQol 5-dimension questionnaire (EQ5D), a total score of 1.0 indicates full health. Participants also marked their current health status on the EuroQol vertical visual analog scale (EQ VAS); 100 = best imaginable health state.[43]

For peer review only - http://bmjopen.bmj.com/site/about/guidelines.xhtml

- Symptoms of depression, measured by the Edinburgh Postnatal Depression Scale (EPDS), range 0-30. A score >13 indicates symptoms of depression that require further examination.[44, 45]
- Catastrophizing beliefs via the subscale “Catastrophizing” (from the Coping Strategies Questionnaire, CSQ-CAT) range 0-36; 0 = no catastrophizing beliefs.[46, 47]
- Questions were also asked about use of analgesics, sick leave, height/weight, education level, family income, and possible side effects of the treatment.

After the intervention period the clinical examination was repeated by the independent test-leader, who was blinded to the treatment allocation, and the participants completed a follow-up questionnaire.

Patient and public involvement

No patients or members of the public was involved in design, conduction or interpretation of the study. Patient satisfaction was measured at follow up.

Statistical analysis

Sample size

With a β-level of 80%, an α-level of 5%, and a difference in functioning between groups of 10%, measured using the ODI, a sample size of at least 30 women/group was required. As the dropout rate for the postpartum follow-up and the planned 3.5 year long-term follow-up was calculated to be 25% and 50%, respectively, the plan was to include 60 women in each group.

Analysis

The analysis was done according to intention to treat (ITT), with the use of multiple imputation with 50 imputation datasets generated followed by one analysis of each of these 50 datasets followed by a pooled analysis of the 50 tests. The imputation of each value was

For peer review only

For peer review only - http://bmjopen.bmj.com/site/about/guidelines.xhtml

performed by Fully Conditional Specifications. For continuous variables T-test was applied due to central limit theorem and for dichotomous variables change in proportion was applied. A Full Analysis Set (FAS) was also performed, using Fischer's non-parametric permutation test, including only those women who completed the intervention. Change from baseline to follow up is expressed as mean (SD)/ median (min; max)/ 95% CI for mean. For dichotomous variables, the Chi Square-test was used. The level of statistical significance was set to $p<0.05$. The analysis was made using IBM SPSS Statistics v. 24 (IBM Corp., Armonk, NY, USA) and SAS v. 9.4 (SAS Institute, Cary, NC, USA).

Results

Altogether 15 women, who fulfilled the inclusion criteria, were excluded after the baseline test due to difficulties in finding time for treatment or they did not show up for randomization (Fig. 1). A total of 113 women were randomized into two groups (Fig. 1). No relevant differences between the intervention groups were found at baseline. At baseline, women in both groups reported pain in their pelvic and/or lumbar region during the last 4 weeks, which affected their daily activities for more than 1 day/week. (Table 1).

Table. 1 Descriptive data

Variable	Acupuncture n=56	TENS n=57	Excluded after baseline-test n=15
**Maternal age, mean (SD), median (min; max)**	30.5 (3.96)<br>30.0 (20.0; 38.0)	31.1 (4.38)<br>32.0 (22.0; 43.0)	31.9 (3.92)<br>32.0 (27.0; 40.0)
**Gestational weeks, mean (SD), median (min; max)**	20.9 (5.32)<br>21.5 (11.0; 28.0)	20.8 (5.18)<br>22.0 (10.0; 28.0)	22.4 (5.17)<br>24.0 (13.0; 28.0)
**BMI, mean (SD), median (min; max)**	25.6 (4.53)<br>25.0 (19.5; 41.8)	26.5 (3.87)<br>26.2 (19.7; 35.0)<br>*n=55*	27.6 (5.83)<br>27.6 (18.2; 41.0)
**Parity, n (%)**			
0	21 (37.5)	22 (38.6)	6 (40.0)
1	30 (53.6)	28 (49.1)	5 (33.3)
≥2	5 (8.9)	7 (12.3)	4 (26.7)
**Education level, n (%)**			
Elementary school	1 (1.8)	2 (3.5)	0
High school	12 (22.4)	20 (35.1)	3 (20.0)
Higher education	43 (76.8)	35 (61.4)	12 (80.0)
**Occupation, n (%)**			
Paid employment	47 (83.9)	47 (82.5)	14 (93.3)

For peer review only - http://bmjopen.bmj.com/site/about/guidelines.xhtml

Not in paid employment	3 (5.4)	3 (5.3)	0
Parental leave	0	2 (3.5)	0
Combination	2 (3.6)	3 (5.3)	0
Other	4 (7.1)	2 (3.5)	1 (6.7)
**Sick leave, n (%)**	9 (16.1)	7 (12.3)	3 (20.0)
**Manage financially, n (%)**			
Very well	22 (39.3)	34 (59.6)	7 (46.7)
Quite well	30 (53.6)	21 (36.8)	7 (46.7)
Neither well or badly	4 (7.1)	2 (3.5)	1 (6.7)
Quite badly	0	0	0
Very badly	0	0	0
**LBP/PGP last 4 weeks, n (%)**	56 (100)	57 (100)	15 (100)
**Pain affects daily activities, n (%)**	53 (94.6)	57 (100)	15 (100)
**Pain how often, n (%)**			
Some days	3 (5.4)	7 (12.3)	0
Most days	16 (28.6)	19 (33.3)	8 (53.3)
Every day	37 (66.1)	31 (54.4)	7 (46.7)
**Use of analgesics, n (%)**	6 (10.9)	3 (5.3)	0
**ODI, mean (SD), median (min; max)**	*n=55*<br>34.5 (13.19)<br>34.0 (8.0; 68.0)	30.1 (11.82)<br>30.0 (8.0; 60.0)	32.5 (12.22)<br>32.0 (12.0; 52.0)
**PSFS NRS, mean (SD), median (min; max)**	2.8 (1.46)<br>2.5 (0; 7.0)	3.2 (1.55)<br>3.0 (0; 7.5)	3.9 (2.33)<br>3.0 (0.5; 9.0)
**Work ability NRS, mean (SD), median (min; max)**	6.2 (2.36)<br>7.0 (0; 10.0)	6.7 (2.41)<br>7.0 (0; 10.0)	5.7 (3.02)<br>6.0 (0; 9.0)
**Recommended PA; yes, n (%)**	*n=55*<br>13 (23.2)	*n=56*<br>14 (24.6)	6 (40)
**PGQ total, mean (SD), median (min; max)**	49.3 (17.70)<br>50.7 (8.0; 90.7)	47.0 (12.91)<br>45.6 (22.2; 82.6))	53.2 (18.1)<br>60.0 (20.0; 77.3)
**PGQ activity, mean (SD), median (min; max)**	*n=55*<br>48.1 (17.76)<br>50.0 (6.7; 90.0)	*n=56*<br>46.3 (13.55)<br>45.3 (24.6; 82.5)	52.1 (18.63)<br>58.3 (16.7; 76.7)
**PGQ symptom, mean (SD), median (min; max)**	*n=55*<br>51.5 (17.17)<br>53.3 (13.3; 93.3)	*n=56*<br>49.4 (13.27)<br>46.7 (13.3; 73.3)	57.1 (15.98)<br>60.0 (26.67; 80.0)
**Evening pain intensity NRS, mean (SD), median (min; max)**	*n=55*<br>6.8 (1.95)<br>7.0 (2.0; 10.0)	6.6 (1.56)<br>7.0 (3.0; 9.0)	7.4 (1.56)<br>8.0 (4.0; 9.0)
**Concern about pain NRS, mean (SD), median (min; max)**	*n=55*<br>4.2 (2.44)<br>5.0 (0; 10.0)	4.6 (2.34)<br>5.0 (0; 10.0)	4.7 (2.69)<br>5.0 (0; 10.0)
**EQ 5D, mean (SD), median (min; max)**	0.55 (0.28)<br>0.69 (-0.13; 0.85)	0.63 (0.19)<br>0.69 (-0.02; 0.89)	0.55 (0.25)<br>0.66 (0.06; 0.8)
**EQ VAS, mean (SD), median (min; max)**	61.1 (16.27)<br>65.0 (20.0; 90.0)	*n=55*<br>64.4 (17.17)<br>70.0 (17.0; 97.0)	60.1 (12.69)<br>60.0 (40.0; 80.0)
**EPDS score, mean (SD), median (min; max)**	7.6 (4.11)<br>7.0 (1.0; 19.0)	8.0 (4.97)<br>8.0 (0; 18.0)	7.6 (3.92)<br>7.5 (2.0; 14.0)
**CSQ-CAT, mean (SD), median (min; max)**	*n=54*<br>8.0 (6.65)<br>7.0 (0; 27.0)	*n=56*<br>6.7 (4.98)<br>7.0 (0; 24.0)	8.2 (6.41)<br>9.0 (0; 20.0)

TENS, Transcutaneous Electric Nerve Stimulation; SD, Standard Deviation; BMI, Body Mass Index (kg/m²); NRS, Numeric Rating Scale; LBP, Low Back Pain; PGP, Pelvic Girdle Pain
ODI, Oswestry Disability Index; PSFS, Patient-Specific Functional Scale (PSFS); PGQ, Pelvic Girdle Questionnaire EQ 5D, EuroQol 5 Dimensions; EQ VAS, EuroQol vertical visual analog scale; EDPS, Edinburgh Postnatal Depression Scale; CSQ-CAT, Coping Strategies Questionnaire subscale "Catastrophizing"

For peer review only - http://bmjopen.bmj.com/site/about/guidelines.xhtml

The sacroiliac joint was the most common pain location (93%) (Table 2).

Table. 2 Clinical tests to verify pelvic girdle pain

Variable	Acupuncture n=56	TENS n=57	Excluded after baseline test n=15
**Reported pubic pain, n (%)**			
No	20 (35.7)	20 (35.1)	5 (33.3)
Yes	36 (64.3)	37 (64.9)	10 (67.7)
**Reported sacroiliac pain, n (%)**			
No	4 (7.1)	4 (7.0)	1 (6.7)
Yes	52 (92.9)	53 (93.0)	14 (93.3)
**Localization sacroiliac pain, n (%)**			
Unilateral	6 (11.5)	7 (13.0)	2 (15.4)
Bilateral	46 (88.5)	47 (87.0)	11 (84.6
	n=52	*n=54*	*n=13*
**Positive MAT-test, n (%)**			
No	29 (51.8)	29 (51.8)	8 (53.3)
Yes	27 (48.2)	27 (48.2)	7 (46.7)
		n=56	
**Positive 4P-test, n (%)**			
No	6 (10.7)	2 (3.5)	1 (6.6)
Yes, unilateral	9 (16.1)	16 (28.1)	4 (26.7)
Yes, bilateral	41 (73.2)	39 (68.4)	10 (66.7)
**Positive SIJ-separation, n (%)**			
No	24 (42.9)	24 (42.1)	5 (33.3)
Yes	32 (57.1)	33 (57.9)	10 (66.7)
**Positive SIJ-compression, n (%)**			
No	42 (75.0)	38 (66.7)	10 (66.7)
Yes	14 (25.0)	19 (33.3)	5 (33.3)
**Positive sacrum ventral-test, n (%)**			
No	29 (51.8)	31 (54.4)	8 (53.3)
Yes	27 (48.2)	26 (45.6)	7 (46.7)
**Positive ASLR-test, n (%)**			
No	10 (17.8)	8 (14)	4 (26.7)
Yes, unilateral	15 (26.8)	19 (33.4)	3 (20.0)
Yes, bilateral	31 (55.4)	30 (52.6)	8 (53.3)

***TENS**= Transcutaneous electric nervous stimulation; **MAT**= Pulling a mat-test; **4P**: Posterior Pelvic Pain Provocation-test; **SIJ**= Sacro-iliac joint; **ASLR**= Active straight leg raise*

The women in both groups reported that their work ability was affected and few of them (acupuncture 23.2%; TENS 24.6%) met the general recommendations for PA. The consumption of analgesics was low in both groups (Table 1).

For peer review only - http://bmjopen.bmj.com/site/about/guidelines.xhtml

For acupuncture, the mean number (min;max) of treatment sessions was 9.9 (9;10) and women in the TENS group used the device for mean 26 (3;37) out of 35 days. In the acupuncture group, 82.5% experienced positive effects of the treatment compared to 60% in the TENS group (p=0.032). At follow up, 9 of 41 women in the acupuncture group and 6 of 45 women in the TENS group reported some side effects during the intervention (Fig. 2).

Between group comparisons

No mean differences between groups were detected regarding the primary (disability, functioning, work ability, PA) or secondary outcome variables. Minor differences between the ITT and FAS were found (Table 3, 4).

Table 3. Change within groups and difference in change between the groups Acupuncture and TENS (ITT-analysis, multiple imputation of 50 imputed datasets)

	Acupuncture		TENS		Difference between groups	
**Outcomes**	**Mean change (95% CI)**	**p-value**	**Mean change (95% CI)**	**p-value**	**Mean difference (95% CI)**	**p-value**
**ODI**	0.44 (-3.74 to 4.61)	0.84	3.53 (-0.79 to 7.85)	0.11	-3.09 (-9.36 to 3.17)	0.33
**PSFS**	0.82 (0.01 to 1.63)	0.048	-0.16 (-0.79 to 0.47)	0.62	0.98 (-0.03 to 1.99)	0.058
**Work ability NRS**	-1.39 (-2.54 to -0.24)	0.018	-1.27 (-1.92 to -0.62)	0.0001	-0.12 (-1.44 to 1.19)	0.86
**Recommended PA**	-0.03 (-0.20 to 0.14)	0.72	-0.03 (-0.20 to 0.13)	0.68	-0.01 (-0.13 to 0.11)	0.87
**PGQ total**	-0.57 (-6.43 to 5.29)	0.85	3.08 (-2.28 to 8.43)	0.26	-3.65 (-11.46 to 4.16)	0.36
**PGQ activity**	0.37 (-5.69 to 6.44)	0.90	3.54 (-1.94 to 9.03)	0.20	-3.17 (-11.14 to 4.80)	0.43
**PGQ symptoms**	-1.34 (-8.33 to 5.65)	0.71	1.73 (-4.12 to 7.58)	0.56	-3.07 (-12.17 to 6.04)	0.51
**Evening pain NRS**	-0.96 (-1.91 to -0.01)	0.049	-1.29 (-2.13 to -0.44)	0.003	0.33 (-0.99 to 1.64)	0.62
**Concern about pain NRS**	-1.44 (-2.31 to -0.57)	0.0012	-1.99 (-2.81 to -1.17)	<.0001	0.55 (-0.64 to 1.73)	0.36
**EQ 5D**	0.04 (-0.07 to 0.14)	0.51	-0.10 (-0.21 to 0.00)	0.059	0.14 (-0.01 to 0.28)	0.061

For peer review only - http://bmjopen.bmj.com/site/about/guidelines.xhtml

**EQ 5D vertical VAS**	-1.18 (-7.72 to 5.37)	0.72	-1.66 (-8.79 to 5.48)	0.65	0.48 (-9.29 to 10.25)	0.92
**EPDS**	-0.40 (-2.07 to 1.28)	0.64	-1.25 (-2.77 to 0.27)	0.11	0.85 (-1.36 to 3.07)	0.45
**CSQ-CAT**	-2.50 (-5.43 to 0.43)	0.095	-1.21 (-2.68 to 0.25)	0.10	-1.29 (-4.55 to 1.98)	0.44

TENS, Transcutaneous Electric Nerve Stimulation; SD, Standard Deviation; BMI, Body Mass Index (kg/m²); NRS, Numeric Rating Scale; LBP,Low Back Pain; PGP, Pelvic Girdle Pain.
ODI, Oswestry Disability Index; PSFS, Patient-Specific Functional Scale; PGQ, Pelvic Girdle Questionnaire EQ 5D, EuroQol 5 Dimensions; EQ VAS, EuroQol vertical visual analog scale; EDPS, Edinburgh Postnatal Depression Scale; CSQ-CAT, Coping Strategies Questionnaire subscale "Catastrophizing"

For peer review only

Table 4. Change within groups and difference in change between the groups Acupuncture and TENS (Full analysis set, all available data)

	Acupuncture (n=41)				TENS (n=45)				Difference between groups	
**Outcome**	**Baseline<br>Mean (SD)<br>Median (Min; Max)**	**Follow up<br>Mean (SD)<br>Median (Min; Max)**	**Mean change (SD)<br>Median (Min; Max)<br>(95% CI for Mean)*<br>n=**	**p-value**	**Baseline<br>Mean (SD)<br>Median (Min; Max)**	**Follow up<br>Mean (SD)<br>Median (Min; Max)**	**Mean change (SD)<br>Median (Min; Max)<br>(95% CI for Mean)*<br>n=**	**p-value**	**Mean difference **<br>(95% CI)*****	**p-value**
**ODI**	32.59 (11.40)<br>34.00 (8.0; 60.0)	33.07 (13.41)<br>34.0 (10.0; 66.0)	0.49 (11.89)<br>2 (-22; 26)<br>(-3.20; 4.44)<br>n=41	0.80	29.07 (10.86)<br>28.0 (8.0; 60.0)	32.93 (15.24)<br>32.0 (2.0; 64.0)	3.87 (12.36)<br>4 (-28; 34)<br>(0.18; 7.68)<br>n=45	0.043	-3.38 (-8.53; 1.88)	0.21
**PSFS**	2.74 (1.54)<br>2.5 (0; 7.0)	3.63 (2.34)<br>3.5 (0; 10.0)	0.89 (2.45)<br>0 (-3; 6.5)<br>(0.11; 1.67)<br>n=41	0.025	3.16 (1.45)<br>3.0 (0.5; 7.5)	3.14 (1.75)<br>3.0 (0; 8.0)	-0.017 (1.85)<br>0 (-4.5; 4.5)<br>(-0.57; 0.54)<br>n=45	0.95	0.91 (0.00; 1.85)	0.053
**Work ability NRS**	6.45 (2.39)<br>7.0 (0; 10.0)	5.34 (3.11)<br>6.0 (0; 10.0)	-1.10 (3.13)<br>-1 (-8; 6)<br>(-2.12; -0.11)<br>n=40	0.036	7.0 (2.28)<br>7.0 (0; 10.0)	5.67 (2.54)<br>6.0 (0; 10)	-1.33 (1.92)<br>-1 (-6; 2)<br>(-1.91; -0.76)<br>n=45	<.0001	0.23 (-0.88; 1.31)	0.70
**Recommended PA; n (%)**	9 (22.0)	8 (19.5)	-0.02 (0.42)<br>0 (-1; 1)<br>(-0.15; 0.11)<br>n=41	1.00	10 (22.2)	9 (20.0)	-0.02 (0.40)<br>0 (-1; 1)<br>(-0.14; 0.10)<br>n=45	1.00	-0.002 (-0.17; 0.17)	1.00
**PGQ total**	46.68 (16.30)<br>48.64 (8.0; 89.72)	47.30 (16.32)<br>51.02 (12.0; 80.0)	0.82 (16.26)<br>3 (-34.67; 32)<br>(-4.51; 6.18)<br>n=39	0.76	45.25 (11.76)<br>42.16 (22.22; 69.33)	48.30 (16.74)<br>47.82 (12.50; 81.94)	3.84 (14.49)<br>2.05 (-38.17; 34.67)<br>(-0.48; 8.17)<br>n=44	0.086	-3.03 (-9.74; 3.63)	0.37
**PGQ activity**	44.93 (15.72<br>48.33 (6.67; 75.0)	47.02 (17.10)<br>49.12 (11.67; 78.94)	2.37 (15.65)<br>3.33 (-31.76; 31.67)<br>(-2.77; 7.44)<br>n=39	0.35	44.56 (12.16)<br>42.72 (24.56; 70.0)	48.00 (17.05)<br>46.67 (10.52; 82.45)	4.24 (14.81)<br>3.42 (-37.81; 33.33)<br>(-0.21; 8.66)<br>n=44	0.064	-1.87 (-8.50; 4.76)	0.58
**PGQ symptoms**	49.83 (16.40)<br>53.33 (13.33; 86.67)	48.42 (18.10)<br>48.33 (13.33; 86.67)	-1.45 (20.67)<br>0 (-53.33; 33.33)<br>(-8.24; 5.21)<br>n=39	0.66	48.00 (13.38)<br>46.67 (13.33; 73.33)	49.48 (18.12)<br>46.667 (0; 86.67)	1.48 (17.69)<br>6.67 (-40; 40)<br>(-3.86; 6.67)<br>n=45	0.58	-2.93 (-11.33; 5.56)	0.50
**Evening pain NRS**	6.82 (1.8)<br>7.0 (2.0; 9.5)	5.58 (2.26)<br>6.0 (1.0; 9.0)	-1.26 (2.64)<br>-1 (-7; 4)<br>(-2.12; -0.40)<br>n=39	0.0052	6.60 (1.5)<br>7.0 (3.0; 9.0)	5.33 (2.36)<br>6.0 (0; 9.0)	-1.27 (2.27)<br>-1 (-9; 3)<br>(-1.94; -0.60)<br>n=45	0.0002	0.01 (-1.06; 1.07)	1.00

For peer review only

For peer review only - http://bmjopen.bmj.com/site/about/guidelines.xhtml

**Concern about pain NRS**	3.95 (2.24)<br>5.0 (0; 8.0)	2.43 (2.15)<br>2.0 (0; 7.0)	-1.45 (2.43)<br>-2 (-7; 6)<br>(-2.25; -0.68)<br>n=40	0.0007	4.62 (2.41)<br>5.0 (0; 10.0)	2.62 (2.20)<br>2.0 (0; 8.0)	-2.00 (2.52)<br>-2 (-8; 2)<br>(-2.77; -1.25)<br>n=45	<.0001	0.55 (-0.52; 1.63)	0.32
**EQ 5D**	0.58 (0.25)<br>0.69 (-0.13; 0.85)	0.62 (0.23)<br>0.69 (-0.07; 0.88)	0.03 (0.32)<br>0 (-0.76; 0.75)<br>(-0.08; 0.13)<br>n=39	0.61	0.65 (0.18)<br>0.69 (-0.02; 0.89)	0.55 (0.29)<br>0.66 (-0.13; 1.0)	-0.10 (0.31)<br>-0.04 (-0.91; 0.61)<br>(-0.20; -0.01)<br>n=44	0.036	0.13 (-0.01; 0.27)	0.073
**EQ 5D vertical VAS**	62.24 (16.39)<br>65.0 (30.0; 90.0)	63.17 (16.38)<br>65.0 (25.0; 90.0)	0.93 (20.274)<br>0 (-47; 50)<br>(-5.60; 7.28)<br>n=41	0.78	66.20 (15.16)<br>70.0 (27.0; 95.0)	63.78 (19.51)<br>70.0 (19.0; 90.0)	-2.42 (19.75)<br>-1 (-55; 47)<br>(-8.25; 3.45)<br>n=45	0.42	3.35 (-5.31; 11.75)	0.45
**EPDS**	6.83 (3.99)<br>6.0 (1.0; 15.0)	6,15 (4.73)<br>5.0 (0; 15.0)	-0.85 (4.27)<br>-1 (-10; 11)<br>(-2.25; 0.52)<br>n=39	0.24	7.38 (4.91)<br>6.0 (0; 18.0)	6.27 (4.93)<br>5.0 (0; 21.0)	-1.11 (4.46)<br>0 (-12; 9)<br>(-2.43; 0.24)<br>n=45	0.11	0.27 (-1.65; 2.17)	0.79
**CSQ-CAT**	7.15 (5.98)<br>6.0 (0; 27)	4.49 (5.44)<br>2.0 (0; 18.0)	-2.60 (6.00)<br>-2.5 (-15; 12)<br>(-4.50; -0.70)<br>n=40	0.010	6.07 (4.83)<br>6.0 (0; 24.0)	5.11 (5.84)<br>4.0 (0; 28.0)	-1.09 (4.50)<br>-1 (-10; 17)<br>(-2.44; 0.27)<br>n=44	0.12	-1.51 (-3.82; 0.78)	0.20

* For comparison within groups the Fisher´s Non-Parametric Permutation Test for matched pairs was used.
** For comparison between groups the Fisher's Non Parametric Permutation Test was used for continuous variables.
*** The confidence interval for then mean difference between groups is based on Fisher's Non-Parametric Permutation Test.

TENS, Transcutaneous Electric Nerve Stimulation; SD, Standard Deviation; BMI, Body Mass Index (kg/m²); NRS, Numeric Rating Scale; LBP, Low Back Pain; PGP, Pelvic Girdle Pain ODI, Oswestry Disability Index; PSFS, Patient-Specific Functional Scale (PSFS); PGQ, Pelvic Girdle Questionnaire EQ 5D, EuroQol 5 Dimensions; EQ VAS, EuroQol vertical visual analog scale; EDPS, Edinburgh Postnatal Depression Scale; CSQ-CAT, Coping Strategies Questionnaire subscale "Catastrophizing"

For peer review only

Within group comparisons

For ODI or the level of PA, no significant change from baseline to follow-up was detected for any group. Both groups experienced a significant decrease in work ability at follow up. The acupuncture group showed a significant improvement in functioning, measured by PSFS, mean change 0.82 ($p=0.048$) (Table 3, 4).

Both groups reported significantly reduced evening pain intensity (acupuncture -0.96, $p=0.049$ and TENS -1.29, $p=0.003$) and concerns about pain (acupuncture -1.44, $p=0.0012$ and TENS -1.99, $p<0.001$) at follow-up. (Table 3, 4).

There were only minor differences between the ITT and FAS and this did not affect the results except for ODI and CSQ-CAT. For ODI the TENS group showed a significant increase of disability in the FAS but this was not the case in the ITT analysis. Regarding CSQ-CAT the acupuncture group, according to the FAS, reported a significant decrease in catastrophizing beliefs, this was not significant in the ITT.

Discussion

The results of the present study did not show any clinically important differences between the interventions regarding the primary outcome variables.

The measurements of disability (ODI) and daily function (PGQ) showed that PGP resulted in a moderate disability and affected the women's everyday life in various ways. Disability due to PGP/LBP tends to increase over time in pregnancy, [14] but in the present study no difference at follow up was detected. By PSFS, the functional status was assessed in an individually tailored way as the women themselves chose the measured activities. The reported activities covered for example housework, childcare, transportation, specific working tasks and leisure activities. Both groups experienced a significant decrease of work ability (Table 3, 4). As previously shown, work ability is affected by a variety of factors in addition

For peer review only - http://bmjopen.bmj.com/site/about/guidelines.xhtml

For peer review only

to PGP as pregnancy proceeds such as work conditions, general health, bodily changes and tiredness.[48] This result indicates that pregnancy itself has an impact on women's work ability.[14]

At follow-up, the level of PA was maintained for both groups (Table 3, 4). This differs from previous studies that showed that the level of activity decreases as pregnancy proceeds.[11, 49-52] The interventions did reduce evening pain and concern about pain and this reduction may help women to maintain their PA level. The amount of evening pain relief in the present study could be considered small; mean change -0.96 for acupuncture and -1.29 for TENS (Table 3, 4) but a minimal clinical important difference (MCID) of -1.3 is reported as meaningful for pregnant women with PGP.[53] No clinically important difference could be detected between or within groups regarding health. Previous studies show that pregnant women, especially women with PGP and/or LBP, experience lower health compared to non-pregnant women [54] and also that quality of life decreases as pregnancy proceeds.[55] The results of the present study may indicate that a decrease in pain intensity and concern about pain could result in maintenance of overall functioning in daily activities, PA and, consequently, better health.

The women experienced positive effect of the treatments, acupuncture 82.5 % and TENS 60%. This difference could be explained by that the participants in the acupuncture group had more contact with the physiotherapist. The interaction between practitioner and patient may affect the outcome of the intervention [56] and we cannot clearly say that the two interventions alone caused the positive effects experienced by the participants. In this study, both interventions gave significant pain relief but no significant difference between the groups was detected (Table 3, 4), which indicates that the treatment can be chosen based on the individual woman's needs and preferences.

For peer review only - http://bmjopen.bmj.com/site/about/guidelines.xhtml

Women with PGP experience a loss of independence as the pain makes them rely on other people for help, for example with child care and household tasks.[24] Treatment with TENS gives a woman control over her pain relief; she can use it where or when she wants. By contrast, for acupuncture, she must make appointments and go to a clinic for treatment. Some women may need more support and may benefit from regular visits to a physiotherapy clinic whereas others feel confident to perform self-treatment after receiving instructions and information about their complaints. The present study indicates that treatment of PGP during pregnancy can make women feel less concern about their pain. This may lead to less distress and better ability to cope with and manage the pain. Women experiencing higher levels of anxiety/depressive symptoms reported higher levels of disability compared to women with lower levels [19] and anxiety predicted lower levels of PA during pregnancy.[57] These are factors important to identify and manage to prevent the development of longstanding PGP that occur in 10% of women who experienced PGP during pregnancy.[8, 9]

Strengths and limitations

Strengths of the present study are that the interventions were evaluated in both the second and the third trimester and that PGP was clinically verified in all participants. Another strength is the compliance among those who completed the interventions. In the acupuncture group, 38 out of 41 women (93 %) had the assigned ten sessions (mean 9.9) and the women in the TENS group used the treatment at home for mean 26 out of 35 possible days during the treatment period. Only a few minor side effects was reported (Fig. 2), mostly at the beginning of the intervention period and none of them made the women discontinue treatment. Both TENS and acupuncture are according to previous studies safe to use during pregnancy. [25, 27]

It is possible that the design of the present study, where the women had to travel to a specific physiotherapy clinic for testing, acupuncture, and follow-up, made women with more severe

For peer review only

For peer review only - http://bmjopen.bmj.com/site/about/guidelines.xhtml

complaints decline to participate.[58] Another limitation of the present study is the self-reported use of TENS. Since the treatment was not monitored, it is possible that the women applying TENS treatment at home did not follow the instructions even though they followed protocol by registering the sessions. Nevertheless, they experienced positive effects of the treatment.

Conclusion

No differences regarding the effect of acupuncture or TENS as treatment for PGP in pregnancy were detected in the present study. However, both interventions resulted in maintained PA level as well as reduced evening pain and concern about pain. This study suggests that, based on individual women's preferences and needs, either intervention could be recommended as a non-pharmacological alternative for pain relief in order to maintain functioning and PA level and consequently, maintain health as pregnancy proceeds.

Acknowledgements

The authors thank the participating pregnant women, the midwives for their help in recruiting the participants, the physiotherapists that provided the treatments and Nils-Gunnar Pehrsson for statistical advice.

Author Contributions

Concept/idea/research design: A. Gutke, M. Fagevik Olsén, T. Jendman

Writing: A. Svahn Ekdahl, A.Gutke, M. Fagevik Olsén

Data collection: A. Svahn Ekdahl, T. Jendman

Data analysis: A. Svahn Ekdahl, A. Gutke, M. Fagevik Olsén

Project management: A. Gutke, M. Fagevik Olsén, A. Svahn Ekdahl

Consultation (including review of manuscript before submitting): A. Gutke, M. Fagevik Olsén, T. Jendman

For peer review only - http://bmjopen.bmj.com/site/about/guidelines.xhtml

Competing interests

None declared

Funding

This study was financed by Region Västra Götaland, Sweden No. VGFOUREG-310521. The sponsor of the study did not have any influence on study design, collection and analysis/interpretation of data or writing the manuscript.

Trial registration

The trial was registered in "FoU i Sverige" (R&D in Sweden) May 2013, No. 127261 and enrollment of participants started in 2014.

For peer review only

For peer review only - http://bmjopen.bmj.com/site/about/guidelines.xhtml

References

1. Vermani E, Mittal R, Weeks A. Pelvic girdle pain and low back pain in pregnancy: a review. *Pain practice : the official journal of World Institute of Pain* 2010;10(1):60-71. DOI: 10.1111/j.1533-2500.2009.00327.x.
2. Wu WH, Meijer OG, Uegaki K, et al. Pregnancy-related pelvic girdle pain (PPP), I: Terminology, clinical presentation, and prevalence. *Eur Spine J* 2004;13(7):575-89. DOI: 10.1007/s00586-003-0615-y.
3. Gutke A, Boissonnault J, Brook G, et al. The Severity and Impact of Pelvic Girdle Pain and Low-Back Pain in Pregnancy: A Multinational Study. *Journal of women's health (2002)* 2018;27(4):510-7. DOI: 10.1089/jwh.2017.6342.
4. Vleeming A, Albert HB, Ostgaard HC, et al. European guidelines for the diagnosis and treatment of pelvic girdle pain. *Eur Spine J* 2008;17(6):794-819. DOI: 10.1007/s00586-008-0602-4.
5. Dionne CE, Dunn KM, Croft PR, et al. A consensus approach toward the standardization of back pain definitions for use in prevalence studies. *Spine (Phila Pa 1976)* 2008;33(1):95-103. DOI: 10.1097/BRS.0b013e31815e7f94.
6. Aldabe D, Milosavljevic S, Bussey MD. Is pregnancy related pelvic girdle pain associated with altered kinematic, kinetic and motor control of the pelvis? A systematic review. *Eur Spine J* 2012;21(9):1777-87. DOI: 10.1007/s00586-012-2401-1.
7. Noren L, Ostgaard S, Johansson G, et al. Lumbar back and posterior pelvic pain during pregnancy: a 3-year follow-up. *Eur Spine J* 2002;11(3):267-71. DOI: 10.1007/s00586-001-0357-7.
8. Elden H, Gutke A, Kjellby-Wendt G, et al. Predictors and consequences of long-term pregnancy-related pelvic girdle pain: a longitudinal follow-up study. *BMC Musculoskelet Disord* 2016;17:276. DOI: 10.1186/s12891-016-1154-0.
9. Bergstrom C, Persson M, Nergard KA, et al. Prevalence and predictors of persistent pelvic girdle pain 12 years postpartum. *BMC Musculoskelet Disord* 2017;18(1):399. DOI: 10.1186/s12891-017-1760-5.
10. World Health Organisation (WHO). Global recommendations on physical activity for health. Switzerland: World Health Organisation (WHO); 2010 [cited 2020 Jul 02]. Available from: http://www.who.int/dietphysicalactivity/publications/9789241599979/en/.
11. Gjestland K, Bo K, Owe KM, et al. Do pregnant women follow exercise guidelines? Prevalence data among 3482 women, and prediction of low-back pain, pelvic girdle pain and depression. *Br J Sports Med* 2013;47(8):515-20. DOI: 10.1136/bjsports-2012-091344.
12. Lardon E, St-Laurent A, Babineau V, et al. Lumbopelvic pain, anxiety, physical activity and mode of conception: a prospective cohort study of pregnant women. *BMJ open* 2018;8(11):e022508. DOI: 10.1136/bmjopen-2018-022508.
13. Sydsjo A, Alexanderson K, Dastserri M, et al. Gender differences in sick leave related to back pain diagnoses: influence of pregnancy. *Spine (Phila Pa 1976)* 2003;28(4):385-9. DOI: 10.1097/01.brs.0000048467.77507.fc.
14. Gutke A, Olsson CB, Vollestad N, et al. Association between lumbopelvic pain, disability and sick leave during pregnancy - a comparison of three Scandinavian cohorts. *J Rehabil Med* 2014;46(5):468-74. DOI: 10.2340/16501977-1801.
15. Backhausen M, Damm P, Bendix J, et al. The prevalence of sick leave: Reasons and associated predictors - A survey among employed pregnant women. *Sexual & reproductive healthcare : official journal of the Swedish Association of Midwives* 2018;15:54-61. DOI: 10.1016/j.srhc.2017.11.005.
16. Hansen ML, Thulstrup AM, Juhl M, et al. Predictors of sickness absence in pregnancy: a Danish cohort study. *Scand J Work Environ Health* 2015;41(2):184-93. DOI: 10.5271/sjweh.3470.

For peer review only - http://bmjopen.bmj.com/site/about/guidelines.xhtml

17. Shiri R, Coggon D, Falah-Hassani K. Exercise for the prevention of low back and pelvic girdle pain in pregnancy: A meta-analysis of randomized controlled trials. *European Journal of Pain (United Kingdom)* 2018;22(1):19-27. DOI: 10.1002/ejp.1096.
18. Gutke A, Josefsson A, Oberg B. Pelvic girdle pain and lumbar pain in relation to postpartum depressive symptoms. *Spine (Phila Pa 1976)* 2007;32(13):1430-6. DOI: 10.1097/BRS.0b013e318060a673.
19. Virgara R, Maher C, Van Kessel G. The comorbidity of low back pelvic pain and risk of depression and anxiety in pregnancy in primiparous women. *BMC Pregnancy Childbirth* 2018;18(1):288. DOI: 10.1186/s12884-018-1929-4.
20. Davenport MH, McCurdy AP, Mottola MF, et al. Impact of prenatal exercise on both prenatal and postnatal anxiety and depressive symptoms: a systematic review and meta-analysis. *Br J Sports Med* 2018;52(21):1376-85. DOI: 10.1136/bjsports-2018-099697.
21. Davenport MH, Ruchat SM, Poitras VJ, et al. Prenatal exercise for the prevention of gestational diabetes mellitus and hypertensive disorders of pregnancy: a systematic review and meta-analysis. *Br J Sports Med* 2018;52(21):1367-75. DOI: 10.1136/bjsports-2018-099355.
22. Lupattelli A, Spigset O, Twigg MJ, et al. Medication use in pregnancy: a cross-sectional, multinational web-based study. *BMJ open* 2014;4(2):e004365. DOI: 10.1136/bmjopen-2013-004365.
23. Mitchell AA, Gilboa SM, Werler MM, et al. Medication use during pregnancy, with particular focus on prescription drugs: 1976-2008. *Am J Obstet Gynecol* 2011;205(1):51.e1-8. DOI: 10.1016/j.ajog.2011.02.029.
24. Mackenzie J, Murray E, Lusher J. Women's experiences of pregnancy related pelvic girdle pain: A systematic review. *Midwifery* 2018;56:102-11. DOI: 10.1016/j.midw.2017.10.011.
25. Liddle SD, Pennick V. Interventions for preventing and treating low-back and pelvic pain during pregnancy. *The Cochrane database of systematic reviews* 2015(9):Cd001139. DOI: 10.1002/14651858.CD001139.pub4.
26. Gutke A, Betten C, Degerskar K, et al. Treatments for pregnancy-related lumbopelvic pain: a systematic review of physiotherapy modalities. *Acta Obstet Gynecol Scand* 2015;94(11):1156-67. DOI: 10.1111/aogs.12681.
27. Keskin EA, Onur O, Keskin HL, et al. Transcutaneous electrical nerve stimulation improves low back pain during pregnancy. *Gynecol Obstet Invest* 2012;74(1):76-83. DOI: 10.1159/000337720.
28. Johnson M. Transcutaneous Electrical Nerve Stimulation: Mechanisms, Clinical Application and Evidence. *Reviews in pain* 2007;1(1):7-11. DOI: 10.1177/204946370700100103.
29. Lund I, Lundeberg T. Mechanisms of acupuncture. 2016;4(4):26-30. DOI: 10.1016/j.arthe.2016.12.001.
30. Shah S, Banh ET, Koury K, et al. Pain Management in Pregnancy: Multimodal Approaches. *Pain research and treatment* 2015;2015:987483. DOI: 10.1155/2015/987483.
31. Carter RE, Lubinsky J. Rehabilitation Research: Principles and Applications. 5th ed. St Louis, MO: Elsevier; 2016. p. 110-1.
32. Gutke A, Kjellby-Wendt G, Oberg B. The inter-rater reliability of a standardised classification system for pregnancy-related lumbopelvic pain. *Man Ther* 2010;15(1):13-8. DOI: 10.1016/j.math.2009.05.005.
33. Springer BA, Marin R, Cyhan T, et al. Normative values for the unipedal stance test with eyes open and closed. *J Geriatr Phys Ther* 2007;30(1):8-15. DOI: 10.1519/00139143-200704000-00003.
34. Elden H, Ladfors L, Olsen MF, et al. Effects of acupuncture and stabilising exercises as adjunct to standard treatment in pregnant women with pelvic girdle pain: randomised single blind controlled trial. *BMJ* 2005;330(7494):761. DOI: 10.1136/bmj.38397.507014.E0.
35. Fairbank JC, Pynsent PB. The Oswestry Disability Index. *Spine (Phila Pa 1976)* 2000;25(22):2940-52; discussion 52. DOI: 10.1097/00007632-200011150-00017.

For peer review only - http://bmjopen.bmj.com/site/about/guidelines.xhtml

36. Westaway MD, Stratford PW, Binkley JM. The patient-specific functional scale: validation of its use in persons with neck dysfunction. *J Orthop Sports Phys Ther* 1998;27(5):331-8. DOI: 10.2519/jospt.1998.27.5.331.
37. de Zwart BC, Frings-Dresen MH, van Duivenbooden JC. Test-retest reliability of the Work Ability Index questionnaire. *Occup Med (Lond)* 2002;52(4):177-81. DOI: 10.1093/occmed/52.4.177.
38. Haakstad LA, Voldner N, Henriksen T, et al. Physical activity level and weight gain in a cohort of pregnant Norwegian women. *Acta Obstet Gynecol Scand* 2007;86(5):559-64. DOI: 10.1080/00016340601185301.
39. Stuge B, Garratt A, Krogstad Jenssen H, et al. The pelvic girdle questionnaire: a condition-specific instrument for assessing activity limitations and symptoms in people with pelvic girdle pain. *Phys Ther* 2011;91(7):1096-108. DOI: 10.2522/ptj.20100357.
40. Gutke A, Stuge B, Elden H, et al. The Swedish version of the pelvic girdle questionnaire, cross-cultural adaptation and validation. *Disabil Rehabil* 2019:1-8. DOI: 10.1080/09638288.2018.1515991.
41. Hjermstad MJ, Fayers PM, Haugen DF, et al. Studies comparing Numerical Rating Scales, Verbal Rating Scales, and Visual Analogue Scales for assessment of pain intensity in adults: a systematic literature review. *J Pain Symptom Manage* 2011;41(6):1073-93. DOI: 10.1016/j.jpainsymman.2010.08.016.
42. Elkins G, Staniunas R, Rajab MH, et al. Use of a numeric visual analog anxiety scale among patients undergoing colorectal surgery. *Clin Nurs Res* 2004;13(3):237-44. DOI: 10.1177/1054773803262222.
43. Rabin R, de Charro F. EQ-5D: a measure of health status from the EuroQol Group. *Ann Med* 2001;33(5):337-43. DOI: 10.3109/07853890109002087.
44. Kozinszky Z, Dudas RB. Validation studies of the Edinburgh Postnatal Depression Scale for the antenatal period. *J Affect Disord* 2015;176:95-105. DOI: 10.1016/j.jad.2015.01.044.
45. Rubertsson C, Borjesson K, Berglund A, et al. The Swedish validation of Edinburgh Postnatal Depression Scale (EPDS) during pregnancy. *Nordic journal of psychiatry* 2011;65(6):414-8. DOI: 10.3109/08039488.2011.590606.
46. Rosenstiel AK, Keefe FJ. The use of coping strategies in chronic low back pain patients: Relationship to patient characteristics and current adjustment. *Pain* 1983;17(1):33-44. DOI: http://dx.doi.org/10.1016/0304-3959(83)90125-2.
47. Jensen I, Linton S. Coping Strategies Questionnaire (CSQ): Reliability of the Swedish version of the CSQ. *Scandinavian Journal of Behaviour Therapy* 1993;22:139-45. DOI: 10.1080/16506079309455940.
48. Truong BT, Lupattelli A, Kristensen P, et al. Sick leave and medication use in pregnancy: A European web-based study. *BMJ open* 2017;7(8). DOI: 10.1136/bmjopen-2016-014934.
49. Gaston A, Vamos CA. Leisure-time physical activity patterns and correlates among pregnant women in Ontario, Canada. *Maternal and child health journal* 2013;17(3):477-84. DOI: 10.1007/s10995-012-1021-z.
50. Walsh JM, McGowan C, Byrne J, et al. Prevalence of physical activity among healthy pregnant women in Ireland. *Int J Gynaecol Obstet* 2011;114(2):154-5. DOI: 10.1016/j.ijgo.2011.02.016.
51. Evenson KR, Savitz DA, Huston SL. Leisure-time physical activity among pregnant women in the US. *Paediatr Perinat Epidemiol* 2004;18(6):400-7. DOI: 10.1111/j.1365-3016.2004.00595.x.
52. Hegaard HK, Damm P, Hedegaard M, et al. Sports and leisure time physical activity during pregnancy in nulliparous women. *Maternal and child health journal* 2011;15(6):806-13. DOI: 10.1007/s10995-010-0647-y.
53. Ogollah R, Bishop A, Lewis M, et al. Responsiveness and Minimal Important Change for Pain and Disability Outcome Measures in Pregnancy-Related Low Back and Pelvic Girdle Pain. *Phys Ther* 2019. DOI: 10.1093/ptj/pzz107.

For peer review only - http://bmjopen.bmj.com/site/about/guidelines.xhtml

54. Olsson C, Nilsson-Wikmar L. Health-related quality of life and physical ability among pregnant women with and without back pain in late pregnancy. *Acta Obstet Gynecol Scand* 2004;83(4):351-7. DOI: 10.1111/j.0001-6349.2004.00384.x.

55. Sahrakorpi N, Koivusalo SB, Stach-Lempinen B, et al. "The Burden of Pregnancy"; heavier for the heaviest? The changes in Health Related Quality of Life (HRQoL) assessed by the 15D instrument during pregnancy and postpartum in different body mass index groups: a longitudinal survey. *Acta Obstet Gynecol Scand* 2017;96(3):352-8. DOI: 10.1111/aogs.13068.

56. Blasini M, Peiris N, Wright T, et al. The Role of Patient-Practitioner Relationships in Placebo and Nocebo Phenomena. *Int Rev Neurobiol* 2018;139:211-31. DOI: 10.1016/bs.irn.2018.07.033.

57. St-Laurent A, Lardon E, Babineau V, et al. Reproductive history, maternal anxiety and past physical activity practice predict physical activity levels throughout pregnancy. *Prev Med Rep* 2019;16:100992. DOI: 10.1016/j.pmedr.2019.100992.

58. Morino S, Ishihara M, Umezaki F, et al. Low back pain and causative movements in pregnancy: a prospective cohort study. *BMC Musculoskelet Disord* 2017;18(1):416. DOI: 10.1186/s12891-017-1776-x.

For peer review only

Figure legends.

Figure 1. Flowchart of the study according to the CONSORT requirements

Figure 2. Reported side effects of the interventions, Acupuncture (n=9/41) and TENS (n=6/45)

For peer review only

For peer review only - http://bmjopen.bmj.com/site/about/guidelines.xhtml

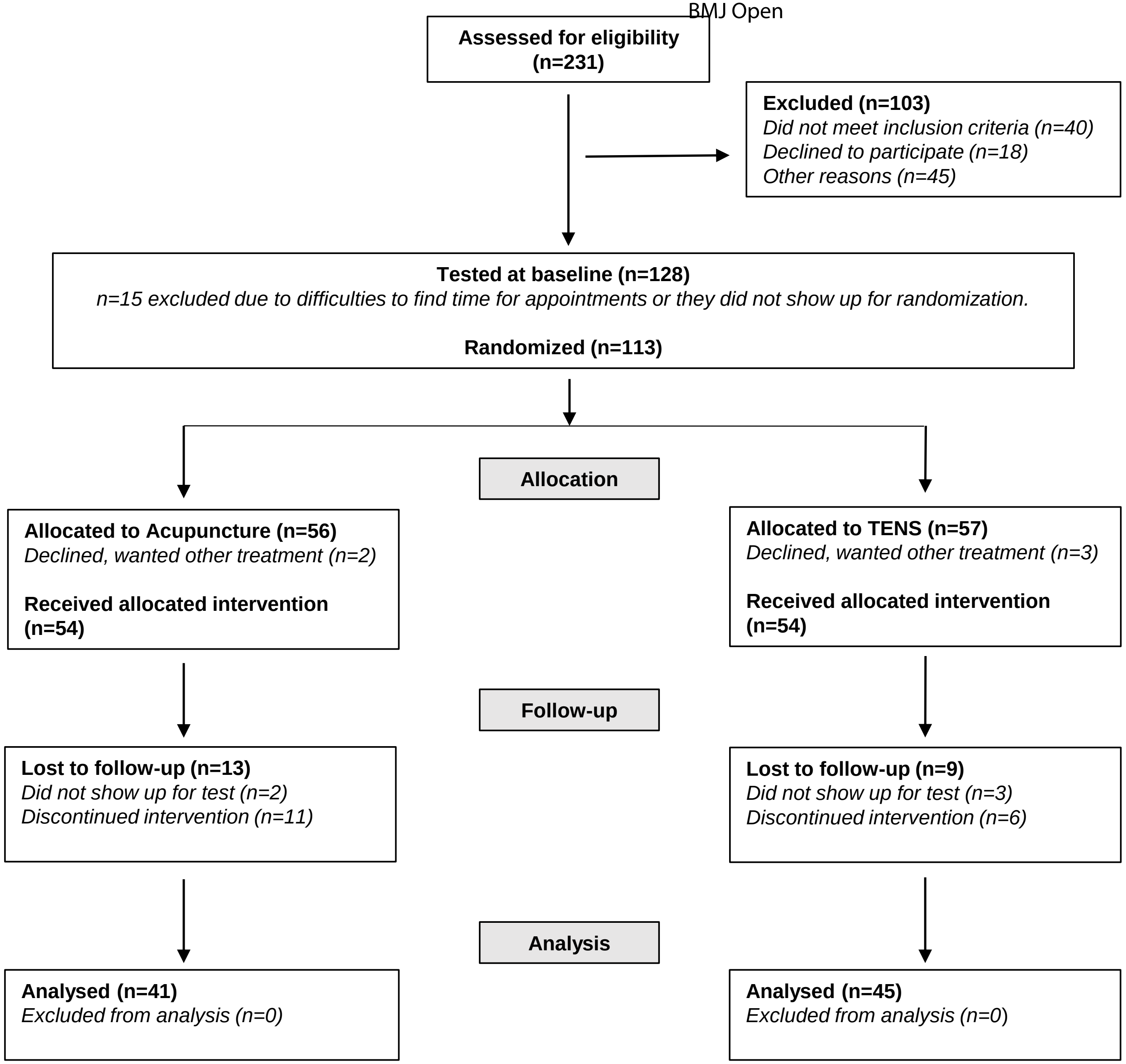
Assessed for eligibility (n=231)
Excluded (n=103)
Did not meet inclusion criteria (n=40)
Declined to participate (n=18)
Other reasons (n=45)
Tested at baseline (n=128)
n=15 excluded due to difficulties to find time for appointments or they did not show up for randomization.
Randomized (n=113)
Allocation
Allocated to Acupuncture (n=56)
Declined, wanted other treatment (n=2)
Received allocated intervention (n=54)
Allocated to TENS (n=57)
Declined, wanted other treatment (n=3)
Received allocated intervention (n=54)
Follow-up
Lost to follow-up (n=13)
Did not show up for test (n=2)
Discontinued intervention (n=11)
Lost to follow-up (n=9)
Did not show up for test (n=3)
Discontinued intervention (n=6)
Analysis
Analysed (n=41)
Excluded from analysis (n=0)
Analysed (n=45)
Excluded from analysis (n=0)

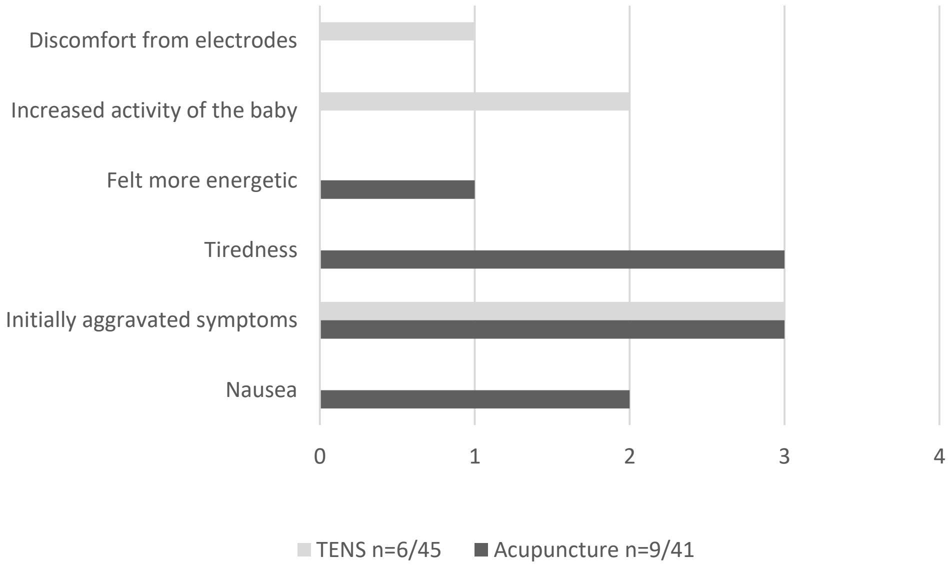
Discomfort from electrodes
Increased activity of the baby
Felt more energetic
Tiredness
Initially aggravated symptoms
Nausea
0
1
2
3
4
TENS n=6/45
Acupuncture n=9/41

BMJ Open

Maintenance of physical activity level, functioning and health after non-pharmacological treatment of pelvic girdle pain with either transcutaneous electric nerve stimulation or acupuncture. A randomized controlled trial

Journal:	*BMJ Open*
Manuscript ID	bmjopen-2020-046314.R1
Article Type:	Original research
Date Submitted by the Author:	26-May-2021
Complete List of Authors:	Svahn Ekdahl, Annika; Sahlgrenska Academy, Department of Health and Rehabilitation; Physiotherapy<br>Olsen, Monika Fagevik; Sahlgrenska universitetssjukhuset, Physiotherapy; Sahlgrenska Academy, Department of Health and Rehabilitation; Physiotherapy<br>Jendman, Tove; Physiotherapy Clinic "I Rorelse"<br>Gutke, Annelie; Sahlgrenska Academy, Department of Health and Rehabilitation; Physiotherapy
**Primary Subject Heading**:	Obstetrics and gynaecology
Secondary Subject Heading:	General practice / Family practice
Keywords:	PAIN MANAGEMENT, Maternal medicine < OBSTETRICS, Back pain < ORTHOPAEDIC & TRAUMA SURGERY, Musculoskeletal disorders < ORTHOPAEDIC & TRAUMA SURGERY

SCHOLARONE™
Manuscripts

I, the Submitting Author has the right to grant and does grant on behalf of all authors of the Work (as defined in the below author licence), an exclusive licence and/or a non-exclusive licence for contributions from authors who are: i) UK Crown employees; ii) where BMJ has agreed a CC-BY licence shall apply, and/or iii) in accordance with the terms applicable for US Federal Government officers or employees acting as part of their official duties; on a worldwide, perpetual, irrevocable, royalty-free basis to BMJ Publishing Group Ltd ("BMJ") its licensees and where the relevant Journal is co-owned by BMJ to the co-owners of the Journal, to publish the Work in this journal and any other BMJ products and to exploit all rights, as set out in our licence.

The Submitting Author accepts and understands that any supply made under these terms is made by BMJ to the Submitting Author unless you are acting as an employee on behalf of your employer or a postgraduate student of an affiliated institution which is paying any applicable article publishing charge ("APC") for Open Access articles. Where the Submitting Author wishes to make the Work available on an Open Access basis (and intends to pay the relevant APC), the terms of reuse of such Open Access shall be governed by a Creative Commons licence – details of these licences and which Creative Commons licence will apply to this Work are set out in our licence referred to above.

Other than as permitted in any relevant BMJ Author's Self Archiving Policies, I confirm this Work has not been accepted for publication elsewhere, is not being considered for publication elsewhere and does not duplicate material already published. I confirm all authors consent to publication of this Work and authorise the granting of this licence.

For peer review only

Maintenance of physical activity level, functioning and health after non-pharmacological treatment of pelvic girdle pain with either transcutaneous electric nerve stimulation or acupuncture. A randomized controlled trial

Annika Svahn Ekdahl[1], Monika Fagevik Olsén[1,2,] Tove Jendman[3], Annelie Gutke[1]

[1] Institute of Neuroscience and Physiology, Section of Health and Rehabilitation, Physiotherapy, Sahlgrenska Academy, University of Gothenburg, Gothenburg, Sweden

[2] Department of Physiotherapy, Sahlgrenska University Hospital, Gothenburg, Sweden

[3] Physiotherapy clinic "I Rörelse," Gothenburg, Sweden

Correspondence to:
Annika Svahn Ekdahl
University of Gothenburg
Institute of Neuroscience and Physiology
Department of Physiotherapy
Box 455
405 30 Gothenburg
Sweden
Phone: +46 31 786 5724 Mobile: +46 766 185724
E-mail: annika.svahn-ekdahl@neuro.gu.se

Word count; Abstract: 299, Body of manuscript: 3599

For peer review only

For peer review only - http://bmjopen.bmj.com/site/about/guidelines.xhtml

Abstract

Objective

To investigate if there are differences between acupuncture and transcutaneous electric nerve stimulation (TENS) as treatment for pelvic girdle pain (PGP) in pregnancy in order to manage pain and thus maintain health and functioning in daily activities and physical activity (PA).

Design

Randomized controlled trial

Setting and participants

Pregnant women (n=113) with clinically verified PGP in gestational weeks 12–28, recruited from maternity healthcare centers, randomized (1:1) into two groups. Exclusion criteria: Any obstetric complication, systemic disease or previous disorder that could contradict tests or treatment.

Interventions

The intervention consisted of either ten acupuncture sessions (two sessions per week) provided by a physiotherapist or daily home-based TENS during five weeks.

Primary outcome variables

Disability (Oswestry Disability Index), functioning (Patient Specific Functional Scale), work ability (Work Ability Index) and PA-level according to general recommendations.

Secondary outcome variables

Functioning related to PGP (Pelvic Girdle Questionnaire), evening pain intensity (Numeric Rating Scale, NRS), concern about pain (NRS), health (EuroQoL 5D), symptoms of

For peer review only

depression/catastrophizing (Edinburgh Postnatal Depression Scale/Coping Strategies Questionnaire).

Results

No mean differences were detected between the groups. Both groups managed to preserve their functioning and PA level at follow up. This may be due to significantly ($p<0.05$) reduced within groups evening pain intensity; acupuncture -0.96 (95% CI -1.91 to -0.01; p=0.049), TENS -1.29 (95% CI -2.13 to -0.44; p=0.003) and concern about pain; acupuncture -1.44 (95% CI -2.31 to -0.57; p=0.0012), TENS -1.99 (95% CI -2.81 to -1.17; $p<0.0001$). The acupuncture group showed an improvement in functioning at follow up; 0.82 (95% CI 0.01 to 1.63; p=0.048)

Conclusion

Treating PGP with acupuncture or TENS resulted in maintenance of functioning and physical activity and also less pain and concern about pain. Either intervention could be recommended as a non-pharmacological alternative for pain relief and may enable pregnant women to stay active.

Trial registration: In “FoU i Sverige” (R&D in Sweden) No. 12726.
https://www.researchweb.org/is/sverige/project/127261

Article Summary

Strengths and limitations of this study

- All participants had clinically verified PGP during pregnancy.
- Interventions were evaluated both in the 2nd and 3rd trimester of pregnancy, with good adherence in both intervention groups.

For peer review only

69 • Lower self-reported satisfaction in the TENS group could be a result of less contact with the treating physiotherapist than in the acupuncture group.
71 • It is possible that the participants did not use TENS according to instructions, as this was self-reported.
73 • The study design, where pregnant women had to travel to the clinic for assessment and/or intervention, may have affected the ability to recruit women with more severe PGP to the study.

For peer review only

Introduction

Pelvic girdle pain (PGP) and/or low back pain (LBP) is reported internationally by over 50% of pregnant women.[1-3] PGP has musculoskeletal origin and is localized to the area between the posterior iliac crest and the gluteal fold, most commonly around the sacroiliac joints and/or pubic bone, sometimes with radiating pain in the thighs.[4] Limitations in daily functioning and walking difficulties, especially a feeling of "catching" of the leg are common symptoms.[2] These symptoms differ from those associated with LBP, which is defined as pain in the area between the lower ribs and the iliac crest, with or without radiating pain to the legs, and is often described as worse when sitting.[5]

The etiology of PGP is multifactorial. Increased pregnancy-related ligamentous laxity may result in altered mechanics in the pelvic joints.[6] Pain may occur if these alterations cannot be compensated for by neuromuscular control.[4] PGP most commonly occurs around gestation week 18, but can occur at any time during pregnancy. Among women who experience PGP during pregnancy, about 20% suffer from persistent pain 4 months postpartum.[7] Previous long-term studies have reported that approximately 10% suffer from remaining PGP more than 11 years postpartum.[8,9] Experiencing PGP during pregnancy is a risk factor for having PGP also in a next pregnancy.[2]

Pregnant women are advised to follow the general recommendations for physical activity (PA), of at least 150 minutes of moderate intensity or at least 75 minutes of high intensity PA per week or a combination of both.[10] Few pregnant women reach this level of PA and a common cause is PGP.[11] A high level of pain during pregnancy results in more severe limitations in daily life and less PA.[12]

There is a higher rate of sick leave among pregnant women compared to age-matched, non-pregnant women and a major reason is PGP and/or LBP.[13-15] Studies show a reduced risk for

For peer review only

For peer review only - http://bmjopen.bmj.com/site/about/guidelines.xhtml

sick leave if the woman can continue to stay active and exercise throughout her pregnancy.[16,17] Women with PGP and/or LBP have an increased risk of prenatal anxiety, depressive symptoms and postpartum depression compared to healthy pregnant women.[18,19] If pregnant women maintains the recommended levels of PA, both the odds of getting prenatal depression and the severity of it is reduced.[20] Furthermore, there is a risk in pregnancy for complications such as hypertension, diabetes and preeclampsia and this risk is reduced if the woman stays active.[21]

The use of medication in pregnancy has increased over the years [22,23] with a risk for overuse of analgesics among women having PGP.[24] Therefore, it is important to provide efficient treatment alternatives. The best evidence non-pharmacological treatments for PGP are acupuncture, a stabilizing pelvic belt, and physical exercise.[25,26] There is, however, limited evidence for the use of transcutaneous electrical nerve stimulation (TENS),[25-27] even if it is widely used in physiotherapy clinical practice. The physiological mechanisms of acupuncture and TENS are similar. Both peripheral and central mechanisms of pain control are stimulated, as per the gate control theory, activating descending pathways for pain control and release of endogenous opioids.[28,29] No specific adverse effects regarding use in pregnancy are reported for any of the interventions mentioned here.[30]

The aim of this study was to investigate if there are differences between acupuncture and TENS as treatments for PGP in order to manage pain and thus maintain health and functioning in daily activities and PA.

Methods

A randomized, controlled design was used to compare acupuncture and TENS as pain relief for PGP in pregnancy. The decision to randomize into two different groups was made based

For peer review only

For peer review only - http://bmjopen.bmj.com/site/about/guidelines.xhtml

on ethical considerations. It is not ethical to withhold evidence based treatment, therefore the experimental treatment (TENS) was given to one group and acupuncture, which is known to be effective for PGP [25,26], to an active control group.[31] The study was approved by the regional ethical review board in Gothenburg, Sweden (No. 308-13).

Setting and Participants

The data collection took place 2014–2018 in two Swedish cities. Two independent physiotherapists located in each city conducted tests and treatment. Women with PGP were briefly informed about the study at Maternity Health Care Centers and those who were interested were contacted by the independent test leader for detailed information and for initial screening for inclusion.

Inclusion criteria

- Single fetus pregnancy
- Pregnancy-related PGP or combined PGP/LBP in gestational weeks 12–28
- Pain located distally/laterally of L5/S1, over the buttocks, and/or the pubic bone, verified by ≥2 positive pain provocation tests or a positive active straight leg raise (ASLR)
- A score of ≥20% on the Oswestry Disability Index (ODI) and/or ≤6 in one self-chosen activity of the Patient-Specific Functional Scale (PSFS)
- Ability to read and understand Swedish

Exclusion criteria

- Previous fracture, surgery, or malignant disease in the back, pelvis, or hips
- Any systemic disease or obstetric complication that contraindicates treatment or tests
- Contraindications for TENS; pacemaker; decreased sensation in the treatment area
- Contraindications for acupuncture; treatment with anticoagulants

For peer review only - http://bmjopen.bmj.com/site/about/guidelines.xhtml

- Start of other treatment during the study period

Those eligible for participation underwent a clinical examination to verify PGP and answered the baseline questionnaire. All participants gave oral and written consent. The clinical examination consisted of a reliable procedure, which included a neurological examination, hip rotation range of motion test, pelvic pain provocation tests, a brief mechanical assessment of the lumbar spine and the ASLR test.[32] The examination also included a single-leg balance test.[33]

Randomization and Interventions

Before start of intervention, the participants each drew a sealed, opaque envelope, prepared from a computer-generated list by an independent research assistant, for assignment to either the acupuncture or TENS group (1:1).

The intervention lasted for 5 weeks in both groups. All women got written general advice on how to manage PGP in addition to the assigned treatment.

Acupuncture

The intervention consisted of 10 acupuncture sessions (two sessions per week), provided by an experienced physiotherapist, trained in Western medical acupuncture. It was decided, due to ethical considerations, not to continue the intervention for any woman that experienced sufficient pain relief in fewer than ten sessions. The treating physiotherapist chose the acupuncture points, based on the individual woman's symptoms and clinical presentation, from a protocol published in a previous study where point localization and insertion depth are described in detail.[34] Disposable, stainless steel needles (China Classic; 0.25x15 mm, 0.30x30 mm, 0.30x50 mm) were used. After insertion and manual stimulation to evoke the sensation of *de Qi* (a radiating sensation or a feeling of heaviness) from the insertion point, the needles were left in situ for 30 minutes and manually stimulated after 10 and 20 minutes.

For peer review only

For peer review only - http://bmjopen.bmj.com/site/about/guidelines.xhtml

TENS

An experienced physiotherapist tried out the electrode placement according to the individual woman's symptoms and clinical presentation. The women received oral and written instructions regarding their individual electrode placement and then used the TENS device (Cefar Primo PRO; DJO Nordic AB, Malmö, Sweden) at home for at least 30 minutes per day for 5 weeks. High frequency stimulation (80 Hz) was used with an intensity of the stimulation that produced a strong but comfortable sensation in the treated area. The women were instructed to increase the intensity of the stimulation during the treatment session in order to maintain the strong but comfortable sensation. Each woman met the physiotherapist on a follow-up visit after 1 week to discuss treatment and to adjust electrode placement and stimulation mode if needed. In case of absence of effect after the first week of treatment, the stimulation mode could be changed to low frequency (2 Hz) in order to receive optimal pain relief. After 3 weeks, one follow-up telephone call to each woman was made to ensure that the intervention was working well. After 5 weeks the participants made a final visit to the physiotherapist.

Outcomes and Follow-up

Because of the present study's focus on functioning, a broad spectrum of outcome variables was included. All outcomes were measured at baseline and repeated at follow up.

Primary outcomes

- Disability, defined as affected function due to PGP, measured by ODI, range 0–100%, where 0% = no disability.[35]
- Functional status, assessed by the PSFS. Two self-chosen everyday activities rated on a numeric rating scale (NRS) of 0–10; 10 = can perform the activity unrestrictedly or as before the onset of PGP.[36]

For peer review only - http://bmjopen.bmj.com/site/about/guidelines.xhtml

- Work ability, measured by one question from the Work Ability Index (WAI). Current work ability marked on a NRS of 0–10; 10 = work ability at its best.[37]
- Physical activity level, number of days/week with activity for 30 minutes at moderate intensity and/or activity at high intensity for 20 minutes.[38]

Secondary outcomes

- Everyday functioning affected by PGP, measured by the Pelvic Girdle Questionnaire (PGQ). A total score and scores for the subscales of "Activity" and "Symptoms", where 0% = no disability.[39,40]
- Evening PGP intensity at its worst, measured by a NRS of 0–10; 10 = worst pain,[41] and by a pain drawing to illustrate the pain location.
- Concern about PGP, assessed using a NRS of 0–10; 10 = extremely concerned.[42]
- Health, measured by the EuroQol 5-dimension questionnaire (EQ5D), a total score of 1.0 indicates full health. Participants also marked their current health status on the EuroQol vertical visual analog scale (EQ VAS); 100 = best imaginable health state.[43]
- Symptoms of depression, measured by the Edinburgh Postnatal Depression Scale (EPDS), range 0-30. A score >13 indicates symptoms of depression that require further examination.[44,45]
- Catastrophizing beliefs via the subscale "Catastrophizing" (from the Coping Strategies Questionnaire, CSQ-CAT) range 0-36; 0 = no catastrophizing beliefs.[46,47]
- Questions were also asked about use of analgesics, sick leave, height/weight, education level, family income, and possible side effects of the treatment.

After the intervention period the clinical examination was repeated by the independent test-leader, who was blinded to the treatment allocation, and the participants completed a follow-up questionnaire.

Patient and public involvement

For peer review only - http://bmjopen.bmj.com/site/about/guidelines.xhtml

No patients or members of the public was involved in design, conduction or interpretation of the study. Patient satisfaction was measured at follow up.

Statistical analysis

Sample size

With a β-level of 80%, an α-level of 5%, and a difference in functioning between groups of 10%, measured using the ODI, a sample size of at least 30 women/group was required. As the dropout rate for the postpartum follow-up and the planned 3.5 year long-term follow-up was calculated to be 25% and 50%, respectively, the plan was to include 60 women in each group.

Analysis

The analysis was done according to intention to treat (ITT), with the use of multiple imputation with 50 imputation datasets generated followed by one analysis of each of these 50 datasets followed by a pooled analysis of the 50 tests. The imputation of each value was performed by Fully Conditional Specifications. For continuous variables T-test was applied due to central limit theorem and for dichotomous variables change in proportion was applied. A Full Analysis Set (FAS) was also performed, using Fischer's non-parametric permutation test, including only those women who completed the intervention. Change from baseline to follow up is expressed as mean (SD)/ median (min; max)/ 95% CI for mean. For dichotomous variables, the Chi Square-test was used. The level of statistical significance was set to $p<0.05$. The analysis was made using IBM SPSS Statistics v. 24 (IBM Corp., Armonk, NY, USA) and SAS v. 9.4 (SAS Institute, Cary, NC, USA).

Results

Altogether 15 women, who fulfilled the inclusion criteria, were excluded after the baseline test due to difficulties in finding time for treatment or they did not show up for randomization

For peer review only - http://bmjopen.bmj.com/site/about/guidelines.xhtml

(Fig. 1). A total of 113 women were randomized into two groups (Fig. 1). No relevant differences between the intervention groups were found at baseline. At baseline, women in both groups reported pain in their pelvic and/or lumbar region during the last 4 weeks, which affected their daily activities for more than 1 day/week. (Table 1).

Table. 1 Descriptive data

Variable	Acupuncture n=56	TENS n=57	Excluded after baseline-test n=15
**Maternal age, mean (SD), median (min; max)**	30.5 (3.96)<br>30.0 (20.0; 38.0)	31.1 (4.38)<br>32.0 (22.0; 43.0)	31.9 (3.92)<br>32.0 (27.0; 40.0)
**Gestational weeks, mean (SD), median (min; max)**	20.9 (5.32)<br>21.5 (11.0; 28.0)	20.8 (5.18)<br>22.0 (10.0; 28.0)	22.4 (5.17)<br>24.0 (13.0; 28.0)
**BMI, mean (SD), median (min; max)**	25.6 (4.53)<br>25.0 (19.5; 41.8)	26.5 (3.87)<br>26.2 (19.7; 35.0)<br>*n=55*	27.6 (5.83)<br>27.6 (18.2; 41.0)
**Parity, n (%)**			
0	21 (37.5)	22 (38.6)	6 (40.0)
1	30 (53.6)	28 (49.1)	5 (33.3)
≥2	5 (8.9)	7 (12.3)	4 (26.7)
**Education level, n (%)**			
Elementary school	1 (1.8)	2 (3.5)	0
High school	12 (22.4)	20 (35.1)	3 (20.0)
Higher education	43 (76.8)	35 (61.4)	12 (80.0)
**Occupation, n (%)**			
Paid employment	47 (83.9)	47 (82.5)	14 (93.3)
Not in paid employment	3 (5.4)	3 (5.3)	0
Parental leave	0	2 (3.5)	0
Combination	2 (3.6)	3 (5.3)	0
Other	4 (7.1)	2 (3.5)	1 (6.7)
**Sick leave, n (%)**	9 (16.1)	7 (12.3)	3 (20.0)
**Manage financially, n (%)**			
Very well	22 (39.3)	34 (59.6)	7 (46.7)
Quite well	30 (53.6)	21 (36.8)	7 (46.7)
Neither well or badly	4 (7.1)	2 (3.5)	1 (6.7)
Quite badly	0	0	0
Very badly	0	0	0
**LBP/PGP last 4 weeks, n (%)**	56 (100)	57 (100)	15 (100)
**Pain affects daily activities, n (%)**	53 (94.6)	57 (100)	15 (100)
**Pain how often, n (%)**			
Some days	3 (5.4)	7 (12.3)	0
Most days	16 (28.6)	19 (33.3)	8 (53.3)
Every day	37 (66.1)	31 (54.4)	7 (46.7)
**Use of analgesics, n (%)**	6 (10.9)<br>*n=55*	3 (5.3)	0
**ODI, mean (SD), median (min; max)**	34.5 (13.19)<br>34.0 (8.0; 68.0)	30.1 (11.82)<br>30.0 (8.0; 60.0)	32.5 (12.22)<br>32.0 (12.0; 52.0)
**PSFS NRS, mean (SD), median (min; max)**	2.8 (1.46)<br>2.5 (0; 7.0)	3.2 (1.55)<br>3.0 (0; 7.5)	3.9 (2.33)<br>3.0 (0.5; 9.0)
**Work ability NRS, mean (SD), median (min; max)**	6.2 (2.36)<br>7.0 (0; 10.0)<br>*n=55*	6.7 (2.41)<br>7.0 (0; 10.0)<br>*n=56*	5.7 (3.02)<br>6.0 (0; 9.0)

For peer review only

For peer review only - http://bmjopen.bmj.com/site/about/guidelines.xhtml

**Recommended PA; yes, n (%)**	13 (23.2)	14 (24.6)	6 (40)
**PGQ total, mean (SD), median (min; max)**	49.3 (17.70)<br>50.7 (8.0; 90.7)<br>*n=55*	47.0 (12.91)<br>45.6 (22.2; 82.6))<br>*n=56*	53.2 (18.1)<br>60.0 (20.0; 77.3)
**PGQ activity, mean (SD), median (min; max)**	48.1 (17.76)<br>50.0 (6.7; 90.0)<br>*n=55*	46.3 (13.55)<br>45.3 (24.6; 82.5)<br>*n=56*	52.1 (18.63)<br>58.3 (16.7; 76.7)
**PGQ symptom, mean (SD), median (min; max)**	51.5 (17.17)<br>53.3 (13.3; 93.3)<br>*n=55*	49.4 (13.27)<br>46.7 (13.3; 73.3)	57.1 (15.98)<br>60.0 (26.67; 80.0)
**Evening pain intensity NRS, mean (SD), median (min; max)**	6.8 (1.95)<br>7.0 (2.0; 10.0)<br>*n=55*	6.6 (1.56)<br>7.0 (3.0; 9.0)	7.4 (1.56)<br>8.0 (4.0; 9.0)
**Concern about pain NRS, mean (SD), median (min; max)**	4.2 (2.44)<br>5.0 (0; 10.0)	4.6 (2.34)<br>5.0 (0; 10.0)	4.7 (2.69)<br>5.0 (0; 10.0)
**EQ 5D, mean (SD), median (min; max)**	0.55 (0.28)<br>0.69 (-0.13; 0.85)	0.63 (0.19)<br>0.69 (-0.02; 0.89)<br>*n=55*	0.55 (0.25)<br>0.66 (0.06; 0.8)
**EQ VAS, mean (SD), median (min; max)**	61.1 (16.27)<br>65.0 (20.0; 90.0)	64.4 (17.17)<br>70.0 (17.0; 97.0)	60.1 (12.69)<br>60.0 (40.0; 80.0)
**EPDS score, mean (SD), median (min; max)**	7.6 (4.11)<br>7.0 (1.0; 19.0)<br>*n=54*	8.0 (4.97)<br>8.0 (0; 18.0)<br>*n=56*	7.6 (3.92)<br>7.5 (2.0; 14.0)
**CSQ-CAT, mean (SD), median (min; max)**	8.0 (6.65)<br>7.0 (0; 27.0)	6.7 (4.98)<br>7.0 (0; 24.0)	8.2 (6.41)<br>9.0 (0; 20.0)

TENS, Transcutaneous Electric Nerve Stimulation; SD, Standard Deviation; BMI, Body Mass Index (kg/m²); NRS, Numeric Rating Scale; LBP, Low Back Pain; PGP, Pelvic Girdle Pain
ODI, Oswestry Disability Index; PSFS, Patient-Specific Functional Scale (PSFS); PGQ, Pelvic Girdle Questionnaire EQ 5D, EuroQol 5 Dimensions; EQ VAS, EuroQol vertical visual analog scale; EDPS, Edinburgh Postnatal Depression Scale; CSQ-CAT, Coping Strategies Questionnaire subscale "Catastrophizing"

The sacroiliac joint was the most common pain location (93%) (Table 2).

Table. 2 Clinical tests to verify pelvic girdle pain

Variable	Acupuncture n=56	TENS n=57	Excluded after baseline test n=15
**Reported pubic pain, n (%)**			
No	20 (35.7)	20 (35.1)	5 (33.3)
Yes	36 (64.3)	37 (64.9)	10 (67.7)
**Reported sacroiliac pain, n (%)**			
No	4 (7.1)	4 (7.0)	1 (6.7)
Yes	52 (92.9)	53 (93.0)	14 (93.3)
**Localization sacroiliac pain, n (%)**			
Unilateral	6 (11.5)	7 (13.0)	2 (15.4)
Bilateral	46 (88.5)<br>*n=52*	47 (87.0)<br>*n=54*	11 (84.6<br>*n=13*
**Positive MAT-test, n (%)**			
No	29 (51.8)	29 (51.8)	8 (53.3)
Yes	27 (48.2)	27 (48.2)<br>*n=56*	7 (46.7)
**Positive 4P-test, n (%)**			
No	6 (10.7)	2 (3.5)	1 (6.6)
Yes, unilateral	9 (16.1)	16 (28.1)	4 (26.7)
Yes, bilateral	41 (73.2)	39 (68.4)	10 (66.7)

For peer review only - http://bmjopen.bmj.com/site/about/guidelines.xhtml

For peer review only

**Positive SIJ-separation, n (%)**			
No	24 (42.9)	24 (42.1)	5 (33.3)
Yes	32 (57.1)	33 (57.9)	10 (66.7)
**Positive SIJ-compression, n (%)**			
No	42 (75.0)	38 (66.7)	10 (66.7)
Yes	14 (25.0)	19 (33.3)	5 (33.3)
**Positive sacrum ventral-test, n (%)**			
No	29 (51.8)	31 (54.4)	8 (53.3)
Yes	27 (48.2)	26 (45.6)	7 (46.7)
**Positive ASLR-test, n (%)**			
No	10 (17.8)	8 (14)	4 (26.7)
Yes, unilateral	15 (26.8)	19 (33.4)	3 (20.0)
Yes, bilateral	31 (55.4)	30 (52.6)	8 (53.3)

TENS*= Transcutaneous electric nervous stimulation;* ***MAT****= Pulling a mat-test;* ***4P****: Posterior Pelvic Pain Provocation-test;* ***SIJ****= Sacro-iliac joint;* ***ASLR****= Active straight leg raise*

The women in both groups reported that their work ability was affected and few of them (acupuncture 23.2%; TENS 24.6%) met the general recommendations for PA. The consumption of analgesics was low in both groups (Table 1).

In both groups, participants were lost to follow up due to discontinuing the intervention (Fig. 1). Reasons for this was illness, vacation, need to care for younger children, difficulties to fit acupuncture sessions into work schedule or just dropping off. For acupuncture, the mean number (min;max) of treatment sessions was 9.9 (9;10) and women in the TENS group used the device for mean 26 (3;37) out of 35 days. There was no need to change the stimulation type due to insufficient effect for any of the participants in the TENS group. In the acupuncture group, 82.5% experienced positive effects of the treatment compared to 60% in the TENS group (p=0.032). At follow up, 9 of 41 women in the acupuncture group and 6 of 45 women in the TENS group reported some side effects during the intervention (Fig. 2).

Between group comparisons

No mean differences between groups were detected regarding the primary (disability, functioning, work ability, PA) or secondary outcome variables. Minor differences between the ITT and FAS were found (Table 3, 4).

For peer review only

For peer review only - http://bmjopen.bmj.com/site/about/guidelines.xhtml

Table 3. Change within groups and difference in change between the groups Acupuncture and TENS (ITT-analysis, multiple imputation of 50 imputed datasets)

	Acupuncture		TENS		Difference between groups	
**Outcomes**	**Mean change (95% CI)**	**p-value**	**Mean change (95% CI)**	**p-value**	**Mean difference (95% CI)**	**p-value**
**ODI**	0.44 (-3.74 to 4.61)	0.84	3.53 (-0.79 to 7.85)	0.11	-3.09 (-9.36 to 3.17)	0.33
**PSFS**	0.82 (0.01 to 1.63)	0.048	-0.16 (-0.79 to 0.47)	0.62	0.98 (-0.03 to 1.99)	0.058
**Work ability NRS**	-1.39 (-2.54 to -0.24)	0.018	-1.27 (-1.92 to -0.62)	0.0001	-0.12 (-1.44 to 1.19)	0.86
**Recommended PA**	-0.03 (-0.20 to 0.14)	0.72	-0.03 (-0.20 to 0.13)	0.68	-0.01 (-0.13 to 0.11)	0.87
**PGQ total**	-0.57 (-6.43 to 5.29)	0.85	3.08 (-2.28 to 8.43)	0.26	-3.65 (-11.46 to 4.16)	0.36
**PGQ activity**	0.37 (-5.69 to 6.44)	0.90	3.54 (-1.94 to 9.03)	0.20	-3.17 (-11.14 to 4.80)	0.43
**PGQ symptoms**	-1.34 (-8.33 to 5.65)	0.71	1.73 (-4.12 to 7.58)	0.56	-3.07 (-12.17 to 6.04)	0.51
**Evening pain NRS**	-0.96 (-1.91 to -0.01)	0.049	-1.29 (-2.13 to -0.44)	0.003	0.33 (-0.99 to 1.64)	0.62
**Concern about pain NRS**	-1.44 (-2.31 to -0.57)	0.0012	-1.99 (-2.81 to -1.17)	<.0001	0.55 (-0.64 to 1.73)	0.36
**EQ 5D**	0.04 (-0.07 to 0.14)	0.51	-0.10 (-0.21 to 0.00)	0.059	0.14 (-0.01 to 0.28)	0.061
**EQ 5D vertical VAS**	-1.18 (-7.72 to 5.37)	0.72	-1.66 (-8.79 to 5.48)	0.65	0.48 (-9.29 to 10.25)	0.92
**EPDS**	-0.40 (-2.07 to 1.28)	0.64	-1.25 (-2.77 to 0.27)	0.11	0.85 (-1.36 to 3.07)	0.45
**CSQ-CAT**	-2.50 (-5.43 to 0.43)	0.095	-1.21 (-2.68 to 0.25)	0.10	-1.29 (-4.55 to 1.98)	0.44

TENS, Transcutaneous Electric Nerve Stimulation; SD, Standard Deviation; BMI, Body Mass Index (kg/m²); NRS, Numeric Rating Scale; LBP,Low Back Pain; PGP, Pelvic Girdle Pain.
ODI, Oswestry Disability Index; PSFS, Patient-Specific Functional Scale; PGQ, Pelvic Girdle Questionnaire EQ 5D, EuroQol 5 Dimensions; EQ VAS, EuroQol vertical visual analog scale; EDPS, Edinburgh Postnatal Depression Scale; CSQ-CAT, Coping Strategies Questionnaire subscale "Catastrophizing"

For peer review only - http://bmjopen.bmj.com/site/about/guidelines.xhtml

Table 4. Change within groups and difference in change between the groups Acupuncture and TENS (Full analysis set, all available data)

	Acupuncture (n=41)				TENS (n=45)				Difference between groups	
**Outcome**	**Baseline Mean (SD) Median (Min; Max)**	**Follow up Mean (SD) Median (Min; Max)**	**Mean change (SD) Median (Min; Max) (95% CI for Mean)* n=**	**p-value**	**Baseline Mean (SD) Median (Min; Max)**	**Follow up Mean (SD) Median (Min; Max)**	**Mean change (SD) Median (Min; Max) (95% CI for Mean)* n=**	**p-value**	**Mean difference ** (95% CI)*****	**p-value**
**ODI**	32.59 (11.40)<br>34.00 (8.0; 60.0)	33.07 (13.41)<br>34.0 (10.0; 66.0)	0.49 (11.89)<br>2 (-22; 26)<br>(-3.20; 4.44)<br>n=41	0.80	29.07 (10.86)<br>28.0 (8.0; 60.0)	32.93 (15.24)<br>32.0 (2.0; 64.0)	3.87 (12.36)<br>4 (-28; 34)<br>(0.18; 7.68)<br>n=45	0.043	-3.38 (-8.53; 1.88)	0.21
**PSFS**	2.74 (1.54)<br>2.5 (0; 7.0)	3.63 (2.34)<br>3.5 (0; 10.0)	0.89 (2.45)<br>0 (-3; 6.5)<br>(0.11; 1.67)<br>n=41	0.025	3.16 (1.45)<br>3.0 (0.5; 7.5)	3.14 (1.75)<br>3.0 (0; 8.0)	-0.017 (1.85)<br>0 (-4.5; 4.5)<br>(-0.57; 0.54)<br>n=45	0.95	0.91 (0.00; 1.85)	0.053
**Work ability NRS**	6.45 (2.39)<br>7.0 (0; 10.0)	5.34 (3.11)<br>6.0 (0; 10.0)	-1.10 (3.13)<br>-1 (-8; 6)<br>(-2.12; -0.11)<br>n=40	0.036	7.0 (2.28)<br>7.0 (0; 10.0)	5.67 (2.54)<br>6.0 (0; 10)	-1.33 (1.92)<br>-1 (-6; 2)<br>(-1.91; -0.76)<br>n=45	<.0001	0.23 (-0.88; 1.31)	0.70
**Recommended PA; n (%)**	9 (22.0)	8 (19.5)	-0.02 (0.42)<br>0 (-1; 1)<br>(-0.15; 0.11)<br>n=41	1.00	10 (22.2)	9 (20.0)	-0.02 (0.40)<br>0 (-1; 1)<br>(-0.14; 0.10)<br>n=45	1.00	-0.002 (-0.17; 0.17)	1.00
**PGQ total**	46.68 (16.30)<br>48.64 (8.0; 89.72)	47.30 (16.32)<br>51.02 (12.0; 80.0)	0.82 (16.26)<br>3 (-34.67; 32)<br>(-4.51; 6.18)<br>n=39	0.76	45.25 (11.76)<br>42.16 (22.22; 69.33)	48.30 (16.74)<br>47.82 (12.50; 81.94)	3.84 (14.49)<br>2.05 (-38.17; 34.67)<br>(-0.48; 8.17)<br>n=44	0.086	-3.03 (-9.74; 3.63)	0.37
**PGQ activity**	44.93 (15.72<br>48.33 (6.67; 75.0)	47.02 (17.10)<br>49.12 (11.67; 78.94)	2.37 (15.65)<br>3.33 (-31.76; 31.67)<br>(-2.77; 7.44)<br>n=39	0.35	44.56 (12.16)<br>42.72 (24.56; 70.0)	48.00 (17.05)<br>46.67 (10.52; 82.45)	4.24 (14.81)<br>3.42 (-37.81; 33.33)<br>(-0.21; 8.66)<br>n=44	0.064	-1.87 (-8.50; 4.76)	0.58
**PGQ symptoms**	49.83 (16.40)<br>53.33 (13.33; 86.67)	48.42 (18.10)<br>48.33 (13.33; 86.67)	-1.45 (20.67)<br>0 (-53.33; 33.33)<br>(-8.24; 5.21)<br>n=39	0.66	48.00 (13.38)<br>46.67 (13.33; 73.33)	49.48 (18.12)<br>46.667 (0; 86.67)	1.48 (17.69)<br>6.67 (-40; 40)<br>(-3.86; 6.67)<br>n=45	0.58	-2.93 (-11.33; 5.56)	0.50
**Evening pain NRS**	6.82 (1.8)<br>7.0 (2.0; 9.5)	5.58 (2.26)<br>6.0 (1.0; 9.0)	-1.26 (2.64)<br>-1 (-7; 4)<br>(-2.12; -0.40)<br>n=39	0.0052	6.60 (1.5)<br>7.0 (3.0; 9.0)	5.33 (2.36)<br>6.0 (0; 9.0)	-1.27 (2.27)<br>-1 (-9; 3)<br>(-1.94; -0.60)<br>n=45	0.0002	0.01 (-1.06; 1.07)	1.00

For peer review only

**Concern about pain NRS**	3.95 (2.24)<br>5.0 (0; 8.0)	2.43 (2.15)<br>2.0 (0; 7.0)	-1.45 (2.43)<br>-2 (-7; 6)<br>(-2.25; -0.68)<br>n=40	0.0007	4.62 (2.41)<br>5.0 (0; 10.0)	2.62 (2.20)<br>2.0 (0; 8.0)	-2.00 (2.52)<br>-2 (-8; 2)<br>(-2.77; -1.25)<br>n=45	<.0001	0.55 (-0.52; 1.63)	0.32
**EQ 5D**	0.58 (0.25)<br>0.69 (-0.13; 0.85)	0.62 (0.23)<br>0.69 (-0.07; 0.88)	0.03 (0.32)<br>0 (-0.76; 0.75)<br>(-0.08; 0.13)<br>n=39	0.61	0.65 (0.18)<br>0.69 (-0.02; 0.89)	0.55 (0.29)<br>0.66 (-0.13; 1.0)	-0.10 (0.31)<br>-0.04 (-0.91; 0.61)<br>(-0.20; -0.01)<br>n=44	0.036	0.13 (-0.01; 0.27)	0.073
**EQ 5D vertical VAS**	62.24 (16.39)<br>65.0 (30.0; 90.0)	63.17 (16.38)<br>65.0 (25.0; 90.0)	0.93 (20.274)<br>0 (-47; 50)<br>(-5.60; 7.28)<br>n=41	0.78	66.20 (15.16)<br>70.0 (27.0; 95.0)	63.78 (19.51)<br>70.0 (19.0; 90.0)	-2.42 (19.75)<br>-1 (-55; 47)<br>(-8.25; 3.45)<br>n=45	0.42	3.35 (-5.31; 11.75)	0.45
**EPDS**	6.83 (3.99)<br>6.0 (1.0; 15.0)	6,15 (4.73)<br>5.0 (0; 15.0)	-0.85 (4.27)<br>-1 (-10; 11)<br>(-2.25; 0.52)<br>n=39	0.24	7.38 (4.91)<br>6.0 (0; 18.0)	6.27 (4.93)<br>5.0 (0; 21.0)	-1.11 (4.46)<br>0 (-12; 9)<br>(-2.43; 0.24)<br>n=45	0.11	0.27 (-1.65; 2.17)	0.79
**CSQ-CAT**	7.15 (5.98)<br>6.0 (0; 27)	4.49 (5.44)<br>2.0 (0; 18.0)	-2.60 (6.00)<br>-2.5 (-15; 12)<br>(-4.50; -0.70)<br>n=40	0.010	6.07 (4.83)<br>6.0 (0; 24.0)	5.11 (5.84)<br>4.0 (0; 28.0)	-1.09 (4.50)<br>-1 (-10; 17)<br>(-2.44; 0.27)<br>n=44	0.12	-1.51 (-3.82; 0.78)	0.20

* For comparison within groups the Fisher´s Non-Parametric Permutation Test for matched pairs was used.
** For comparison between groups the Fisher's Non Parametric Permutation Test was used for continuous variables.
*** The confidence interval for then mean difference between groups is based on Fisher's Non-Parametric Permutation Test.

TENS, Transcutaneous Electric Nerve Stimulation; SD, Standard Deviation; BMI, Body Mass Index (kg/m²); NRS, Numeric Rating Scale; LBP, Low Back Pain; PGP, Pelvic Girdle Pain ODI, Oswestry Disability Index; PSFS, Patient-Specific Functional Scale (PSFS); PGQ, Pelvic Girdle Questionnaire EQ 5D, EuroQol 5 Dimensions; EQ VAS, EuroQol vertical visual analog scale; EDPS, Edinburgh Postnatal Depression Scale; CSQ-CAT, Coping Strategies Questionnaire subscale "Catastrophizing"

For peer review only

For peer review only - http://bmjopen.bmj.com/site/about/guidelines.xhtml

Within group comparisons

For ODI or the level of PA, no significant change from baseline to follow-up was detected for any group. Both groups experienced a significant decrease in work ability at follow up. The acupuncture group showed a significant improvement in functioning, measured by PSFS, mean change 0.82 (p=0.048) (Table 3, 4).

Both groups reported significantly reduced evening pain intensity (acupuncture -0.96, p=0.049 and TENS -1.29, p=0.003) and concerns about pain (acupuncture -1.44, p=0.0012 and TENS -1.99, p<0.001) at follow-up. (Table 3, 4).

There were only minor differences between the ITT and FAS and this did not affect the results except for ODI and CSQ-CAT. For ODI the TENS group showed a significant increase of disability in the FAS but this was not the case in the ITT analysis. Regarding CSQ-CAT the acupuncture group, according to the FAS, reported a significant decrease in catastrophizing beliefs, this was not significant in the ITT.

Discussion

The results of the present study did not show any clinically important differences between the interventions regarding the primary outcome variables.

The measurements of disability (ODI) and daily function (PGQ) showed that PGP resulted in a moderate disability and affected the women's everyday life in various ways. Disability due to PGP/LBP tends to increase over time in pregnancy, [14] but in the present study no difference at follow up was detected. By PSFS, the functional status was assessed in an individually tailored way as the women themselves chose the measured activities. The reported activities covered for example housework, childcare, transportation, specific working tasks and leisure activities. There was a significant difference on PSFS at follow up for the acupuncture group, although it may have a small clinical relevance. For PSFS, the MCID varies between 0.8

For peer review only - http://bmjopen.bmj.com/site/about/guidelines.xhtml

(small change) and 3.0 in different studies and for different conditions.[48] At present, there are no studies that have evaluated PSFS in pregnancy. Both groups experienced a significant decrease of work ability (Table 3, 4). As previously shown, work ability is affected by a variety of factors in addition to PGP as pregnancy proceeds such as work conditions, general health, bodily changes and tiredness.[49] This result indicates that pregnancy itself has an impact on women's work ability.[14]

At follow-up, the level of PA was maintained for both groups (Table 3, 4). This differs from previous studies that showed that the level of activity decreases as pregnancy proceeds.[11,50-53] The interventions did reduce evening pain and concern about pain and this reduction may help women to maintain their PA level. The amount of evening pain relief in the present study could be considered small; mean change -0.96 for acupuncture and -1.29 for TENS (Table 3, 4) but a minimal clinical important difference (MCID) of -1.3 is reported as meaningful for pregnant women with PGP.[54] No clinically important difference could be detected between or within groups regarding health. Previous studies show that pregnant women, especially women with PGP and/or LBP, experience lower health compared to non-pregnant women [55] and also that quality of life decreases as pregnancy proceeds.[56] The results of the present study may indicate that a decrease in pain intensity and concern about pain could result in maintenance of overall functioning in daily activities, PA and, consequently, better health.

The women experienced positive effect of the treatments, acupuncture 82.5 % and TENS 60%. This difference could be explained by that the participants in the acupuncture group had more contact with the physiotherapist. The interaction between practitioner and patient may affect the outcome of the intervention [57] and we cannot clearly say that the two interventions alone caused the positive effects experienced by the participants. In this study, both interventions gave significant pain relief but no significant difference between the groups was detected. The increase of functioning (PSFS) at follow up for the acupuncture group could

For peer review only - http://bmjopen.bmj.com/site/about/guidelines.xhtml

indicate that the women experienced a positive impact on everyday activities that were important for them which in turn led to a positive experience of the intervention (Table 3, 4). A positive effect on activity level could be as relevant for the individual woman as pain relief, which could be important to consider when treatment choices are made.

Women with PGP experience a loss of independence as the pain makes them rely on other people for help, for example with child care and household tasks.[24] Treatment with TENS gives a woman control over her pain relief; she can use it where or when she wants. By contrast, for acupuncture, she must make appointments and go to a clinic for treatment. Some women may need more support and may benefit from regular visits to a physiotherapy clinic whereas others feel confident to perform self-treatment after receiving instructions and information about their complaints. The present study indicates that treatment of PGP during pregnancy can make women feel less concern about their pain. This may lead to less distress and better ability to cope with and manage the pain. Women experiencing higher levels of anxiety/depressive symptoms reported higher levels of disability compared to women with lower levels [19] and anxiety predicted lower levels of PA during pregnancy.[58] These are factors important to identify and manage to prevent the development of longstanding PGP that occur in 10% of women who experienced PGP during pregnancy.[8,9]

Strengths and limitations

Strengths of the present study are that the interventions were evaluated in both the second and the third trimester and that PGP was clinically verified in all participants. Another strength is the compliance among those who completed the interventions. In the acupuncture group, 38 out of 41 women (93 %) had the assigned ten sessions (mean 9.9). One participant experienced pain relief after 6 sessions and the intervention was not continued due to ethical considerations, two participants missed a session each due to business travel and vacation. The women in the TENS group used the treatment at home for mean 26 out of 35 possible

For peer review only

days during the treatment period. Only a few minor side effects were reported (Fig. 2), mostly at the beginning of the intervention period and none of them made the women discontinue treatment. Both TENS and acupuncture are according to previous studies safe to use during pregnancy. [25,27]

It is possible that the design of the present study, where the women had to travel to a specific physiotherapy clinic for testing, acupuncture, and follow-up, made women with more severe complaints decline to participate.[59] Another limitation of the present study is the self-reported use of TENS. Since the treatment was not monitored, it is possible that the women applying TENS treatment at home did not follow the instructions even though they followed protocol by registering the sessions. Previous research has reported that there is a risk for developing tolerance against the analgesic effect of TENS after a few days and a solution for this could be to change electrode placement and/or mode of stimulation during the treatment period.[60,61] In the present study, the mode of stimulation or the electrode placement could be changed by the treating physiotherapist after one week if the woman had insufficient analgesic effect, but this was not needed for any of the participants. Nevertheless, the participants experienced positive effects of the treatment.

Conclusion

No differences regarding the effect of acupuncture or TENS as treatment for PGP in pregnancy were detected in the present study. However, both interventions resulted in maintained PA level as well as reduced evening pain and concern about pain. The acupuncture group showed a change in functioning at follow up as well as higher satisfaction with treatment. This study suggests that, based on individual women's preferences and needs, these interventions could be chosen as non-pharmacological alternatives for pain relief in order to maintain functioning and PA level and consequently, maintain health as pregnancy proceeds.

For peer review only - http://bmjopen.bmj.com/site/about/guidelines.xhtml

Acknowledgements

The authors thank the participating pregnant women, the midwives for their help in recruiting the participants, the physiotherapists that provided the treatments and Nils-Gunnar Pehrsson for statistical advice.

Author Contributions

Concept/idea/research design: A. Gutke, M. Fagevik Olsén, T. Jendman

Writing: A. Svahn Ekdahl, A.Gutke, M. Fagevik Olsén

Data collection: A. Svahn Ekdahl, T. Jendman

Data analysis: A. Svahn Ekdahl, A. Gutke, M. Fagevik Olsén

Project management: A. Gutke, M. Fagevik Olsén, A. Svahn Ekdahl

Consultation (including review of manuscript before submitting): A. Gutke, M. Fagevik Olsén, T. Jendman

Competing interests

None declared

Funding

This study was financed by Region Västra Götaland, Sweden No. VGFOUREG-310521. The sponsor of the study did not have any influence on study design, collection and analysis/interpretation of data or writing the manuscript.

Data availability statement

The data on which the analyzes presented in this manuscript are based, are available upon reasonable request from the corresponding author.

For peer review only - http://bmjopen.bmj.com/site/about/guidelines.xhtml

421 **Trial registration**

422 The trial was registered in “FoU i Sverige” (R&D in Sweden), No. 127261
423 (https://www.researchweb.org/is/sverige/project/127261)

424

For peer review only

For peer review only - http://bmjopen.bmj.com/site/about/guidelines.xhtml

References

1. Vermani E, Mittal R, Weeks A. Pelvic girdle pain and low back pain in pregnancy: a review. *Pain Pract.* 2010;10(1):60-71.
2. Wu WH, Meijer OG, Uegaki K, et al. Pregnancy-related pelvic girdle pain (PPP), I: Terminology, clinical presentation, and prevalence. *Eur Spine J.* 2004;13(7):575-589.
3. Gutke A, Boissonnault J, Brook G, Stuge B. The Severity and Impact of Pelvic Girdle Pain and Low-Back Pain in Pregnancy: A Multinational Study. *J Womens Health (Larchmt).* 2018;27(4):510-517.
4. Vleeming A, Albert HB, Ostgaard HC, Sturesson B, Stuge B. European guidelines for the diagnosis and treatment of pelvic girdle pain. *Eur Spine J.* 2008;17(6):794-819.
5. Dionne CE, Dunn KM, Croft PR, et al. A consensus approach toward the standardization of back pain definitions for use in prevalence studies. *Spine (Phila Pa 1976).* 2008;33(1):95-103.
6. Aldabe D, Milosavljevic S, Bussey MD. Is pregnancy related pelvic girdle pain associated with altered kinematic, kinetic and motor control of the pelvis? A systematic review. *Eur Spine J.* 2012;21(9):1777-1787.
7. Noren L, Ostgaard S, Johansson G, Ostgaard HC. Lumbar back and posterior pelvic pain during pregnancy: a 3-year follow-up. *Eur Spine J.* 2002;11(3):267-271.
8. Elden H, Gutke A, Kjellby-Wendt G, Fagevik-Olsen M, Ostgaard HC. Predictors and consequences of long-term pregnancy-related pelvic girdle pain: a longitudinal follow-up study. *BMC Musculoskelet Disord.* 2016;17:276.
9. Bergstrom C, Persson M, Nergard KA, Mogren I. Prevalence and predictors of persistent pelvic girdle pain 12 years postpartum. *BMC Musculoskelet Disord.* 2017;18(1):399.
10. World Health Organisation (WHO). Global recommendations on physical activity for health. 2010; http://www.who.int/dietphysicalactivity/publications/9789241599979/en/.
11. Gjestland K, Bo K, Owe KM, Eberhard-Gran M. Do pregnant women follow exercise guidelines? Prevalence data among 3482 women, and prediction of low-back pain, pelvic girdle pain and depression. *Br J Sports Med.* 2013;47(8):515-520.
12. Lardon E, St-Laurent A, Babineau V, Descarreaux M, Ruchat SM. Lumbopelvic pain, anxiety, physical activity and mode of conception: a prospective cohort study of pregnant women. *BMJ Open.* 2018;8(11):e022508.
13. Sydsjo A, Alexanderson K, Dastserri M, Sydsjo G. Gender differences in sick leave related to back pain diagnoses: influence of pregnancy. *Spine (Phila Pa 1976).* 2003;28(4):385-389.
14. Gutke A, Olsson CB, Vollestad N, Oberg B, Wikmar LN, Robinson HS. Association between lumbopelvic pain, disability and sick leave during pregnancy - a comparison of three Scandinavian cohorts. *J Rehabil Med.* 2014;46(5):468-474.
15. Backhausen M, Damm P, Bendix J, Tabor A, Hegaard H. The prevalence of sick leave: Reasons and associated predictors - A survey among employed pregnant women. *Sex Reprod Healthc.* 2018;15:54-61.
16. Hansen ML, Thulstrup AM, Juhl M, Kristensen JK, Ramlau-Hansen CH. Predictors of sickness absence in pregnancy: a Danish cohort study. *Scand J Work Environ Health.* 2015;41(2):184-193.
17. Shiri R, Coggon D, Falah-Hassani K. Exercise for the prevention of low back and pelvic girdle pain in pregnancy: A meta-analysis of randomized controlled trials. *European Journal of Pain (United Kingdom).* 2018;22(1):19-27.
18. Gutke A, Josefsson A, Oberg B. Pelvic girdle pain and lumbar pain in relation to postpartum depressive symptoms. *Spine (Phila Pa 1976).* 2007;32(13):1430-1436.
19. Virgara R, Maher C, Van Kessel G. The comorbidity of low back pelvic pain and risk of depression and anxiety in pregnancy in primiparous women. *BMC Pregnancy Childbirth.* 2018;18(1):288.
20. Davenport MH, McCurdy AP, Mottola MF, et al. Impact of prenatal exercise on both prenatal and postnatal anxiety and depressive symptoms: a systematic review and meta-analysis. *Br J Sports Med.* 2018;52(21):1376-1385.

For peer review only - http://bmjopen.bmj.com/site/about/guidelines.xhtml

21. Davenport MH, Ruchat SM, Poitras VJ, et al. Prenatal exercise for the prevention of gestational diabetes mellitus and hypertensive disorders of pregnancy: a systematic review and meta-analysis. *Br J Sports Med.* 2018;52(21):1367-1375.

22. Lupattelli A, Spigset O, Twigg MJ, et al. Medication use in pregnancy: a cross-sectional, multinational web-based study. *BMJ Open.* 2014;4(2):e004365.

23. Mitchell AA, Gilboa SM, Werler MM, Kelley KE, Louik C, Hernandez-Diaz S. Medication use during pregnancy, with particular focus on prescription drugs: 1976-2008. *Am J Obstet Gynecol.* 2011;205(1):51.e51-58.

24. Mackenzie J, Murray E, Lusher J. Women's experiences of pregnancy related pelvic girdle pain: A systematic review. *Midwifery.* 2018;56:102-111.

25. Liddle SD, Pennick V. Interventions for preventing and treating low-back and pelvic pain during pregnancy. *Cochrane Database Syst Rev.* 2015(9):Cd001139.

26. Gutke A, Betten C, Degerskar K, Pousette S, Fagevik Olsen M. Treatments for pregnancy-related lumbopelvic pain: a systematic review of physiotherapy modalities. *Acta Obstet Gynecol Scand.* 2015;94(11):1156-1167.

27. Keskin EA, Onur O, Keskin HL, Gumus, II, Kafali H, Turhan N. Transcutaneous electrical nerve stimulation improves low back pain during pregnancy. *Gynecol Obstet Invest.* 2012;74(1):76-83.

28. Johnson M. Transcutaneous Electrical Nerve Stimulation: Mechanisms, Clinical Application and Evidence. *Rev Pain.* 2007;1(1):7-11.

29. Lund I, Lundeberg T. Mechanisms of acupuncture. 2016;4(4):26-30.

30. Shah S, Banh ET, Koury K, Bhatia G, Nandi R, Gulur P. Pain Management in Pregnancy: Multimodal Approaches. *Pain Res Treat.* 2015;2015:987483.

31. Carter RE, Lubinsky J. Rehabilitation Research: Principles and Applications. 5th ed. St Louis, MO: Elsevier; 2016:110-111.

32. Gutke A, Kjellby-Wendt G, Oberg B. The inter-rater reliability of a standardised classification system for pregnancy-related lumbopelvic pain. *Man Ther.* 2010;15(1):13-18.

33. Springer BA, Marin R, Cyhan T, Roberts H, Gill NW. Normative values for the unipedal stance test with eyes open and closed. *J Geriatr Phys Ther.* 2007;30(1):8-15.

34. Elden H, Ladfors L, Olsen MF, Ostgaard HC, Hagberg H. Effects of acupuncture and stabilising exercises as adjunct to standard treatment in pregnant women with pelvic girdle pain: randomised single blind controlled trial. *Bmj.* 2005;330(7494):761.

35. Fairbank JC, Pynsent PB. The Oswestry Disability Index. *Spine (Phila Pa 1976).* 2000;25(22):2940-2952; discussion 2952.

36. Westaway MD, Stratford PW, Binkley JM. The patient-specific functional scale: validation of its use in persons with neck dysfunction. *J Orthop Sports Phys Ther.* 1998;27(5):331-338.

37. de Zwart BC, Frings-Dresen MH, van Duivenbooden JC. Test-retest reliability of the Work Ability Index questionnaire. *Occup Med (Lond).* 2002;52(4):177-181.

38. Haakstad LA, Voldner N, Henriksen T, Bo K. Physical activity level and weight gain in a cohort of pregnant Norwegian women. *Acta Obstet Gynecol Scand.* 2007;86(5):559-564.

39. Stuge B, Garratt A, Krogstad Jenssen H, Grotle M. The pelvic girdle questionnaire: a condition-specific instrument for assessing activity limitations and symptoms in people with pelvic girdle pain. *Phys Ther.* 2011;91(7):1096-1108.

40. Gutke A, Stuge B, Elden H, Sandell C, Asplin G, Fagevik Olsen M. The Swedish version of the pelvic girdle questionnaire, cross-cultural adaptation and validation. *Disabil Rehabil.* 2019:1-8.

41. Hjermstad MJ, Fayers PM, Haugen DF, et al. Studies comparing Numerical Rating Scales, Verbal Rating Scales, and Visual Analogue Scales for assessment of pain intensity in adults: a systematic literature review. *J Pain Symptom Manage.* 2011;41(6):1073-1093.

42. Elkins G, Staniunas R, Rajab MH, Marcus J, Snyder T. Use of a numeric visual analog anxiety scale among patients undergoing colorectal surgery. *Clin Nurs Res.* 2004;13(3):237-244.

43. Rabin R, de Charro F. EQ-5D: a measure of health status from the EuroQol Group. *Ann Med.* 2001;33(5):337-343.

For peer review only

For peer review only - http://bmjopen.bmj.com/site/about/guidelines.xhtml

44. Kozinszky Z, Dudas RB. Validation studies of the Edinburgh Postnatal Depression Scale for the antenatal period. *J Affect Disord.* 2015;176:95-105.
45. Rubertsson C, Borjesson K, Berglund A, Josefsson A, Sydsjo G. The Swedish validation of Edinburgh Postnatal Depression Scale (EPDS) during pregnancy. *Nord J Psychiatry.* 2011;65(6):414-418.
46. Rosenstiel AK, Keefe FJ. The use of coping strategies in chronic low back pain patients: Relationship to patient characteristics and current adjustment. *Pain.* 1983;17(1):33-44.
47. Jensen I, Linton S. Coping Strategies Questionnaire (CSQ): Reliability of the Swedish version of the CSQ. *Scandinavian Journal of Behaviour Therapy.* 1993;22:139-145.
48. Horn KK, Jennings S, Richardson G, Vliet DV, Hefford C, Abbott JH. The patient-specific functional scale: psychometrics, clinimetrics, and application as a clinical outcome measure. *J Orthop Sports Phys Ther.* 2012;42(1):30-42.
49. Truong BT, Lupattelli A, Kristensen P, Nordeng H. Sick leave and medication use in pregnancy: A European web-based study. *BMJ Open.* 2017;7(8).
50. Gaston A, Vamos CA. Leisure-time physical activity patterns and correlates among pregnant women in Ontario, Canada. *Matern Child Health J.* 2013;17(3):477-484.
51. Walsh JM, McGowan C, Byrne J, McAuliffe FM. Prevalence of physical activity among healthy pregnant women in Ireland. *Int J Gynaecol Obstet.* 2011;114(2):154-155.
52. Evenson KR, Savitz DA, Huston SL. Leisure-time physical activity among pregnant women in the US. *Paediatr Perinat Epidemiol.* 2004;18(6):400-407.
53. Hegaard HK, Damm P, Hedegaard M, et al. Sports and leisure time physical activity during pregnancy in nulliparous women. *Matern Child Health J.* 2011;15(6):806-813.
54. Ogollah R, Bishop A, Lewis M, Grotle M, Foster NE. Responsiveness and Minimal Important Change for Pain and Disability Outcome Measures in Pregnancy-Related Low Back and Pelvic Girdle Pain. *Phys Ther.* 2019.
55. Olsson C, Nilsson-Wikmar L. Health-related quality of life and physical ability among pregnant women with and without back pain in late pregnancy. *Acta Obstet Gynecol Scand.* 2004;83(4):351-357.
56. Sahrakorpi N, Koivusalo SB, Stach-Lempinen B, Eriksson JG, Kautiainen H, Roine RP. "The Burden of Pregnancy"; heavier for the heaviest? The changes in Health Related Quality of Life (HRQoL) assessed by the 15D instrument during pregnancy and postpartum in different body mass index groups: a longitudinal survey. *Acta Obstet Gynecol Scand.* 2017;96(3):352-358.
57. Blasini M, Peiris N, Wright T, Colloca L. The Role of Patient-Practitioner Relationships in Placebo and Nocebo Phenomena. *Int Rev Neurobiol.* 2018;139:211-231.
58. St-Laurent A, Lardon E, Babineau V, Ruchat SM. Reproductive history, maternal anxiety and past physical activity practice predict physical activity levels throughout pregnancy. *Prev Med Rep.* 2019;16:100992.
59. Morino S, Ishihara M, Umezaki F, et al. Low back pain and causative movements in pregnancy: a prospective cohort study. *BMC Musculoskelet Disord.* 2017;18(1):416.
60. Liebano RE, Rakel B, Vance CGT, Walsh DM, Sluka KA. An investigation of the development of analgesic tolerance to TENS in humans. *Pain.* 2011;152(2):335-342.
61. Johnson MI. Resolving Long-Standing Uncertainty about the Clinical Efficacy of Transcutaneous Electrical Nerve Stimulation (TENS) to Relieve Pain: A Comprehensive Review of Factors Influencing Outcome. *Medicina (Kaunas).* 2021;57(4).

For peer review only

For peer review only - http://bmjopen.bmj.com/site/about/guidelines.xhtml

Figure legends.

Figure 1. Flowchart of the study according to the CONSORT requirements

Figure 2. Reported side effects of the interventions, Acupuncture (n=9/41) and TENS (n=6/45)

For peer review only - http://bmjopen.bmj.com/site/about/guidelines.xhtml

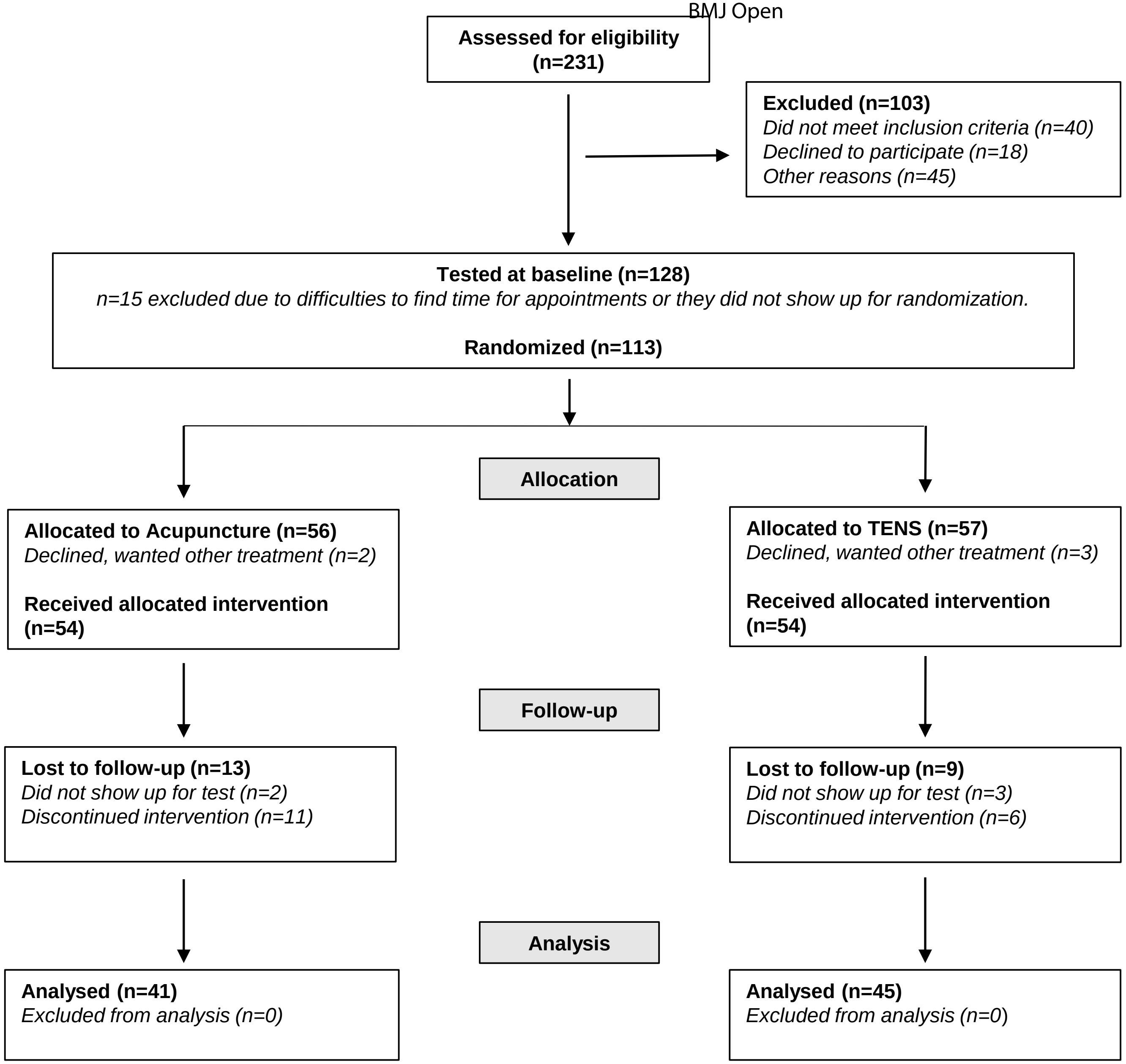
Assessed for eligibility (n=231)
Excluded (n=103)
Did not meet inclusion criteria (n=40)
Declined to participate (n=18)
Other reasons (n=45)
Tested at baseline (n=128)
n=15 excluded due to difficulties to find time for appointments or they did not show up for randomization.
Randomized (n=113)
Allocation
Allocated to Acupuncture (n=56)
Declined, wanted other treatment (n=2)
Received allocated intervention (n=54)
Allocated to TENS (n=57)
Declined, wanted other treatment (n=3)
Received allocated intervention (n=54)
Follow-up
Lost to follow-up (n=13)
Did not show up for test (n=2)
Discontinued intervention (n=11)
Lost to follow-up (n=9)
Did not show up for test (n=3)
Discontinued intervention (n=6)
Analysis
Analysed (n=41)
Excluded from analysis (n=0)
Analysed (n=45)
Excluded from analysis (n=0)

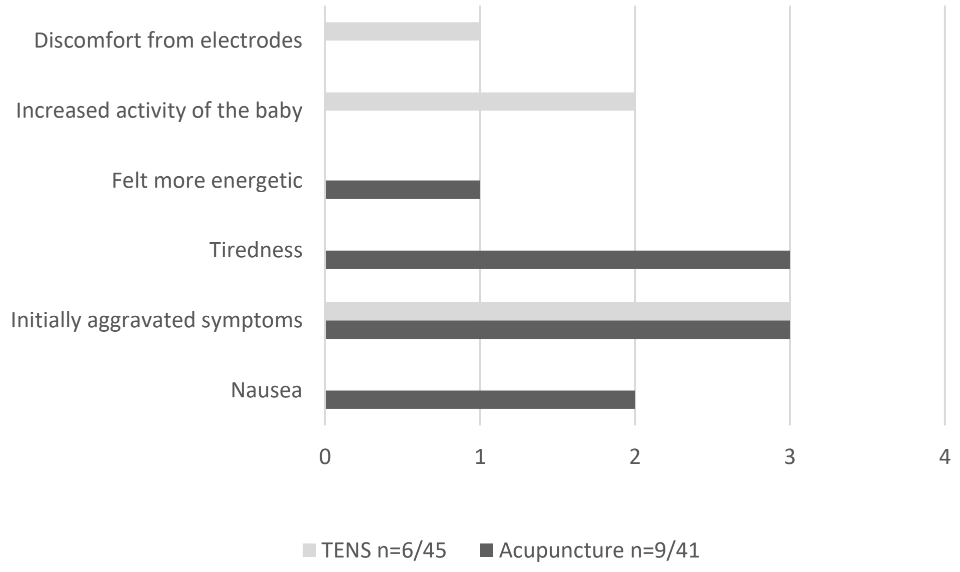
Discomfort from electrodes
Increased activity of the baby
Felt more energetic
Tiredness
Initially aggravated symptoms
Nausea
0
1
2
3
4
TENS n=6/45
Acupuncture n=9/41

STandards for Reporting Interventions in Clinical Trials of Acupuncture (STRICTA): extending the CONSORT Statement

"Maintenance of physical activity level, functioning and health after non-pharmacological treatment of pelvic girdle pain with either transcutaneous electric nerve stimulation or acupuncture. A randomized controlled trial"

Table 1: STRICTA 2010 checklist of information to include when reporting interventions in a clinical trial of acupuncture (Expansion of Item 5 from CONSORT 2010 checklist)

Item	Detail	Reported on page no:
**1. Acupuncture rationale**	1a) Style of acupuncture (e.g. Traditional Chinese Medicine, Japanese, Korean, Western medical, Five Element, ear acupuncture, etc)	08
	1b) Reasoning for treatment provided, based on historical context, literature sources, and/or consensus methods, with references where appropriate	06
	1c) Extent to which treatment was varied	
**2. Details of needling**	2a) Number of needle insertions per subject per session (mean and range where relevant)	08
	2b) Names (or location if no standard name) of points used (uni/bilateral)	08
	2c) Depth of insertion, based on a specified unit of measurement, or on a particular tissue level	08
	2d) Response sought (e.g. *de qi* or muscle twitch response)	08
	2e) Needle stimulation (e.g. manual, electrical)	08
	2f) Needle retention time	08
	2g) Needle type (diameter, length, and manufacturer or material)	08
**3. Treatment regimen**	3a) Number of treatment sessions	08
	3b) Frequency and duration of treatment sessions	08
**4. Other components of treatment**	4a) Details of other interventions administered to the acupuncture group (e.g. moxibustion, cupping, herbs, exercises, lifestyle advice)	08
	4b) Setting and context of treatment, including instructions to practitioners, and information and explanations to patients	
**5. Practitioner background**	5) Description of participating acupuncturists (qualification or professional affiliation, years in acupuncture practice, other relevant experience)	08
**6. Control or comparator interventions**	6a) Rationale for the control or comparator in the context of the research question, with sources that justify this choice	06
	6b) Precise description of the control or comparator. If sham acupuncture or any other type of acupuncture-like control is used, provide details as for Items 1 to 3 above.	09

Note: This checklist, which should be read in conjunction with the explanations of the STRICTA items provided in the main text, is designed to replace CONSORT 2010's item 5 when reporting an acupuncture trial.

Citation: *MacPherson H, Altman DG, Hammerschlag R, Youping L, Taixiang W, White A, Moher D; STRICTA Revision Group. Revised STandards for Reporting Interventions in Clinical Trials of Acupuncture (STRICTA): extending the CONSORT statement. PLoS Med. 2010 Jun 8;7(6):e1000261*

For peer review only - http://bmjopen.bmj.com/site/about/guidelines.xhtml

STandards for Reporting Interventions in Clinical Trials of Acupuncture (STRICTA): extending the CONSORT Statement

Table 2: CONSORT 2010 checklist with the Non-pharmacological Trials Extension to CONSORT (with STRICTA 2010 extending CONSORT Item 5 for acupuncture trials)

**Section/Topic**	**Item #**	**CONSORT 2010 Statement*: Checklist item[10]. Describe:**	**Additional items from the Non-pharmacological Trials Extension to CONSORT[14]. Add:**	**Reported on page no:**
TITLE AND ABSTRACT				
	1.a	Identification as a randomized trial in the title	In the abstract, description of the experimental treatment, comparator, care providers, centres and blinding status.	01
	1.b	Structured summary of trial design, methods, results, and conclusions; for specific guidance see CONSORT for Abstracts [58,59]		02–03
INTRODUCTION				
Background and objectives	2.a	Scientific background and explanation of rationale		03–04
	2.b	Specific objectives or hypotheses		03–04
METHODS				
Trial design	3.a	Description of trial design (e.g., parallel, factorial) including allocation ratio		06–07
	3.b	Important changes to methods after trial commencement (e.g. eligibility criteria), with reasons		n a
Participants	4.a	Eligibility criteria for participants	When applicable, eligibility criteria for centers and those performing the interventions.	07
	4.b	Settings and locations where the data were collected		07
**Interventions**	**5**	**The interventions for each group with sufficient details to allow replication, including how and when they were actually administered**	Precise details of both the experimental treatment and comparator - see Table 1 for details	**Details described in table 1**
Outcomes	6.a	Completely defined pre-specified primary and secondary outcome measures, including how and when they were assessed		09–10
	6.b	Any changes to trial outcomes after the trial commenced with reasons		n a
Sample size	7.a	How sample size was determined	When applicable, details of whether and how the clustering by care providers or centers was addressed.	10
	7.b	When applicable, explanation of any interim analyses and stopping guidelines		n a

For peer review only - http://bmjopen.bmj.com/site/about/guidelines.xhtml

STandards for Reporting Interventions in Clinical Trials of Acupuncture (STRICTA): extending the CONSORT Statement

Section/Topic	Item #	CONSORT 2010 Statement*: Checklist item[10]. Describe:	Additional items from the Non-pharmacological Trials Extension to CONSORT[14]. Add:	Reported on page no:
Randomization				
Sequence generation	8.a	Method used to generate the random allocation sequence	When applicable, how care providers were allocated to each trial group.	08
	8.b	Type of randomization; details of any restriction (e.g., blocking and block size)		08
Allocation concealment	9	Mechanism used to implement the random allocation sequence (e.g., sequentially numbered containers), describing any steps taken to conceal the sequence until interventions were assigned		08
Implementation	10	Who generated the random allocation sequence, who enrolled participants, and who assigned participants to interventions		08
Blinding	11.a	If done, who was blinded after assignment to interventions (e.g. participants, care providers, those assessing outcomes) and how	Whether or not those administering co-interventions were blinded to group assignment. If blinded, method of blinding and description of the similarity of interventions.	10
	11.b	If relevant, description of the similarity of interventions		n a
Statistical methods	12.a	Statistical methods used to compare groups for primary and secondary outcomes	When applicable, details of whether and how the clustering by care providers or centers was addressed.	10–11
	12.b	Methods for additional analyses, such as subgroup analyses and adjusted analyses		10–11
RESULTS				
Participant flow (A diagram is strongly recommended)	13.a	For each group, the numbers of participants who were randomly assigned, received intended treatment, and were analyzed for the primary outcome	The number of care providers or centers performing the intervention in each group and the number of patients treated by each care provider or in each center.	11, fig. 1
	13.b	For each group, losses and exclusions after randomization, together with reasons		11, fig. 1
Implementation of intervention			Details of the experimental treatment and comparator as they were implemented.	13
Recruitment	14.a	Dates defining the periods of recruitment and follow-up		7–8

For peer review only - http://bmjopen.bmj.com/site/about/guidelines.xhtml

STandards for Reporting Interventions in Clinical Trials of Acupuncture (STRICTA): extending the CONSORT Statement

Section/Topic	Item #	CONSORT 2010 Statement*: Checklist item[10]. Describe:	Additional items from the Non-pharmacological Trials Extension to CONSORT[14]. Add:	Reported on page no:
	14.b	Why the trial ended or was stopped		n a
Baseline data	15	A table showing baseline demographic and clinical characteristics for each group	When applicable, a description of care providers (case volume, qualification, expertise, etc.) and centers (volume) in each group.	11, table 1
Numbers analyzed	16	For each group, number of participants (denominator) included in each analysis and whether the analysis was by original assigned groups		Table 3, 4
Outcomes and estimation	17.a	For each primary and secondary outcome, results for each group, and the estimated effect size and its precision (e.g., 95% confidence interval)		Table 3, 4
	17.b	For binary outcomes, presentation of both absolute and relative effect sizes is recommended		Table 3, 4
Ancillary analyses	18	Results of any other analyses performed, including subgroup analyses and adjusted analyses, distinguishing pre-specified from exploratory		n a
Harms	19	All important harms or unintended effects in each group; for specific guidance see CONSORT for Harms [60]		13, fig. 2
DISCUSSION				
Limitations	20	Trial limitations, addressing sources of potential bias, imprecision, and, if relevant, multiplicity of analyses		17–20
Generalizability	21	Generalizability (external validity, applicability) of the trial findings	Generalizability (external validity) of the trial findings according to the intervention, comparators, patients and care providers and centers involved in the trial.	17–20

For peer review only

STandards for Reporting Interventions in Clinical Trials of Acupuncture (STRICTA): extending the CONSORT Statement

Section/Topic	Item #	CONSORT 2010 Statement*: Checklist item[10]. Describe:	Additional items from the Non-pharmacological Trials Extension to CONSORT[14]. Add:	Reported on page no:
Interpretation	22	Interpretation consistent with results, balancing benefits and harms, and considering other relevant evidence	In addition, take into account the choice of the comparator, lack of or partial blinding, unequal expertise of care providers or centers in each group.	20
OTHER INFORMATION				
Registration	23	Registration number and name of trial registry		03, 21
Protocol	24	Where the full trial protocol can be accessed, if available		n a
Funding	25	Sources of funding and other support (e.g., supply of drugs); role of funders		21

* We strongly recommend reading this Statement in conjunction with the CONSORT 2010 explanation and elaboration [11] for important clarifications on all the items. If relevant, we also recommend reading CONSORT extensions for cluster randomized trials [61], noninferiority and equivalence trials [62], herbal interventions [63], and pragmatic trials [16]. Moreover, additional extensions are forthcoming. For those and also for up-to-date references relevant to this checklist, see http://www.consort-statement.org.

For peer review only

BMJ Open

Maintenance of physical activity level, functioning and health after non-pharmacological treatment of pelvic girdle pain with either transcutaneous electric nerve stimulation or acupuncture. A randomized controlled trial

Journal:	*BMJ Open*
Manuscript ID	bmjopen-2020-046314.R2
Article Type:	Original research
Date Submitted by the Author:	14-Sep-2021
Complete List of Authors:	Svahn Ekdahl, Annika; Sahlgrenska Academy, Department of Health and Rehabilitation; Physiotherapy<br>Olsen, Monika Fagevik; Sahlgrenska universitetssjukhuset, Physiotherapy; Sahlgrenska Academy, Department of Health and Rehabilitation; Physiotherapy<br>Jendman, Tove; Physiotherapy Clinic "I Rorelse"<br>Gutke, Annelie; Sahlgrenska Academy, Department of Health and Rehabilitation; Physiotherapy
**Primary Subject Heading**:	Obstetrics and gynaecology
Secondary Subject Heading:	General practice / Family practice
Keywords:	PAIN MANAGEMENT, Maternal medicine < OBSTETRICS, Back pain < ORTHOPAEDIC & TRAUMA SURGERY, Musculoskeletal disorders < ORTHOPAEDIC & TRAUMA SURGERY

SCHOLARONE™
Manuscripts

I, the Submitting Author has the right to grant and does grant on behalf of all authors of the Work (as defined in the below author licence), an exclusive licence and/or a non-exclusive licence for contributions from authors who are: i) UK Crown employees; ii) where BMJ has agreed a CC-BY licence shall apply, and/or iii) in accordance with the terms applicable for US Federal Government officers or employees acting as part of their official duties; on a worldwide, perpetual, irrevocable, royalty-free basis to BMJ Publishing Group Ltd ("BMJ") its licensees and where the relevant Journal is co-owned by BMJ to the co-owners of the Journal, to publish the Work in this journal and any other BMJ products and to exploit all rights, as set out in our licence.

The Submitting Author accepts and understands that any supply made under these terms is made by BMJ to the Submitting Author unless you are acting as an employee on behalf of your employer or a postgraduate student of an affiliated institution which is paying any applicable article publishing charge ("APC") for Open Access articles. Where the Submitting Author wishes to make the Work available on an Open Access basis (and intends to pay the relevant APC), the terms of reuse of such Open Access shall be governed by a Creative Commons licence – details of these licences and which Creative Commons licence will apply to this Work are set out in our licence referred to above.

Other than as permitted in any relevant BMJ Author's Self Archiving Policies, I confirm this Work has not been accepted for publication elsewhere, is not being considered for publication elsewhere and does not duplicate material already published. I confirm all authors consent to publication of this Work and authorise the granting of this licence.

For peer review only

Maintenance of physical activity level, functioning and health after non-pharmacological treatment of pelvic girdle pain with either transcutaneous electric nerve stimulation or acupuncture. A randomized controlled trial

Annika Svahn Ekdahl[1], Monika Fagevik Olsén[1,2,] Tove Jendman[3], Annelie Gutke[1]

[1] Institute of Neuroscience and Physiology, Section of Health and Rehabilitation, Physiotherapy, Sahlgrenska Academy, University of Gothenburg, Gothenburg, Sweden

[2] Department of Physiotherapy, Sahlgrenska University Hospital, Gothenburg, Sweden

[3] Physiotherapy clinic "I Rörelse," Gothenburg, Sweden

Correspondence to:
Annika Svahn Ekdahl
University of Gothenburg
Institute of Neuroscience and Physiology
Department of Physiotherapy
Box 455
405 30 Gothenburg
Sweden
Phone: +46 31 786 5724 Mobile: +46 766 185724
E-mail: annika.svahn-ekdahl@neuro.gu.se

Word count; Abstract: 299, Body of manuscript: 3600

For peer review only - http://bmjopen.bmj.com/site/about/guidelines.xhtml

For peer review only

Abstract

Objective

To investigate if there are differences between acupuncture and transcutaneous electric nerve stimulation (TENS) as treatment for pelvic girdle pain (PGP) in pregnancy in order to manage pain and thus maintain health and functioning in daily activities and physical activity (PA).

Design

Randomized controlled trial

Setting and participants

Pregnant women (n=113) with clinically verified PGP in gestational weeks 12–28, recruited from maternity healthcare centers, randomized (1:1) into two groups. Exclusion criteria: Any obstetric complication, systemic disease or previous disorder that could contradict tests or treatment.

Interventions

The intervention consisted of either ten acupuncture sessions (two sessions per week) provided by a physiotherapist or daily home-based TENS during five weeks.

Primary outcome variables

Disability (Oswestry Disability Index), functioning (Patient Specific Functional Scale), work ability (Work Ability Index) and PA-level according to general recommendations.

Secondary outcome variables

Functioning related to PGP (Pelvic Girdle Questionnaire), evening pain intensity (Numeric Rating Scale, NRS), concern about pain (NRS), health (EuroQoL 5D), symptoms of

For peer review only

depression/catastrophizing (Edinburgh Postnatal Depression Scale/Coping Strategies Questionnaire).

Results

No mean differences were detected between the groups. Both groups managed to preserve their functioning and PA level at follow up. This may be due to significantly ($p<0.05$) reduced within groups evening pain intensity; acupuncture -0.96 (95% CI -1.91 to -0.01; p=0.049), TENS -1.29 (95% CI -2.13 to -0.44; p=0.003) and concern about pain; acupuncture -1.44 (95% CI -2.31 to -0.57; p=0.0012), TENS -1.99 (95% CI -2.81 to -1.17; $p<0.0001$). The acupuncture group showed an improvement in functioning at follow up; 0.82 (95% CI 0.01 to 1.63; p=0.048)

Conclusion

Treating PGP with acupuncture or TENS resulted in maintenance of functioning and physical activity and also less pain and concern about pain. Either intervention could be recommended as a non-pharmacological alternative for pain relief and may enable pregnant women to stay active.

Trial registration: In "FoU i Sverige" (R&D in Sweden) No. 12726.
https://www.researchweb.org/is/sverige/project/127261

Article Summary

Strengths and limitations of this study

- All participants had clinically verified PGP during pregnancy.
- Interventions were evaluated both in the 2nd and 3rd trimester of pregnancy, with good adherence in both intervention groups.

For peer review only - http://bmjopen.bmj.com/site/about/guidelines.xhtml

- Lower self-reported satisfaction in the TENS group could be a result of less contact with the treating physiotherapist than in the acupuncture group.
- It is possible that the participants did not use TENS according to instructions, as this was self-reported.
- The study design, where pregnant women had to travel to the clinic for assessment and/or intervention, may have affected the ability to recruit women with more severe PGP to the study.

For peer review only

For peer review only - http://bmjopen.bmj.com/site/about/guidelines.xhtml

Introduction

Pelvic girdle pain (PGP) and/or low back pain (LBP) is reported internationally by over 50% of pregnant women.[1-3] PGP has musculoskeletal origin and is localized to the area between the posterior iliac crest and the gluteal fold, most commonly around the sacroiliac joints and/or pubic bone, sometimes with radiating pain in the thighs.[4] Limitations in daily functioning and walking difficulties, especially a feeling of "catching" of the leg are common symptoms.[2] These symptoms differ from those associated with LBP, which is defined as pain in the area between the lower ribs and the iliac crest, with or without radiating pain to the legs, and is often described as worse when sitting.[5]

The etiology of PGP is multifactorial. Increased pregnancy-related ligamentous laxity may result in altered mechanics in the pelvic joints.[6] Pain may occur if these alterations cannot be compensated for by neuromuscular control.[4] PGP most commonly occurs around gestation week 18, but can occur at any time during pregnancy. Among women who experience PGP during pregnancy, about 20% suffer from persistent pain 4 months postpartum.[7] Previous long-term studies have reported that approximately 10% suffer from remaining PGP more than 11 years postpartum.[8,9] Experiencing PGP during pregnancy is a risk factor for having PGP also in a next pregnancy.[2]

Pregnant women are advised to follow the general recommendations for physical activity (PA), of at least 150 minutes of moderate intensity or at least 75 minutes of high intensity PA per week or a combination of both.[10] Few pregnant women reach this level of PA and a common cause is PGP.[11] A high level of pain during pregnancy results in more severe limitations in daily life and less PA.[12]

There is a higher rate of sick leave among pregnant women compared to age-matched, non-pregnant women and a major reason is PGP and/or LBP.[13-15] Studies show a reduced risk for

For peer review only - http://bmjopen.bmj.com/site/about/guidelines.xhtml

sick leave if the woman can continue to stay active and exercise throughout her pregnancy.[16,17] Women with PGP and/or LBP have an increased risk of prenatal anxiety, depressive symptoms and postpartum depression compared to healthy pregnant women.[18,19] If pregnant women maintains the recommended levels of PA, both the odds of getting prenatal depression and the severity of it is reduced.[20] Furthermore, there is a risk in pregnancy for complications such as hypertension, diabetes and preeclampsia and this risk is reduced if the woman stays active.[21]

The use of medication in pregnancy has increased over the years [22,23] with a risk for overuse of analgesics among women having PGP.[24] Therefore, it is important to provide efficient treatment alternatives. The best evidence non-pharmacological treatments for PGP are acupuncture, a stabilizing pelvic belt, and physical exercise.[25,26] There is, however, limited evidence for the use of transcutaneous electrical nerve stimulation (TENS),[25-27] even if it is widely used in physiotherapy clinical practice. The physiological mechanisms of acupuncture and TENS are similar. Both peripheral and central mechanisms of pain control are stimulated, as per the gate control theory, activating descending pathways for pain control and release of endogenous opioids.[28,29] No specific adverse effects regarding use in pregnancy are reported for any of the interventions mentioned here.[30]

The aim of this study was to investigate if there are differences between acupuncture and TENS as treatments for PGP in order to manage pain and thus maintain health and functioning in daily activities and PA.

Methods

A randomized, controlled design was used to compare acupuncture and TENS as pain relief for PGP in pregnancy. The decision to randomize into two different groups was made based on ethical considerations. It is not ethical to withhold evidence based treatment, therefore the

For peer review only - http://bmjopen.bmj.com/site/about/guidelines.xhtml

For peer review only

experimental treatment (TENS) was given to one group and acupuncture, which is known to be effective for PGP [25,26], to an active control group.[31] The study was approved by the regional ethical review board in Gothenburg, Sweden (No. 308-13).

Setting and Participants

The data collection took place 2014–2018 in two Swedish cities. Two independent physiotherapists located in each city conducted tests and treatment. Women with PGP were briefly informed about the study at Maternity Health Care Centers and those who were interested were contacted by the independent test leader for detailed information and for initial screening for inclusion.

Inclusion criteria

- Single fetus pregnancy
- Pregnancy-related PGP or combined PGP/LBP in gestational weeks 12–28
- Pain located distally/laterally of L5/S1, over the buttocks, and/or the pubic bone, verified by ≥2 positive pain provocation tests or a positive active straight leg raise (ASLR)
- A score of ≥20% on the Oswestry Disability Index (ODI) and/or ≤6 in one self-chosen activity of the Patient-Specific Functional Scale (PSFS)
- Ability to read and understand Swedish

Exclusion criteria

- Previous fracture, surgery, or malignant disease in the back, pelvis, or hips
- Any systemic disease or obstetric complication that contraindicates treatment or tests
- Contraindications for TENS; pacemaker; decreased sensation in the treatment area
- Contraindications for acupuncture; treatment with anticoagulants
- Start of other treatment during the study period

For peer review only - http://bmjopen.bmj.com/site/about/guidelines.xhtml

Those eligible for participation underwent a clinical examination to verify PGP and answered the baseline questionnaire. All participants gave oral and written consent. The clinical examination consisted of a reliable procedure, which included a neurological examination, hip rotation range of motion test, pelvic pain provocation tests, a brief mechanical assessment of the lumbar spine and the ASLR test.[32] The examination also included a single-leg balance test.[33]

Randomization and Interventions

Before start of intervention, the participants each drew a sealed, opaque envelope, prepared from a computer-generated list by an independent research assistant, for assignment to either the acupuncture or TENS group (1:1).

The intervention lasted for 5 weeks in both groups. All women got written general advice on how to manage PGP in addition to the assigned treatment.

Acupuncture

The intervention consisted of 10 acupuncture sessions (two sessions per week), provided by an experienced physiotherapist, trained in Western medical acupuncture. It was decided, due to ethical considerations, not to continue the intervention for any woman that experienced sufficient pain relief in fewer than ten sessions. The treating physiotherapist chose the acupuncture points, based on the individual woman's symptoms and clinical presentation, from a protocol published in a previous study where point localization and insertion depth are described in detail.[34] Disposable, stainless steel needles (China Classic; 0.25x15 mm, 0.30x30 mm, 0.30x50 mm) were used. After insertion and manual stimulation to evoke the sensation of *de Qi* (a radiating sensation or a feeling of heaviness) from the insertion point, the needles were left in situ for 30 minutes and manually stimulated after 10 and 20 minutes. The

For peer review only - http://bmjopen.bmj.com/site/about/guidelines.xhtml

participants got written information about acupuncture at the start of treatment and were instructed to report any percieved side effects to the treating physiotherapist.

TENS

An experienced physiotherapist tried out the electrode placement according to the individual woman's symptoms and clinical presentation, either uni- or bilaterally over the sacroiliacal joint and gluteal muscles for dorsal pelvic pain or in the groin area for pubic pain. The women received oral and written instructions regarding their individual electrode placement and then used the TENS device (Cefar Primo PRO; DJO Nordic AB, Malmö, Sweden) at home for at least 30 minutes per day for 5 weeks. High frequency stimulation (80 Hz) was used with an intensity of the stimulation that produced a strong but comfortable sensation in the treated area. The women were instructed to increase the intensity of the stimulation during the treatment session in order to maintain the strong but comfortable sensation. Each woman met the physiotherapist on a follow-up visit after 1 week to discuss treatment and to adjust electrode placement and stimulation mode if needed. In case of absence of effect after the first week of treatment, the stimulation mode could be changed to low frequency (2 Hz) in order to receive optimal pain relief. After 3 weeks, one follow-up telephone call to each woman was made to ensure that the intervention was working well. After 5 weeks the participants made a final visit to the physiotherapist.

Outcomes and Follow-up

Because of the present study's focus on functioning, a broad spectrum of outcome variables was included. All outcomes were measured at baseline and repeated at follow up.

Primary outcomes

- Disability, defined as affected function due to PGP, measured by ODI, range 0–100%, where 0% = no disability.[35]

For peer review only - http://bmjopen.bmj.com/site/about/guidelines.xhtml

- Functional status, assessed by the PSFS. Two self-chosen everyday activities rated on a numeric rating scale (NRS) of 0–10; 10 = can perform the activity unrestrictedly or as before the onset of PGP.[36]
- Work ability, measured by one question from the Work Ability Index (WAI). Current work ability marked on a NRS of 0–10; 10 = work ability at its best.[37]
- Physical activity level, number of days/week with activity for 30 minutes at moderate intensity and/or activity at high intensity for 20 minutes.[38]

Secondary outcomes

- Everyday functioning affected by PGP, measured by the Pelvic Girdle Questionnaire (PGQ). A total score and scores for the subscales of "Activity" and "Symptoms", where 0% = no disability.[39,40]
- Evening PGP intensity at its worst, measured by a NRS of 0–10; 10 = worst pain,[41] and by a pain drawing to illustrate the pain location.
- Concern about PGP, assessed using a NRS of 0–10; 10 = extremely concerned.[42]
- Health, measured by the EuroQol 5-dimension questionnaire (EQ5D), a total score of 1.0 indicates full health. Participants also marked their current health status on the EuroQol vertical visual analog scale (EQ VAS); 100 = best imaginable health state.[43]
- Symptoms of depression, measured by the Edinburgh Postnatal Depression Scale (EPDS), range 0-30. A score >13 indicates symptoms of depression that require further examination.[44,45]
- Catastrophizing beliefs via the subscale "Catastrophizing" (from the Coping Strategies Questionnaire, CSQ-CAT) range 0-36; 0 = no catastrophizing beliefs.[46,47]
- Questions were also asked about use of analgesics, sick leave, height/weight, education level, family income, and possible side effects of the treatment.

For peer review only

For peer review only - http://bmjopen.bmj.com/site/about/guidelines.xhtml

After the intervention period the clinical examination was repeated by the independent test-leader, who was blinded to the treatment allocation, and the participants completed a follow-up questionnaire.

Patient and public involvement

No patients or members of the public was involved in design, conduction or interpretation of the study. Patient satisfaction was measured at follow up.

Statistical analysis

Sample size

With a β-level of 80%, an α-level of 5%, and a difference in functioning between groups of 10%, measured using the ODI, a sample size of at least 30 women/group was required. As the dropout rate for the postpartum follow-up and the planned 3.5 year long-term follow-up was calculated to be 25% and 50%, respectively, the plan was to include 60 women in each group.

Analysis

The analysis was done according to intention to treat (ITT), with the use of multiple imputation with 50 imputation datasets generated followed by one analysis of each of these 50 datasets followed by a pooled analysis of the 50 tests. The imputation of each value was performed by Fully Conditional Specifications. For continuous variables T-test was applied due to central limit theorem and for dichotomous variables change in proportion was applied. A Full Analysis Set (FAS) was also performed, using Fischer's non-parametric permutation test, including only those women who completed the intervention. Change from baseline to follow up is expressed as mean (SD)/ median (min; max)/ 95% CI for mean. For dichotomous variables, the Chi Square-test was used. The level of statistical significance was set to $p<0.05$.

For peer review only

The analysis was made using IBM SPSS Statistics v. 24 (IBM Corp., Armonk, NY, USA) and SAS v. 9.4 (SAS Institute, Cary, NC, USA).

Results

Altogether 15 women, who fulfilled the inclusion criteria, were excluded after the baseline test due to difficulties in finding time for treatment or they did not show up for randomization (Fig. 1). A total of 113 women were randomized into two groups (Fig. 1). No relevant differences between the intervention groups were found at baseline. At baseline, women in both groups reported pain in their pelvic and/or lumbar region during the last 4 weeks, which affected their daily activities for more than 1 day/week. (Table 1).

Table. 1 Descriptive data

Variable	Acupuncture n=56	TENS n=57	Excluded after baseline-test n=15
**Maternal age, mean (SD), median (min; max)**	30.5 (3.96)<br>30.0 (20.0; 38.0)	31.1 (4.38)<br>32.0 (22.0; 43.0)	31.9 (3.92)<br>32.0 (27.0; 40.0)
**Gestational weeks, mean (SD), median (min; max)**	20.9 (5.32)<br>21.5 (11.0; 28.0)	20.8 (5.18)<br>22.0 (10.0; 28.0)	22.4 (5.17)<br>24.0 (13.0; 28.0)
**BMI, mean (SD), median (min; max)**	25.6 (4.53)<br>25.0 (19.5; 41.8)	26.5 (3.87)<br>26.2 (19.7; 35.0)<br>*n=55*	27.6 (5.83)<br>27.6 (18.2; 41.0)
**Parity, n (%)**			
0	21 (37.5)	22 (38.6)	6 (40.0)
1	30 (53.6)	28 (49.1)	5 (33.3)
≥2	5 (8.9)	7 (12.3)	4 (26.7)
**Education level, n (%)**			
Elementary school	1 (1.8)	2 (3.5)	0
High school	12 (22.4)	20 (35.1)	3 (20.0)
Higher education	43 (76.8)	35 (61.4)	12 (80.0)
**Occupation, n (%)**			
Paid employment	47 (83.9)	47 (82.5)	14 (93.3)
Not in paid employment	3 (5.4)	3 (5.3)	0
Parental leave	0	2 (3.5)	0
Combination	2 (3.6)	3 (5.3)	0
Other	4 (7.1)	2 (3.5)	1 (6.7)
**Sick leave, n (%)**	9 (16.1)	7 (12.3)	3 (20.0)
**Manage financially, n (%)**			
Very well	22 (39.3)	34 (59.6)	7 (46.7)
Quite well	30 (53.6)	21 (36.8)	7 (46.7)
Neither well or badly	4 (7.1)	2 (3.5)	1 (6.7)
Quite badly	0	0	0
Very badly	0	0	0
**LBP/PGP last 4 weeks, n (%)**	56 (100)	57 (100)	15 (100)
**Pain affects daily activities, n (%)**	53 (94.6)	57 (100)	15 (100)

For peer review only - http://bmjopen.bmj.com/site/about/guidelines.xhtml

For peer review only

**Pain how often, n (%)**			
Some days	3 (5.4)	7 (12.3)	0
Most days	16 (28.6)	19 (33.3)	8 (53.3)
Every day	37 (66.1)	31 (54.4)	7 (46.7)
**Use of analgesics, n (%)**	6 (10.9)	3 (5.3)	0
**ODI, mean (SD),**<br>**median (min; max)**	*n=55*<br>34.5 (13.19)<br>34.0 (8.0; 68.0)	30.1 (11.82)<br>30.0 (8.0; 60.0)	32.5 (12.22)<br>32.0 (12.0; 52.0)
**PSFS NRS, mean (SD),**<br>**median (min; max)**	2.8 (1.46)<br>2.5 (0; 7.0)	3.2 (1.55)<br>3.0 (0; 7.5)	3.9 (2.33)<br>3.0 (0.5; 9.0)
**Work ability NRS, mean (SD),**<br>**median (min; max)**	6.2 (2.36)<br>7.0 (0; 10.0)	6.7 (2.41)<br>7.0 (0; 10.0)	5.7 (3.02)<br>6.0 (0; 9.0)
**Recommended PA; yes, n (%)**	*n=55*<br>13 (23.2)	*n=56*<br>14 (24.6)	6 (40)
**PGQ total, mean (SD),**<br>**median (min; max)**	49.3 (17.70)<br>50.7 (8.0; 90.7)	47.0 (12.91)<br>45.6 (22.2; 82.6))	53.2 (18.1)<br>60.0 (20.0; 77.3)
**PGQ activity, mean (SD),**<br>**median (min; max)**	*n=55*<br>48.1 (17.76)<br>50.0 (6.7; 90.0)	*n=56*<br>46.3 (13.55)<br>45.3 (24.6; 82.5)	52.1 (18.63)<br>58.3 (16.7; 76.7)
**PGQ symptom, mean (SD),**<br>**median (min; max)**	*n=55*<br>51.5 (17.17)<br>53.3 (13.3; 93.3)	*n=56*<br>49.4 (13.27)<br>46.7 (13.3; 73.3)	57.1 (15.98)<br>60.0 (26.67; 80.0)
**Evening pain intensity NRS, mean (SD),**<br>**median (min; max)**	*n=55*<br>6.8 (1.95)<br>7.0 (2.0; 10.0)	6.6 (1.56)<br>7.0 (3.0; 9.0)	7.4 (1.56)<br>8.0 (4.0; 9.0)
**Concern about pain NRS, mean (SD),**<br>**median (min; max)**	*n=55*<br>4.2 (2.44)<br>5.0 (0; 10.0)	4.6 (2.34)<br>5.0 (0; 10.0)	4.7 (2.69)<br>5.0 (0; 10.0)
**EQ 5D, mean (SD),**<br>**median (min; max)**	0.55 (0.28)<br>0.69 (-0.13; 0.85)	0.63 (0.19)<br>0.69 (-0.02; 0.89)	0.55 (0.25)<br>0.66 (0.06; 0.8)
**EQ VAS, mean (SD),**<br>**median (min; max)**	61.1 (16.27)<br>65.0 (20.0; 90.0)	*n=55*<br>64.4 (17.17)<br>70.0 (17.0; 97.0)	60.1 (12.69)<br>60.0 (40.0; 80.0)
**EPDS score, mean (SD),**<br>**median (min; max)**	7.6 (4.11)<br>7.0 (1.0; 19.0)	8.0 (4.97)<br>8.0 (0; 18.0)	7.6 (3.92)<br>7.5 (2.0; 14.0)
**CSQ-CAT, mean (SD),**<br>**median (min; max)**	*n=54*<br>8.0 (6.65)<br>7.0 (0; 27.0)	*n=56*<br>6.7 (4.98)<br>7.0 (0; 24.0)	8.2 (6.41)<br>9.0 (0; 20.0)

TENS, Transcutaneous Electric Nerve Stimulation; SD, Standard Deviation; BMI, Body Mass Index (kg/m²); NRS, Numeric Rating Scale; LBP, Low Back Pain; PGP, Pelvic Girdle Pain
ODI, Oswestry Disability Index; PSFS, Patient-Specific Functional Scale (PSFS); PGQ, Pelvic Girdle Questionnaire EQ 5D, EuroQol 5 Dimensions; EQ VAS, EuroQol vertical visual analog scale; EDPS, Edinburgh Postnatal Depression Scale; CSQ-CAT, Coping Strategies Questionnaire subscale "Catastrophizing"

The sacroiliac joint was the most common pain location (93%) (Table 2).

Table. 2 Clinical tests to verify pelvic girdle pain

Variable	Acupuncture<br>n=56	TENS<br>n=57	Excluded after baseline test<br>n=15
**Reported pubic pain, n (%)**			
No	20 (35.7)	20 (35.1)	5 (33.3)
Yes	36 (64.3)	37 (64.9)	10 (67.7)
**Reported sacroiliac pain, n (%)**			
No	4 (7.1)	4 (7.0)	1 (6.7)
Yes	52 (92.9)	53 (93.0)	14 (93.3)

For peer review only

**Localization sacroiliac pain, n (%)**			
Unilateral	6 (11.5)	7 (13.0)	2 (15.4)
Bilateral	46 (88.5)	47 (87.0)	11 (84.6
	n=52	*n=54*	*n=13*
**Positive MAT-test, n (%)**			
No	29 (51.8)	29 (51.8)	8 (53.3)
Yes	27 (48.2)	27 (48.2)	7 (46.7)
		n=56	
**Positive 4P-test, n (%)**			
No	6 (10.7)	2 (3.5)	1 (6.6)
Yes, unilateral	9 (16.1)	16 (28.1)	4 (26.7)
Yes, bilateral	41 (73.2)	39 (68.4)	10 (66.7)
**Positive SIJ-separation, n (%)**			
No	24 (42.9)	24 (42.1)	5 (33.3)
Yes	32 (57.1)	33 (57.9)	10 (66.7)
**Positive SIJ-compression, n (%)**			
No	42 (75.0)	38 (66.7)	10 (66.7)
Yes	14 (25.0)	19 (33.3)	5 (33.3)
**Positive sacrum ventral-test, n (%)**			
No	29 (51.8)	31 (54.4)	8 (53.3)
Yes	27 (48.2)	26 (45.6)	7 (46.7)
**Positive ASLR-test, n (%)**			
No	10 (17.8)	8 (14)	4 (26.7)
Yes, unilateral	15 (26.8)	19 (33.4)	3 (20.0)
Yes, bilateral	31 (55.4)	30 (52.6)	8 (53.3)

***TENS**= Transcutaneous electric nervous stimulation; **MAT**= Pulling a mat-test; **4P**: Posterior Pelvic Pain Provocation-test; **SIJ**= Sacro-iliac joint; **ASLR**= Active straight leg raise*

The women in both groups reported that their work ability was affected and few of them (acupuncture 23.2%; TENS 24.6%) met the general recommendations for PA. The consumption of analgesics was low in both groups (Table 1).

In both groups, participants were lost to follow up due to discontinuing the intervention (Fig. 1). Reasons for this was illness, vacation, need to care for younger children, difficulties to fit acupuncture sessions into work schedule or just dropping off. For acupuncture, the mean number (min;max) of treatment sessions was 9.9 (9;10) and women in the TENS group used the device for mean 26 (3;37) out of 35 days. There was no need to change the stimulation type due to insufficient effect for any of the participants in the TENS group. In the acupuncture group, 82.5% experienced positive effects of the treatment compared to 60% in the TENS group (p=0.032). At follow up, 9 of 41 women in the acupuncture group and 6 of 45 women in the TENS group reported some side effects during the intervention (Fig. 2).

For peer review only - http://bmjopen.bmj.com/site/about/guidelines.xhtml

For peer review only

Between group comparisons

No mean differences between groups were detected regarding the primary (disability, functioning, work ability, PA) or secondary outcome variables. Minor differences between the ITT and FAS were found (Table 3, 4).

Table 3. Change within groups and difference in change between the groups Acupuncture and TENS (ITT-analysis, multiple imputation of 50 imputed datasets)

	Acupuncture		TENS		Difference between groups	
**Outcomes**	**Mean change (95% CI)**	**p-value**	**Mean change (95% CI)**	**p-value**	**Mean difference (95% CI)**	**p-value**
**ODI**	0.44 (-3.74 to 4.61)	0.84	3.53 (-0.79 to 7.85)	0.11	-3.09 (-9.36 to 3.17)	0.33
**PSFS**	0.82 (0.01 to 1.63)	0.048	-0.16 (-0.79 to 0.47)	0.62	0.98 (-0.03 to 1.99)	0.058
**Work ability NRS**	-1.39 (-2.54 to -0.24)	0.018	-1.27 (-1.92 to -0.62)	0.0001	-0.12 (-1.44 to 1.19)	0.86
**Recommended PA**	-0.03 (-0.20 to 0.14)	0.72	-0.03 (-0.20 to 0.13)	0.68	-0.01 (-0.13 to 0.11)	0.87
**PGQ total**	-0.57 (-6.43 to 5.29)	0.85	3.08 (-2.28 to 8.43)	0.26	-3.65 (-11.46 to 4.16)	0.36
**PGQ activity**	0.37 (-5.69 to 6.44)	0.90	3.54 (-1.94 to 9.03)	0.20	-3.17 (-11.14 to 4.80)	0.43
**PGQ symptoms**	-1.34 (-8.33 to 5.65)	0.71	1.73 (-4.12 to 7.58)	0.56	-3.07 (-12.17 to 6.04)	0.51
**Evening pain NRS**	-0.96 (-1.91 to -0.01)	0.049	-1.29 (-2.13 to -0.44)	0.003	0.33 (-0.99 to 1.64)	0.62
**Concern about pain NRS**	-1.44 (-2.31 to -0.57)	0.0012	-1.99 (-2.81 to -1.17)	<.0001	0.55 (-0.64 to 1.73)	0.36
**EQ 5D**	0.04 (-0.07 to 0.14)	0.51	-0.10 (-0.21 to 0.00)	0.059	0.14 (-0.01 to 0.28)	0.061
**EQ 5D vertical VAS**	-1.18 (-7.72 to 5.37)	0.72	-1.66 (-8.79 to 5.48)	0.65	0.48 (-9.29 to 10.25)	0.92
**EPDS**	-0.40 (-2.07 to 1.28)	0.64	-1.25 (-2.77 to 0.27)	0.11	0.85 (-1.36 to 3.07)	0.45
**CSQ-CAT**	-2.50 (-5.43 to 0.43)	0.095	-1.21 (-2.68 to 0.25)	0.10	-1.29 (-4.55 to 1.98)	0.44

For peer review only - http://bmjopen.bmj.com/site/about/guidelines.xhtml

TENS, Transcutaneous Electric Nerve Stimulation; SD, Standard Deviation; BMI, Body Mass Index (kg/m²); NRS, Numeric Rating Scale; LBP,Low Back Pain; PGP, Pelvic Girdle Pain.
ODI, Oswestry Disability Index; PSFS, Patient-Specific Functional Scale; PGQ, Pelvic Girdle Questionnaire EQ 5D, EuroQol 5 Dimensions; EQ VAS, EuroQol vertical visual analog scale; EDPS, Edinburgh Postnatal Depression Scale; CSQ-CAT, Coping Strategies Questionnaire subscale "Catastrophizing"

For peer review only

For peer review only - http://bmjopen.bmj.com/site/about/guidelines.xhtml

Table 4. Change within groups and difference in change between the groups Acupuncture and TENS (Full analysis set, all available data)

	Acupuncture (n=41)				TENS (n=45)				Difference between groups	
**Outcome**	**Baseline<br>Mean (SD)<br>Median (Min; Max)**	**Follow up<br>Mean (SD)<br>Median (Min; Max)**	**Mean change (SD)<br>Median (Min; Max)<br>(95% CI for Mean)*<br>n=**	**p-value**	**Baseline<br>Mean (SD)<br>Median (Min; Max)**	**Follow up<br>Mean (SD)<br>Median (Min; Max)**	**Mean change (SD)<br>Median (Min; Max)<br>(95% CI for Mean)*<br>n=**	**p-value**	**Mean difference **<br>(95% CI)*****	**p-value**
**ODI**	32.59 (11.40)<br>34.00 (8.0; 60.0)	33.07 (13.41)<br>34.0 (10.0; 66.0)	0.49 (11.89)<br>2 (-22; 26)<br>(-3.20; 4.44)<br>n=41	0.80	29.07 (10.86)<br>28.0 (8.0; 60.0)	32.93 (15.24)<br>32.0 (2.0; 64.0)	3.87 (12.36)<br>4 (-28; 34)<br>(0.18; 7.68)<br>n=45	0.043	-3.38 (-8.53; 1.88)	0.21
**PSFS**	2.74 (1.54)<br>2.5 (0; 7.0)	3.63 (2.34)<br>3.5 (0; 10.0)	0.89 (2.45)<br>0 (-3; 6.5)<br>(0.11; 1.67)<br>n=41	0.025	3.16 (1.45)<br>3.0 (0.5; 7.5)	3.14 (1.75)<br>3.0 (0; 8.0)	-0.017 (1.85)<br>0 (-4.5; 4.5)<br>(-0.57; 0.54)<br>n=45	0.95	0.91 (0.00; 1.85)	0.053
**Work ability NRS**	6.45 (2.39)<br>7.0 (0; 10.0)	5.34 (3.11)<br>6.0 (0; 10.0)	-1.10 (3.13)<br>-1 (-8; 6)<br>(-2.12; -0.11)<br>n=40	0.036	7.0 (2.28)<br>7.0 (0; 10.0)	5.67 (2.54)<br>6.0 (0; 10)	-1.33 (1.92)<br>-1 (-6; 2)<br>(-1.91; -0.76)<br>n=45	<.0001	0.23 (-0.88; 1.31)	0.70
**Recommended PA; n (%)**	9 (22.0)	8 (19.5)	-0.02 (0.42)<br>0 (-1; 1)<br>(-0.15; 0.11)<br>n=41	1.00	10 (22.2)	9 (20.0)	-0.02 (0.40)<br>0 (-1; 1)<br>(-0.14; 0.10)<br>n=45	1.00	-0.002 (-0.17; 0.17)	1.00
**PGQ total**	46.68 (16.30)<br>48.64 (8.0; 89.72)	47.30 (16.32)<br>51.02 (12.0; 80.0)	0.82 (16.26)<br>3 (-34.67; 32)<br>(-4.51; 6.18)<br>n=39	0.76	45.25 (11.76)<br>42.16 (22.22; 69.33)	48.30 (16.74)<br>47.82 (12.50; 81.94)	3.84 (14.49)<br>2.05 (-38.17; 34.67)<br>(-0.48; 8.17)<br>n=44	0.086	-3.03 (-9.74; 3.63)	0.37
**PGQ activity**	44.93 (15.72<br>48.33 (6.67; 75.0)	47.02 (17.10)<br>49.12 (11.67; 78.94)	2.37 (15.65)<br>3.33 (-31.76; 31.67)<br>(-2.77; 7.44)<br>n=39	0.35	44.56 (12.16)<br>42.72 (24.56; 70.0)	48.00 (17.05)<br>46.67 (10.52; 82.45)	4.24 (14.81)<br>3.42 (-37.81; 33.33)<br>(-0.21; 8.66)<br>n=44	0.064	-1.87 (-8.50; 4.76)	0.58
**PGQ symptoms**	49.83 (16.40)<br>53.33 (13.33; 86.67)	48.42 (18.10)<br>48.33 (13.33; 86.67)	-1.45 (20.67)<br>0 (-53.33; 33.33)<br>(-8.24; 5.21)<br>n=39	0.66	48.00 (13.38)<br>46.67 (13.33; 73.33)	49.48 (18.12)<br>46.667 (0; 86.67)	1.48 (17.69)<br>6.67 (-40; 40)<br>(-3.86; 6.67)<br>n=45	0.58	-2.93 (-11.33; 5.56)	0.50
**Evening pain NRS**	6.82 (1.8)<br>7.0 (2.0; 9.5)	5.58 (2.26)<br>6.0 (1.0; 9.0)	-1.26 (2.64)<br>-1 (-7; 4)<br>(-2.12; -0.40)<br>n=39	0.0052	6.60 (1.5)<br>7.0 (3.0; 9.0)	5.33 (2.36)<br>6.0 (0; 9.0)	-1.27 (2.27)<br>-1 (-9; 3)<br>(-1.94; -0.60)<br>n=45	0.0002	0.01 (-1.06; 1.07)	1.00

For peer review only

For peer review only - http://bmjopen.bmj.com/site/about/guidelines.xhtml

**Concern about pain NRS**	3.95 (2.24)<br>5.0 (0; 8.0)	2.43 (2.15)<br>2.0 (0; 7.0)	-1.45 (2.43)<br>-2 (-7; 6)<br>(-2.25; -0.68)<br>n=40	0.0007	4.62 (2.41)<br>5.0 (0; 10.0)	2.62 (2.20)<br>2.0 (0; 8.0)	-2.00 (2.52)<br>-2 (-8; 2)<br>(-2.77; -1.25)<br>n=45	<.0001	0.55 (-0.52; 1.63)	0.32
**EQ 5D**	0.58 (0.25)<br>0.69 (-0.13; 0.85)	0.62 (0.23)<br>0.69 (-0.07; 0.88)	0.03 (0.32)<br>0 (-0.76; 0.75)<br>(-0.08; 0.13)<br>n=39	0.61	0.65 (0.18)<br>0.69 (-0.02; 0.89)	0.55 (0.29)<br>0.66 (-0.13; 1.0)	-0.10 (0.31)<br>-0.04 (-0.91; 0.61)<br>(-0.20; -0.01)<br>n=44	0.036	0.13 (-0.01; 0.27)	0.073
**EQ 5D vertical VAS**	62.24 (16.39)<br>65.0 (30.0; 90.0)	63.17 (16.38)<br>65.0 (25.0; 90.0)	0.93 (20.274)<br>0 (-47; 50)<br>(-5.60; 7.28)<br>n=41	0.78	66.20 (15.16)<br>70.0 (27.0; 95.0)	63.78 (19.51)<br>70.0 (19.0; 90.0)	-2.42 (19.75)<br>-1 (-55; 47)<br>(-8.25; 3.45)<br>n=45	0.42	3.35 (-5.31; 11.75)	0.45
**EPDS**	6.83 (3.99)<br>6.0 (1.0; 15.0)	6,15 (4.73)<br>5.0 (0; 15.0)	-0.85 (4.27)<br>-1 (-10; 11)<br>(-2.25; 0.52)<br>n=39	0.24	7.38 (4.91)<br>6.0 (0; 18.0)	6.27 (4.93)<br>5.0 (0; 21.0)	-1.11 (4.46)<br>0 (-12; 9)<br>(-2.43; 0.24)<br>n=45	0.11	0.27 (-1.65; 2.17)	0.79
**CSQ-CAT**	7.15 (5.98)<br>6.0 (0; 27)	4.49 (5.44)<br>2.0 (0; 18.0)	-2.60 (6.00)<br>-2.5 (-15; 12)<br>(-4.50; -0.70)<br>n=40	0.010	6.07 (4.83)<br>6.0 (0; 24.0)	5.11 (5.84)<br>4.0 (0; 28.0)	-1.09 (4.50)<br>-1 (-10; 17)<br>(-2.44; 0.27)<br>n=44	0.12	-1.51 (-3.82; 0.78)	0.20

* For comparison within groups the Fisher´s Non-Parametric Permutation Test for matched pairs was used.
** For comparison between groups the Fisher's Non Parametric Permutation Test was used for continuous variables.
*** The confidence interval for then mean difference between groups is based on Fisher's Non-Parametric Permutation Test.

TENS, Transcutaneous Electric Nerve Stimulation; SD, Standard Deviation; BMI, Body Mass Index (kg/m^2); NRS, Numeric Rating Scale; LBP, Low Back Pain; PGP, Pelvic Girdle Pain ODI, Oswestry Disability Index; PSFS, Patient-Specific Functional Scale (PSFS); PGQ, Pelvic Girdle Questionnaire EQ 5D, EuroQol 5 Dimensions; EQ VAS, EuroQol vertical visual analog scale; EDPS, Edinburgh Postnatal Depression Scale; CSQ-CAT, Coping Strategies Questionnaire subscale "Catastrophizing"

For peer review only

For peer review only - http://bmjopen.bmj.com/site/about/guidelines.xhtml

Within group comparisons

For ODI or the level of PA, no significant change from baseline to follow-up was detected for any group. Both groups experienced a significant decrease in work ability at follow up. The acupuncture group showed a significant improvement in functioning, measured by PSFS, mean change 0.82 (p=0.048) (Table 3, 4).

Both groups reported significantly reduced evening pain intensity (acupuncture -0.96, p=0.049 and TENS -1.29, p=0.003) and concerns about pain (acupuncture -1.44, p=0.0012 and TENS -1.99, p<0.001) at follow-up. (Table 3, 4).

There were only minor differences between the ITT and FAS and this did not affect the results except for ODI and CSQ-CAT. For ODI the TENS group showed a significant increase of disability in the FAS but this was not the case in the ITT analysis. Regarding CSQ-CAT the acupuncture group, according to the FAS, reported a significant decrease in catastrophizing beliefs, this was not significant in the ITT.

Discussion

The results of the present study did not show any clinically important differences between the interventions regarding the primary outcome variables.

The measurements of disability (ODI) and daily function (PGQ) showed that PGP resulted in a moderate disability and affected the women's everyday life in various ways. Disability due to PGP/LBP tends to increase over time in pregnancy, [14] but in the present study no difference at follow up was detected. By PSFS, the functional status was assessed in an individually tailored way as the women themselves chose the measured activities. The reported activities covered for example housework, childcare, transportation, specific working tasks and leisure activities. There was a significant difference on PSFS at follow up for the acupuncture group, although it may have a small clinical relevance. For PSFS, the MCID varies between 0.8

For peer review only - http://bmjopen.bmj.com/site/about/guidelines.xhtml

(small change) and 3.0 in different studies and for different conditions.[48] At present, there are no studies that have evaluated PSFS in pregnancy. Both groups experienced a significant decrease of work ability (Table 3, 4). As previously shown, work ability is affected by a variety of factors in addition to PGP as pregnancy proceeds such as work conditions, general health, bodily changes and tiredness.[49] This result indicates that pregnancy itself has an impact on women's work ability.[14]

At follow-up, the level of PA was maintained for both groups (Table 3, 4). This differs from previous studies that showed that the level of activity decreases as pregnancy proceeds.[11,50-53] The interventions did reduce evening pain and concern about pain and this reduction may help women to maintain their PA level. The amount of evening pain relief in the present study could be considered small; mean change -0.96 for acupuncture and -1.29 for TENS (Table 3, 4) but a minimal clinical important difference (MCID) of -1.3 is reported as meaningful for pregnant women with PGP.[54] No clinically important difference could be detected between or within groups regarding health. Previous studies show that pregnant women, especially women with PGP and/or LBP, experience lower health compared to non-pregnant women [55] and also that quality of life decreases as pregnancy proceeds.[56] The results of the present study may indicate that a decrease in pain intensity and concern about pain could result in maintenance of overall functioning in daily activities, PA and, consequently, better health.

The women experienced positive effect of the treatments, acupuncture 82.5 % and TENS 60%. This difference could be explained by that the participants in the acupuncture group had more contact with the physiotherapist. The interaction between practitioner and patient may affect the outcome of the intervention [57] and we cannot clearly say that the two interventions alone caused the positive effects experienced by the participants. In this study, both interventions gave significant pain relief but no significant difference between the groups was detected. The increase of functioning (PSFS) at follow up for the acupuncture group could,

For peer review only - http://bmjopen.bmj.com/site/about/guidelines.xhtml

even though it may be a small change[48], indicate that the women experienced a positive impact on everyday activities that were important for them which in turn led to a positive experience of the intervention (Table 3, 4). A positive effect on activity level could be as relevant for the individual woman as pain relief, which could be important to consider when treatment choices are made.

Women with PGP experience a loss of independence as the pain makes them rely on other people for help, for example with child care and household tasks.[24] Treatment with TENS gives a woman control over her pain relief; she can use it where or when she wants. By contrast, for acupuncture, she must make appointments and go to a clinic for treatment. Some women may need more support and may benefit from regular visits to a physiotherapy clinic whereas others feel confident to perform self-treatment after receiving instructions and information about their complaints. The present study indicates that treatment of PGP during pregnancy can make women feel less concern about their pain. This may lead to less distress and better ability to cope with and manage the pain. Women experiencing higher levels of anxiety/depressive symptoms reported higher levels of disability compared to women with lower levels [19] and anxiety predicted lower levels of PA during pregnancy.[58] These are factors important to identify and manage to prevent the development of longstanding PGP that occur in 10% of women who experienced PGP during pregnancy.[8,9]

Strengths and limitations

Strengths of the present study are that the interventions were evaluated in both the second and the third trimester and that PGP was clinically verified in all participants. Another strength is the compliance among those who completed the interventions. In the acupuncture group, 38 out of 41 women (93 %) had the assigned ten sessions (mean 9.9). One participant experienced pain relief after 6 sessions and the intervention was not continued due to ethical considerations, two participants missed a session each due to business travel and vacation.

For peer review only - http://bmjopen.bmj.com/site/about/guidelines.xhtml

The women in the TENS group used the treatment at home for mean 26 out of 35 possible days during the treatment period. Only a few minor side effects were reported (Fig. 2), mostly at the beginning of the intervention period and none of them made the women discontinue treatment. Both TENS and acupuncture are according to previous studies safe to use during pregnancy. [25,27]

It is possible that the design of the present study, where the women had to travel to a specific physiotherapy clinic for testing, acupuncture, and follow-up, made women with more severe complaints decline to participate.[59] Another limitation of the present study is the self-reported use of TENS. Since the treatment was not monitored, it is possible that the women applying TENS treatment at home did not follow the instructions even though they followed protocol by registering the sessions. Previous research has reported that there is a risk for developing tolerance against the analgesic effect of TENS after a few days and a solution for this could be to change electrode placement and/or mode of stimulation during the treatment period.[60,61] In the present study, the mode of stimulation or the electrode placement could be changed by the treating physiotherapist after one week if the woman had insufficient analgesic effect, but this was not needed for any of the participants. Nevertheless, the participants experienced positive effects of the treatment.

Conclusion

No differences regarding the effect of acupuncture or TENS as treatment for PGP in pregnancy were detected in the present study. However, both interventions resulted in maintained PA level as well as reduced evening pain and concern about pain. The acupuncture group showed a change in functioning at follow up as well as higher satisfaction with treatment. This study suggests that, based on individual women's preferences and needs, these interventions could be chosen as non-pharmacological alternatives for pain relief in

For peer review only - http://bmjopen.bmj.com/site/about/guidelines.xhtml

order to maintain functioning and PA level and consequently, maintain health as pregnancy proceeds.

Acknowledgements

The authors thank the participating pregnant women, the midwives for their help in recruiting the participants, the physiotherapists that provided the treatments and Nils-Gunnar Pehrsson for statistical advice.

Author Contributions

Concept/idea/research design: A. Gutke, M. Fagevik Olsén, T. Jendman

Writing: A. Svahn Ekdahl, A.Gutke, M. Fagevik Olsén

Data collection: A. Svahn Ekdahl, T. Jendman

Data analysis: A. Svahn Ekdahl, A. Gutke, M. Fagevik Olsén

Project management: A. Gutke, M. Fagevik Olsén, A. Svahn Ekdahl

Consultation (including review of manuscript before submitting): A. Gutke, M. Fagevik Olsén, T. Jendman

Competing interests

None declared

Funding

This study was financed by Region Västra Götaland, Sweden No. VGFOUREG-310521. The sponsor of the study did not have any influence on study design, collection and analysis/interpretation of data or writing the manuscript.

For peer review only

For peer review only - http://bmjopen.bmj.com/site/about/guidelines.xhtml

Trial registration

The trial was registered in "FoU i Sverige" (R&D in Sweden), No. 127261 (https://www.researchweb.org/is/sverige/project/127261)

Ethics statement

The study was approved by the regional ethical review board in Gothenburg, Sweden (No. 308-13).

Data sharing

The data on which the analyses presented in this manuscript are based, are available upon reasonable request from the corresponding author.

For peer review only

For peer review only - http://bmjopen.bmj.com/site/about/guidelines.xhtml

References

1. Vermani E, Mittal R, Weeks A. Pelvic girdle pain and low back pain in pregnancy: a review. *Pain Pract.* 2010;10(1):60-71.
2. Wu WH, Meijer OG, Uegaki K, et al. Pregnancy-related pelvic girdle pain (PPP), I: Terminology, clinical presentation, and prevalence. *Eur Spine J.* 2004;13(7):575-589.
3. Gutke A, Boissonnault J, Brook G, Stuge B. The Severity and Impact of Pelvic Girdle Pain and Low-Back Pain in Pregnancy: A Multinational Study. *J Womens Health (Larchmt).* 2018;27(4):510-517.
4. Vleeming A, Albert HB, Ostgaard HC, Sturesson B, Stuge B. European guidelines for the diagnosis and treatment of pelvic girdle pain. *Eur Spine J.* 2008;17(6):794-819.
5. Dionne CE, Dunn KM, Croft PR, et al. A consensus approach toward the standardization of back pain definitions for use in prevalence studies. *Spine (Phila Pa 1976).* 2008;33(1):95-103.
6. Aldabe D, Milosavljevic S, Bussey MD. Is pregnancy related pelvic girdle pain associated with altered kinematic, kinetic and motor control of the pelvis? A systematic review. *Eur Spine J.* 2012;21(9):1777-1787.
7. Noren L, Ostgaard S, Johansson G, Ostgaard HC. Lumbar back and posterior pelvic pain during pregnancy: a 3-year follow-up. *Eur Spine J.* 2002;11(3):267-271.
8. Elden H, Gutke A, Kjellby-Wendt G, Fagevik-Olsen M, Ostgaard HC. Predictors and consequences of long-term pregnancy-related pelvic girdle pain: a longitudinal follow-up study. *BMC Musculoskelet Disord.* 2016;17:276.
9. Bergstrom C, Persson M, Nergard KA, Mogren I. Prevalence and predictors of persistent pelvic girdle pain 12 years postpartum. *BMC Musculoskelet Disord.* 2017;18(1):399.
10. World Health Organisation (WHO). Global recommendations on physical activity for health. 2010; http://www.who.int/dietphysicalactivity/publications/9789241599979/en/.
11. Gjestland K, Bo K, Owe KM, Eberhard-Gran M. Do pregnant women follow exercise guidelines? Prevalence data among 3482 women, and prediction of low-back pain, pelvic girdle pain and depression. *Br J Sports Med.* 2013;47(8):515-520.
12. Lardon E, St-Laurent A, Babineau V, Descarreaux M, Ruchat SM. Lumbopelvic pain, anxiety, physical activity and mode of conception: a prospective cohort study of pregnant women. *BMJ Open.* 2018;8(11):e022508.
13. Sydsjo A, Alexanderson K, Dastserri M, Sydsjo G. Gender differences in sick leave related to back pain diagnoses: influence of pregnancy. *Spine (Phila Pa 1976).* 2003;28(4):385-389.
14. Gutke A, Olsson CB, Vollestad N, Oberg B, Wikmar LN, Robinson HS. Association between lumbopelvic pain, disability and sick leave during pregnancy - a comparison of three Scandinavian cohorts. *J Rehabil Med.* 2014;46(5):468-474.
15. Backhausen M, Damm P, Bendix J, Tabor A, Hegaard H. The prevalence of sick leave: Reasons and associated predictors - A survey among employed pregnant women. *Sex Reprod Healthc.* 2018;15:54-61.
16. Hansen ML, Thulstrup AM, Juhl M, Kristensen JK, Ramlau-Hansen CH. Predictors of sickness absence in pregnancy: a Danish cohort study. *Scand J Work Environ Health.* 2015;41(2):184-193.
17. Shiri R, Coggon D, Falah-Hassani K. Exercise for the prevention of low back and pelvic girdle pain in pregnancy: A meta-analysis of randomized controlled trials. *European Journal of Pain (United Kingdom).* 2018;22(1):19-27.
18. Gutke A, Josefsson A, Oberg B. Pelvic girdle pain and lumbar pain in relation to postpartum depressive symptoms. *Spine (Phila Pa 1976).* 2007;32(13):1430-1436.
19. Virgara R, Maher C, Van Kessel G. The comorbidity of low back pelvic pain and risk of depression and anxiety in pregnancy in primiparous women. *BMC Pregnancy Childbirth.* 2018;18(1):288.
20. Davenport MH, McCurdy AP, Mottola MF, et al. Impact of prenatal exercise on both prenatal and postnatal anxiety and depressive symptoms: a systematic review and meta-analysis. *Br J Sports Med.* 2018;52(21):1376-1385.

For peer review only - http://bmjopen.bmj.com/site/about/guidelines.xhtml

21. Davenport MH, Ruchat SM, Poitras VJ, et al. Prenatal exercise for the prevention of gestational diabetes mellitus and hypertensive disorders of pregnancy: a systematic review and meta-analysis. *Br J Sports Med.* 2018;52(21):1367-1375.
22. Lupattelli A, Spigset O, Twigg MJ, et al. Medication use in pregnancy: a cross-sectional, multinational web-based study. *BMJ Open.* 2014;4(2):e004365.
23. Mitchell AA, Gilboa SM, Werler MM, Kelley KE, Louik C, Hernandez-Diaz S. Medication use during pregnancy, with particular focus on prescription drugs: 1976-2008. *Am J Obstet Gynecol.* 2011;205(1):51.e51-58.
24. Mackenzie J, Murray E, Lusher J. Women's experiences of pregnancy related pelvic girdle pain: A systematic review. *Midwifery.* 2018;56:102-111.
25. Liddle SD, Pennick V. Interventions for preventing and treating low-back and pelvic pain during pregnancy. *Cochrane Database Syst Rev.* 2015(9):Cd001139.
26. Gutke A, Betten C, Degerskar K, Pousette S, Fagevik Olsen M. Treatments for pregnancy-related lumbopelvic pain: a systematic review of physiotherapy modalities. *Acta Obstet Gynecol Scand.* 2015;94(11):1156-1167.
27. Keskin EA, Onur O, Keskin HL, Gumus, II, Kafali H, Turhan N. Transcutaneous electrical nerve stimulation improves low back pain during pregnancy. *Gynecol Obstet Invest.* 2012;74(1):76-83.
28. Johnson M. Transcutaneous Electrical Nerve Stimulation: Mechanisms, Clinical Application and Evidence. *Rev Pain.* 2007;1(1):7-11.
29. Lund I, Lundeberg T. Mechanisms of acupuncture. 2016;4(4):26-30.
30. Shah S, Banh ET, Koury K, Bhatia G, Nandi R, Gulur P. Pain Management in Pregnancy: Multimodal Approaches. *Pain Res Treat.* 2015;2015:987483.
31. Carter RE, Lubinsky J. Rehabilitation Research: Principles and Applications. 5th ed. St Louis, MO: Elsevier; 2016:110-111.
32. Gutke A, Kjellby-Wendt G, Oberg B. The inter-rater reliability of a standardised classification system for pregnancy-related lumbopelvic pain. *Man Ther.* 2010;15(1):13-18.
33. Springer BA, Marin R, Cyhan T, Roberts H, Gill NW. Normative values for the unipedal stance test with eyes open and closed. *J Geriatr Phys Ther.* 2007;30(1):8-15.
34. Elden H, Ladfors L, Olsen MF, Ostgaard HC, Hagberg H. Effects of acupuncture and stabilising exercises as adjunct to standard treatment in pregnant women with pelvic girdle pain: randomised single blind controlled trial. *Bmj.* 2005;330(7494):761.
35. Fairbank JC, Pynsent PB. The Oswestry Disability Index. *Spine (Phila Pa 1976).* 2000;25(22):2940-2952; discussion 2952.
36. Westaway MD, Stratford PW, Binkley JM. The patient-specific functional scale: validation of its use in persons with neck dysfunction. *J Orthop Sports Phys Ther.* 1998;27(5):331-338.
37. de Zwart BC, Frings-Dresen MH, van Duivenbooden JC. Test-retest reliability of the Work Ability Index questionnaire. *Occup Med (Lond).* 2002;52(4):177-181.
38. Haakstad LA, Voldner N, Henriksen T, Bo K. Physical activity level and weight gain in a cohort of pregnant Norwegian women. *Acta Obstet Gynecol Scand.* 2007;86(5):559-564.
39. Stuge B, Garratt A, Krogstad Jenssen H, Grotle M. The pelvic girdle questionnaire: a condition-specific instrument for assessing activity limitations and symptoms in people with pelvic girdle pain. *Phys Ther.* 2011;91(7):1096-1108.
40. Gutke A, Stuge B, Elden H, Sandell C, Asplin G, Fagevik Olsen M. The Swedish version of the pelvic girdle questionnaire, cross-cultural adaptation and validation. *Disabil Rehabil.* 2019:1-8.
41. Hjermstad MJ, Fayers PM, Haugen DF, et al. Studies comparing Numerical Rating Scales, Verbal Rating Scales, and Visual Analogue Scales for assessment of pain intensity in adults: a systematic literature review. *J Pain Symptom Manage.* 2011;41(6):1073-1093.
42. Elkins G, Staniunas R, Rajab MH, Marcus J, Snyder T. Use of a numeric visual analog anxiety scale among patients undergoing colorectal surgery. *Clin Nurs Res.* 2004;13(3):237-244.
43. Rabin R, de Charro F. EQ-5D: a measure of health status from the EuroQol Group. *Ann Med.* 2001;33(5):337-343.

For peer review only - http://bmjopen.bmj.com/site/about/guidelines.xhtml

44. Kozinszky Z, Dudas RB. Validation studies of the Edinburgh Postnatal Depression Scale for the antenatal period. *J Affect Disord.* 2015;176:95-105.
45. Rubertsson C, Borjesson K, Berglund A, Josefsson A, Sydsjo G. The Swedish validation of Edinburgh Postnatal Depression Scale (EPDS) during pregnancy. *Nord J Psychiatry.* 2011;65(6):414-418.
46. Rosenstiel AK, Keefe FJ. The use of coping strategies in chronic low back pain patients: Relationship to patient characteristics and current adjustment. *Pain.* 1983;17(1):33-44.
47. Jensen I, Linton S. Coping Strategies Questionnaire (CSQ): Reliability of the Swedish version of the CSQ. *Scandinavian Journal of Behaviour Therapy.* 1993;22:139-145.
48. Horn KK, Jennings S, Richardson G, Vliet DV, Hefford C, Abbott JH. The patient-specific functional scale: psychometrics, clinimetrics, and application as a clinical outcome measure. *J Orthop Sports Phys Ther.* 2012;42(1):30-42.
49. Truong BT, Lupattelli A, Kristensen P, Nordeng H. Sick leave and medication use in pregnancy: A European web-based study. *BMJ Open.* 2017;7(8).
50. Gaston A, Vamos CA. Leisure-time physical activity patterns and correlates among pregnant women in Ontario, Canada. *Matern Child Health J.* 2013;17(3):477-484.
51. Walsh JM, McGowan C, Byrne J, McAuliffe FM. Prevalence of physical activity among healthy pregnant women in Ireland. *Int J Gynaecol Obstet.* 2011;114(2):154-155.
52. Evenson KR, Savitz DA, Huston SL. Leisure-time physical activity among pregnant women in the US. *Paediatr Perinat Epidemiol.* 2004;18(6):400-407.
53. Hegaard HK, Damm P, Hedegaard M, et al. Sports and leisure time physical activity during pregnancy in nulliparous women. *Matern Child Health J.* 2011;15(6):806-813.
54. Ogollah R, Bishop A, Lewis M, Grotle M, Foster NE. Responsiveness and Minimal Important Change for Pain and Disability Outcome Measures in Pregnancy-Related Low Back and Pelvic Girdle Pain. *Phys Ther.* 2019.
55. Olsson C, Nilsson-Wikmar L. Health-related quality of life and physical ability among pregnant women with and without back pain in late pregnancy. *Acta Obstet Gynecol Scand.* 2004;83(4):351-357.
56. Sahrakorpi N, Koivusalo SB, Stach-Lempinen B, Eriksson JG, Kautiainen H, Roine RP. "The Burden of Pregnancy"; heavier for the heaviest? The changes in Health Related Quality of Life (HRQoL) assessed by the 15D instrument during pregnancy and postpartum in different body mass index groups: a longitudinal survey. *Acta Obstet Gynecol Scand.* 2017;96(3):352-358.
57. Blasini M, Peiris N, Wright T, Colloca L. The Role of Patient-Practitioner Relationships in Placebo and Nocebo Phenomena. *Int Rev Neurobiol.* 2018;139:211-231.
58. St-Laurent A, Lardon E, Babineau V, Ruchat SM. Reproductive history, maternal anxiety and past physical activity practice predict physical activity levels throughout pregnancy. *Prev Med Rep.* 2019;16:100992.
59. Morino S, Ishihara M, Umezaki F, et al. Low back pain and causative movements in pregnancy: a prospective cohort study. *BMC Musculoskelet Disord.* 2017;18(1):416.
60. Liebano RE, Rakel B, Vance CGT, Walsh DM, Sluka KA. An investigation of the development of analgesic tolerance to TENS in humans. *Pain.* 2011;152(2):335-342.
61. Johnson MI. Resolving Long-Standing Uncertainty about the Clinical Efficacy of Transcutaneous Electrical Nerve Stimulation (TENS) to Relieve Pain: A Comprehensive Review of Factors Influencing Outcome. *Medicina (Kaunas).* 2021;57(4).

For peer review only - http://bmjopen.bmj.com/site/about/guidelines.xhtml

Figure legends.

Figure 1. Flowchart of the study according to the CONSORT requirements

Figure 2. Reported side effects of the interventions, Acupuncture (n=9/41) and TENS (n=6/45)

For peer review only

For peer review only - http://bmjopen.bmj.com/site/about/guidelines.xhtml

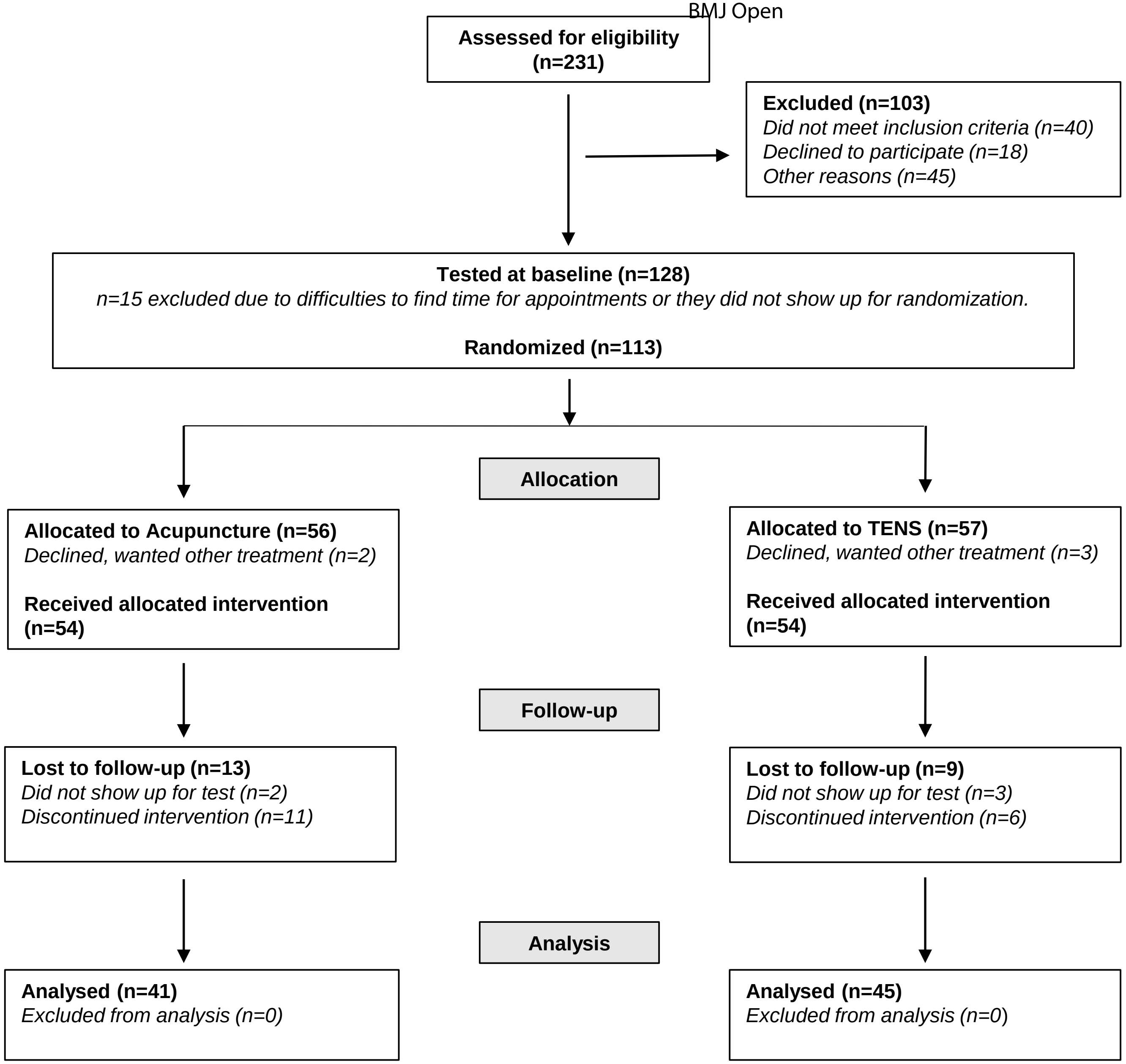
Assessed for eligibility (n=231)
Excluded (n=103)
Did not meet inclusion criteria (n=40)
Declined to participate (n=18)
Other reasons (n=45)
Tested at baseline (n=128)
n=15 excluded due to difficulties to find time for appointments or they did not show up for randomization.
Randomized (n=113)
Allocation
Allocated to Acupuncture (n=56)
Declined, wanted other treatment (n=2)
Received allocated intervention (n=54)
Allocated to TENS (n=57)
Declined, wanted other treatment (n=3)
Received allocated intervention (n=54)
Follow-up
Lost to follow-up (n=13)
Did not show up for test (n=2)
Discontinued intervention (n=11)
Lost to follow-up (n=9)
Did not show up for test (n=3)
Discontinued intervention (n=6)
Analysis
Analysed (n=41)
Excluded from analysis (n=0)
Analysed (n=45)
Excluded from analysis (n=0)

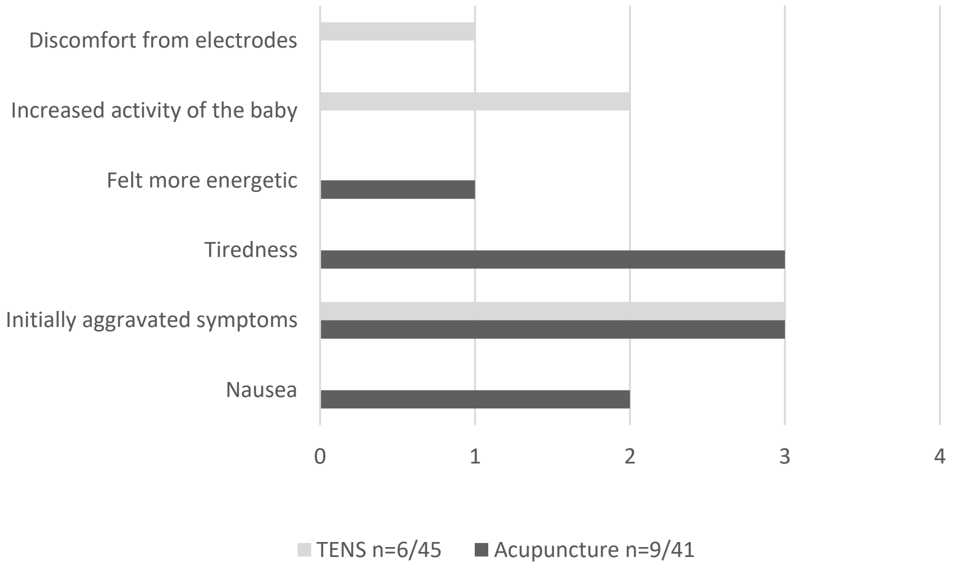
Discomfort from electrodes
Increased activity of the baby
Felt more energetic
Tiredness
Initially aggravated symptoms
Nausea
0
1
2
3
4
TENS n=6/45
Acupuncture n=9/41

STandards for Reporting Interventions in Clinical Trials of Acupuncture (STRICTA): extending the CONSORT Statement

"Maintenance of physical activity level, functioning and health after non-pharmacological treatment of pelvic girdle pain with either transcutaneous electric nerve stimulation or acupuncture. A randomized controlled trial"

Table 1: STRICTA 2010 checklist of information to include when reporting interventions in a clinical trial of acupuncture (Expansion of Item 5 from CONSORT 2010 checklist)

**Item**	**Detail**	**Reported on page no:**
**1. Acupuncture rationale**	1a) Style of acupuncture (e.g. Traditional Chinese Medicine, Japanese, Korean, Western medical, Five Element, ear acupuncture, etc)	08
	1b) Reasoning for treatment provided, based on historical context, literature sources, and/or consensus methods, with references where appropriate	06
	1c) Extent to which treatment was varied	
**2. Details of needling**	2a) Number of needle insertions per subject per session (mean and range where relevant)	08
	2b) Names (or location if no standard name) of points used (uni/bilateral)	08
	2c) Depth of insertion, based on a specified unit of measurement, or on a particular tissue level	08
	2d) Response sought (e.g. *de qi* or muscle twitch response)	08
	2e) Needle stimulation (e.g. manual, electrical)	08
	2f) Needle retention time	08
	2g) Needle type (diameter, length, and manufacturer or material)	08
**3. Treatment regimen**	3a) Number of treatment sessions	08
	3b) Frequency and duration of treatment sessions	08
**4. Other components of treatment**	4a) Details of other interventions administered to the acupuncture group (e.g. moxibustion, cupping, herbs, exercises, lifestyle advice)	08
	4b) Setting and context of treatment, including instructions to practitioners, and information and explanations to patients	
**5. Practitioner background**	5) Description of participating acupuncturists (qualification or professional affiliation, years in acupuncture practice, other relevant experience)	08
**6. Control or comparator interventions**	6a) Rationale for the control or comparator in the context of the research question, with sources that justify this choice	06
	6b) Precise description of the control or comparator. If sham acupuncture or any other type of acupuncture-like control is used, provide details as for Items 1 to 3 above.	09

Note: This checklist, which should be read in conjunction with the explanations of the STRICTA items provided in the main text, is designed to replace CONSORT 2010's item 5 when reporting an acupuncture trial.

Citation: *MacPherson H, Altman DG, Hammerschlag R, Youping L, Taixiang W, White A, Moher D; STRICTA Revision Group. Revised STandards for Reporting Interventions in Clinical Trials of Acupuncture (STRICTA): extending the CONSORT statement. PLoS Med. 2010 Jun 8;7(6):e1000261*

For peer review only

STandards for Reporting Interventions in Clinical Trials of Acupuncture (STRICTA): extending the CONSORT Statement

Table 2: CONSORT 2010 checklist with the Non-pharmacological Trials Extension to CONSORT (with STRICTA 2010 extending CONSORT Item 5 for acupuncture trials)

Section/Topic	Item #	CONSORT 2010 Statement*: Checklist item[10]. Describe:	Additional items from the Non-pharmacological Trials Extension to CONSORT[14]. Add:	Reported on page no:
TITLE AND ABSTRACT				
	1.a	Identification as a randomized trial in the title	In the abstract, description of the experimental treatment, comparator, care providers, centres and blinding status.	01
	1.b	Structured summary of trial design, methods, results, and conclusions; for specific guidance see CONSORT for Abstracts [58,59]		02–03
INTRODUCTION				
Background and objectives	2.a	Scientific background and explanation of rationale		03–04
	2.b	Specific objectives or hypotheses		03–04
METHODS				
Trial design	3.a	Description of trial design (e.g., parallel, factorial) including allocation ratio		06–07
	3.b	Important changes to methods after trial commencement (e.g. eligibility criteria), with reasons		n a
Participants	4.a	Eligibility criteria for participants	When applicable, eligibility criteria for centers and those performing the interventions.	07
	4.b	Settings and locations where the data were collected		07
**Interventions**	**5**	**The interventions for each group with sufficient details to allow replication, including how and when they were actually administered**	Precise details of both the experimental treatment and comparator - see Table 1 for details	**Details described in table 1**
Outcomes	6.a	Completely defined pre-specified primary and secondary outcome measures, including how and when they were assessed		09–10
	6.b	Any changes to trial outcomes after the trial commenced with reasons		n a
Sample size	7.a	How sample size was determined	When applicable, details of whether and how the clustering by care providers or centers was addressed.	10
	7.b	When applicable, explanation of any interim analyses and stopping guidelines		n a

For peer review only

STandards for Reporting Interventions in Clinical Trials of Acupuncture (STRICTA): extending the CONSORT Statement

Section/Topic	Item #	CONSORT 2010 Statement*: Checklist item[10]. Describe:	Additional items from the Non-pharmacological Trials Extension to CONSORT[14]. Add:	Reported on page no:
Randomization				
Sequence generation	8.a	Method used to generate the random allocation sequence	When applicable, how care providers were allocated to each trial group.	08
	8.b	Type of randomization; details of any restriction (e.g., blocking and block size)		08
Allocation concealment	9	Mechanism used to implement the random allocation sequence (e.g., sequentially numbered containers), describing any steps taken to conceal the sequence until interventions were assigned		08
Implementation	10	Who generated the random allocation sequence, who enrolled participants, and who assigned participants to interventions		08
Blinding	11.a	If done, who was blinded after assignment to interventions (e.g. participants, care providers, those assessing outcomes) and how	Whether or not those administering co-interventions were blinded to group assignment. If blinded, method of blinding and description of the similarity of interventions.	10
	11.b	If relevant, description of the similarity of interventions		n a
Statistical methods	12.a	Statistical methods used to compare groups for primary and secondary outcomes	When applicable, details of whether and how the clustering by care providers or centers was addressed.	10–11
	12.b	Methods for additional analyses, such as subgroup analyses and adjusted analyses		10–11
RESULTS				
Participant flow (A diagram is strongly recommended)	13.a	For each group, the numbers of participants who were randomly assigned, received intended treatment, and were analyzed for the primary outcome	The number of care providers or centers performing the intervention in each group and the number of patients treated by each care provider or in each center.	11, fig. 1
	13.b	For each group, losses and exclusions after randomization, together with reasons		11, fig. 1
Implementation of intervention			Details of the experimental treatment and comparator as they were implemented.	13
Recruitment	14.a	Dates defining the periods of recruitment and follow-up		7–8

STandards for Reporting Interventions in Clinical Trials of Acupuncture (STRICTA): extending the CONSORT Statement

Section/Topic	Item #	CONSORT 2010 Statement*: Checklist item[10]. Describe:	Additional items from the Non-pharmacological Trials Extension to CONSORT[14]. Add:	Reported on page no:
	14.b	Why the trial ended or was stopped		n a
Baseline data	15	A table showing baseline demographic and clinical characteristics for each group	When applicable, a description of care providers (case volume, qualification, expertise, etc.) and centers (volume) in each group.	11, table 1
Numbers analyzed	16	For each group, number of participants (denominator) included in each analysis and whether the analysis was by original assigned groups		Table 3, 4
Outcomes and estimation	17.a	For each primary and secondary outcome, results for each group, and the estimated effect size and its precision (e.g., 95% confidence interval)		Table 3, 4
	17.b	For binary outcomes, presentation of both absolute and relative effect sizes is recommended		Table 3, 4
Ancillary analyses	18	Results of any other analyses performed, including subgroup analyses and adjusted analyses, distinguishing pre-specified from exploratory		n a
Harms	19	All important harms or unintended effects in each group; for specific guidance see CONSORT for Harms [60]		13, fig. 2
DISCUSSION				
Limitations	20	Trial limitations, addressing sources of potential bias, imprecision, and, if relevant, multiplicity of analyses		17–20
Generalizability	21	Generalizability (external validity, applicability) of the trial findings	Generalizability (external validity) of the trial findings according to the intervention, comparators, patients and care providers and centers involved in the trial.	17–20

For peer review only

STandards for Reporting Interventions in Clinical Trials of Acupuncture (STRICTA): extending the CONSORT Statement

Section/Topic	Item #	CONSORT 2010 Statement*: Checklist item[10]. Describe:	Additional items from the Non-pharmacological Trials Extension to CONSORT[14]. Add:	Reported on page no:
Interpretation	22	Interpretation consistent with results, balancing benefits and harms, and considering other relevant evidence	In addition, take into account the choice of the comparator, lack of or partial blinding, unequal expertise of care providers or centers in each group.	20
OTHER INFORMATION				
Registration	23	Registration number and name of trial registry		03, 21
Protocol	24	Where the full trial protocol can be accessed, if available		n a
Funding	25	Sources of funding and other support (e.g., supply of drugs); role of funders		21

* We strongly recommend reading this Statement in conjunction with the CONSORT 2010 explanation and elaboration [11] for important clarifications on all the items. If relevant, we also recommend reading CONSORT extensions for cluster randomized trials [61], noninferiority and equivalence trials [62], herbal interventions [63], and pragmatic trials [16]. Moreover, additional extensions are forthcoming. For those and also for up-to-date references relevant to this checklist, see http://www.consort-statement.org.

For peer review only
